# Invasion Patterns and Niche Dynamics of the Pollinivorous Florida Calligrapher, *Toxomerus floralis* (Diptera: Syrphidae) in the Afrotropical Region

**DOI:** 10.1002/ece3.73838

**Published:** 2026-06-23

**Authors:** Burgert Muller, John Midgley, Georg Goergen, Ali Al Jahdhami, Michelson Azo'o Ela, Terence Bellingan, Simon Cavaillès, Robert Copeland, Marc De Meyer, Martin Hauser, Allen Holmes, Ximo Mengual, Gabriel Nève, Menno Reemer, Jeff Skevington, Gunilla Ståhls, Eugène Sinzinkayo, John Smit, Axel Ssymank, Genevieve Theron, Kurt Jordaens

**Affiliations:** ^1^ National Museum Bloemfontein Bloemfontein South Africa; ^2^ Department Natural Sciences KwaZulu‐Natal Museum Pietermaritzburg South Africa; ^3^ Department of Zoology and Entomology Rhodes University Makhanda South Africa; ^4^ International Institute for Tropical Agriculture Biodiversity Centre Cotonou Benin; ^5^ Ministry of Agriculture, Fisheries and Water Resource Samed Ashan Oman; ^6^ Department of Biological Sciences University of Maroua Maroua Cameroon; ^7^ Department of Entomology and Arachnology Albany Museum Makhanda South Africa; ^8^ Bel‐Air Trévérec France; ^9^ Biosystematics Unit International Centre of Insect Physiology and Ecology Nairobi Kenya; ^10^ Invertebrates Section and JEMU Royal Museum for Central Africa Tervuren Belgium; ^11^ Plant Pest Diagnostics Branch California Department of Food and Agriculture Sacramento California USA; ^12^ Independent Researcher Burnley Lancashire UK; ^13^ Museum Koenig Bonn Leibniz‐Institut zur Analyse des Biodiversitätswandels Bonn Germany; ^14^ IMBE Aix Marseille Univ, Avignon Univ, CNRS, IRD Marseille France; ^15^ Naturalis Biodiversity Center Leiden the Netherlands; ^16^ Agriculture and Agri‐Food Canada Canadian National Collection of Insects, Arachnids and Nematodes Ottawa Ontario Canada; ^17^ Finnish Museum of Natural History Luomus University of Helsinki Helsinki Finland; ^18^ Burundian Office for the Protection of the Environment Biodiversity Research Service Bujumbura Burundi; ^19^ Independent Researcher Wachtberg Germany

**Keywords:** Afrotropical Region, alien species, flower fly, niche analysis, niche expansion

## Abstract

The rapid spread of *Toxomerus floralis* (Fabricius, 1798) (Diptera: Syrphidae) within the Afrotropical region is described. We characterise and compare the climatic niches of *T. floralis* in its native (Southern North America, Central America and South America) and invaded (Afrotropical Region) range to assess the potential for further expansion across Africa and beyond, and included future global climate models and socioeconomic pathways as projections. Occurrence data for native and invaded ranges were obtained from field sampling by authors, major collections of Afrotropical Syrphidae, collections records and occurrence data from the Global Biodiversity Information Facility (GBIF), including iNaturalist data. Single and ensemble species distribution modelling was performed utilising the ‘biomod2’ package, and the ‘ecospat’ package was used to determine the Continuous Boyce Index and niche dynamics of the species. Current and global climate models (GCM) Worldclim 2.1 data were used as environmental variables. Ensemble models showed high predictive accuracy (native: TSS = 0.824, CBI *R*_s_ = 0.982; expanded: TSS = 0.805, CBI *R*_s_ = 0.997), Bio18 and Bio2 Worldclim 2.1 variables proving the most important predictors. Niche dynamics showed primarily niche conservatism (76.4%) as well as a degree of expansion (23.6%, *p* = 0.048). Models predict high probability of further spread throughout Africa, with potential expansion into other Ecoregions. Future climate projections suggest continued range expansion through 2100 under most scenarios. The species' potential distribution shows spatial overlap with its larval host plants, however, since host distributions were not integrated into the modelling framework, the relative roles of climatic and biotic factors in limiting distribution cannot be directly evaluated from current analyses. It is predicted that *T. floralis* will invade the tropical regions of Asia and Australia in the near future. Citizen science data proved invaluable for tracking the expansion of *T. floralis* , highlighting the value of such platforms for monitoring non‐native species expansions.

## Graphical abstract

The rapid spread of *Toxomerus* *floralis* (Fabricius, 1798) (Diptera: Syrphidae) within the Afrotropical region is analysed using occurrence data from field sampling, museum collections and citizen science sources, combined with single and ensemble species distribution models. Comparisons of expanded niches between native (Americas) and invaded (Afrotropical) ranges, incorporating current and future climate projections, reveal high model performance, primarily niche conservatism (76.4%), as well as a degree of expansion (23.6%, p = 0.048) and identify key climatic predictors limiting distribution. Models predict continued expansion across Africa and likely invasion of tropical regions of Asia and Australia, demonstrating both adaptive capacity and the value of citizen science data for monitoring biological invasions.

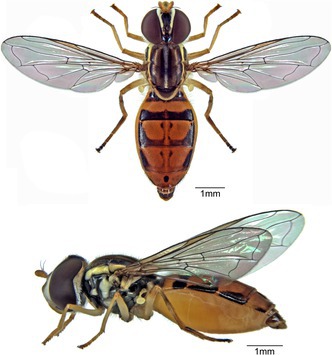

## 1 Introduction

The invasive movement of non‐native species is a global phenomenon and one of the most important threats to biodiversity (e.g., Gurevitch and Padilla 2004; Roy et al. 2014). To mitigate undesirable consequences of invasive alien species, such as negative impacts on native ecosystems (Thuiller et al. 2005) we require knowledge on the ecology and behaviour of the species in the recipient invaded region. This also comprises a sound understanding of its spread and habitat requirements. This knowledge may be gained by monitoring the spread of the species, and using this information to refine predictive modelling tools that are useful for guiding preventive and management actions (Hulme 2006).

A first step to predict the pathway by which invasive species (*sensu* Richardson et al. 2000) may colonise new environments is identifying the biotic (i.e., hosts, predators, competition) and abiotic (environmental, i.e., climatic, geographic or ecological) variables that account for the species' distribution in its native range. Often, the success of a species expanding its range depends on which environmental conditions it experiences outside its native range. Areas outside the native range that have similar environmental conditions will be more likely to be colonised (the ‘climate match hypothesis’; Peterson and Robins 2003; Broennimann et al. 2007; Jiménez‐Valverde et al. 2011).

**FIGURE 1:**
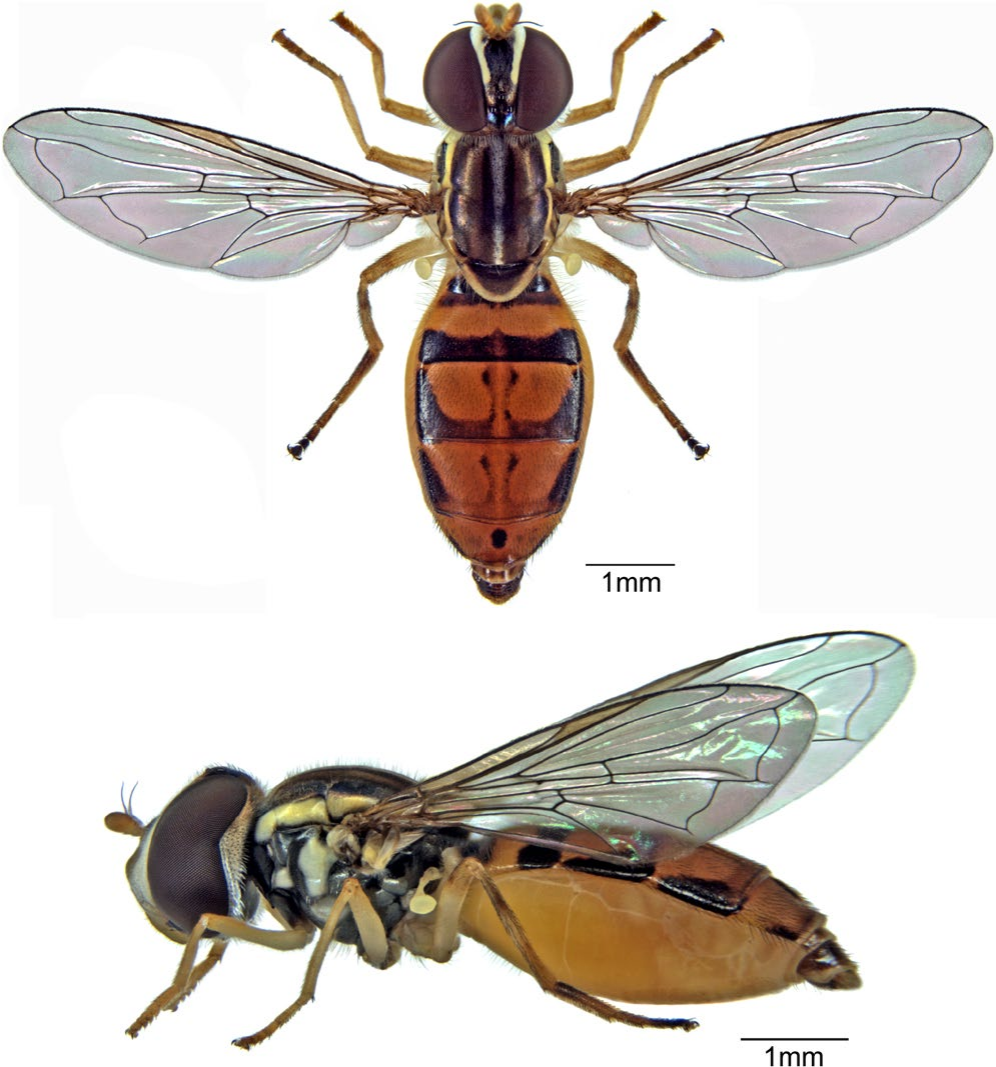
Adult *Toxomerus floralis* from the Afrotropical region.

Niche shifts are detected by comparing the environmental space occupied by a species in its native range with that occupied in its invaded range. While most invasive species conserve their native ecological niche even in the invaded area (‘niche conservatism’; see Wiens and Graham 2005; Pearman et al. 2008), some species seem able to adapt to new environmental conditions by shifting their native niche (e.g., Broennimann et al. 2012; Guisan et al. 2014; Ørsted and Ørsted 2019). Such shifts can be broadly distinguished between apparent and true niche shifts. Apparent shifts may occur when a species expands into environmental conditions that were accessible but not occupied in its native range (Keane and Crawley 2002; Oliveira et al. 2018), or when biotic factors differ between ranges, that is, lack of natural enemies or interspecific competition, or the addition of anthropogenic disturbances, rather than reflecting a true change in environmental tolerance (Feng et al. 2024). True niche shifts, by contrast, may be a consequence of rapid evolutionary processes acting on species under new selective pressures (Broennimann et al. 2007), and have been described at early stages of invasion for several species (Early and Sax 2014; González‐Moreno et al. 2015). Insects are one of the largest groups of invaders (Turner et al. 2021). Some ecologically and biologically well‐known insect groups, such as hover flies (Diptera, Syrphidae), provide ideal case studies to evaluate the effects of environmental or climatic changes (Thomas 2005) on insect distribution and potential new niche utilisation, because of their quick ecological or physical responses. Introductions of hover fly species from the Palaearctic Region, especially into the Nearctic Region, are well‐documented. For instance, more than 10 European species have been introduced into the USA and some of these (e.g., *Eristalis tenax* (Linnaeus, 1758), *Eristalis arbustorum* (Linnaeus, 1758), *Eristalinus aeneus* (Scopoli, 1763) and *Syritta pipiens* (Linnaeus, 1758)) may be found throughout the entire USA (Skevington et al. 2019). The European drone fly, *Eristalis arbustorum* , has additionally also been introduced into Australia and New Zealand and the common bulb fly, *Merodon equestris* (Fabricius, 1794), has been introduced into New Zealand and is considered of economic importance (Environmental Protection Authority 2025). Other well‐known examples of hover fly introductions are the introduction of the Afrotropical/Palaearctic species *Eristalinus taeniops* (Wiedemann, 1818) into the Nearctic Region, the Palaearctic species *Eristalis tenax* into the Afrotropical region and Australia [Cook et al. (2020) mention that *E. tenax* is beneficial in pollinating field crops in Australia], and the Neotropical species *Ornidia obesa* (Fabricius, 1775) and *Toxomerus floralis* (Fabricius, 1798) (Figure 1) into the Afrotropical region (Jordaens et al. 2015; Ramage et al. 2018; Kondo et al. 2024). However, to our knowledge, the invasive potential and potential niche‐shifts have never been studied in detail for any of these species, thereby leaving us with an incomplete understanding of the ecology, distribution and niche dynamics in invaded ranges of introduced hover flies.

**FIGURE 2:**
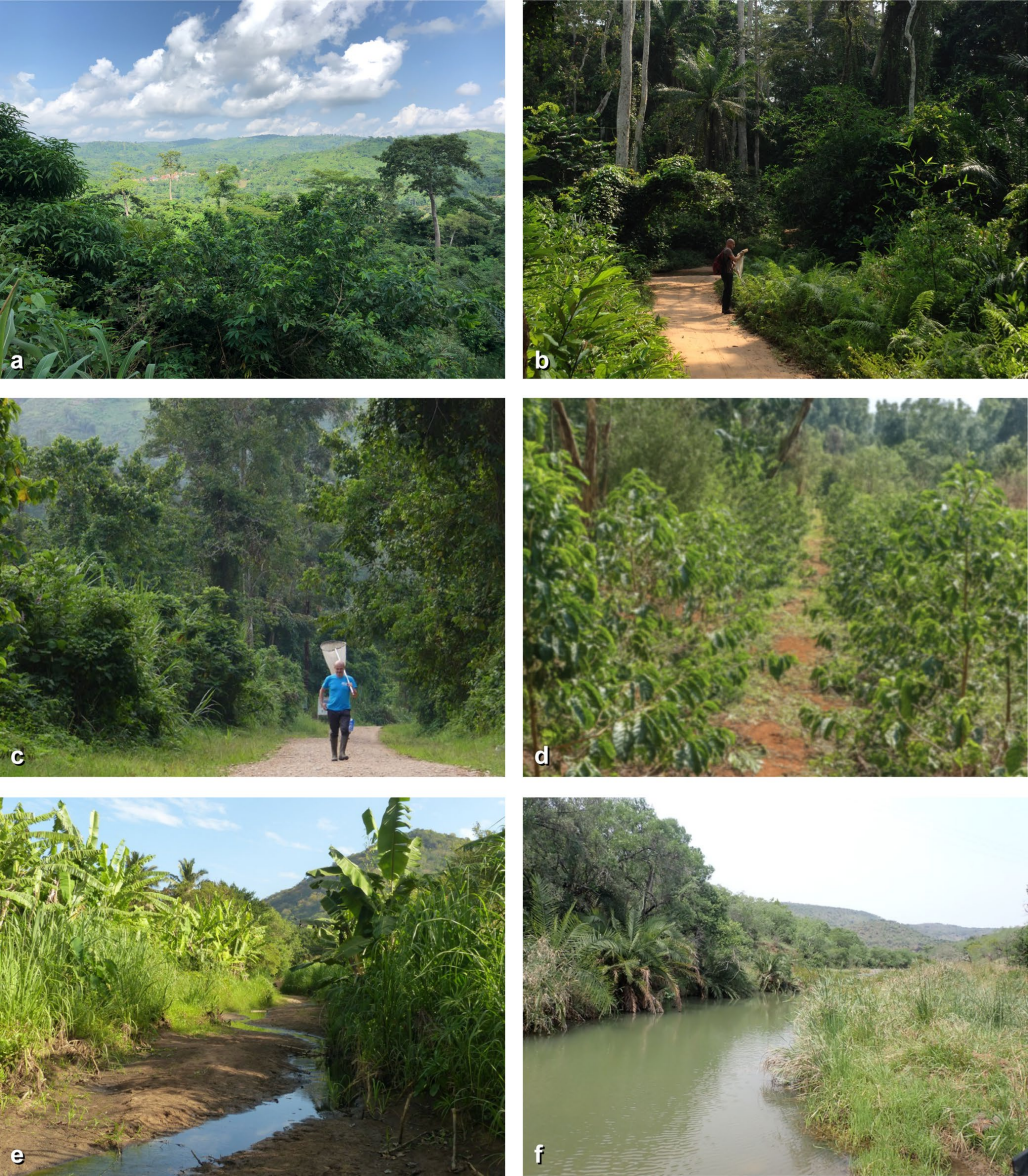
Some of the Afrotropical localities where *Toxomerus floralis* was collected. (a) Kloto forest, Togo KMMA, Kurt Jordaens. (b) Pobè, Benin KMMA, Kurt Jordaens. (c) Bwindi Impenetrable Forest National Park, Uganda KMMA, Kurt Jordaens. (d) Kasarani, Kenya KMMA, Kurt Jordaens. (e) Msau, Kenya, Jeffrey H. Skevington. (f) Ithala Game Reserve, South Africa KMMA, Kurt Jordaens.

**FIGURE 3:**
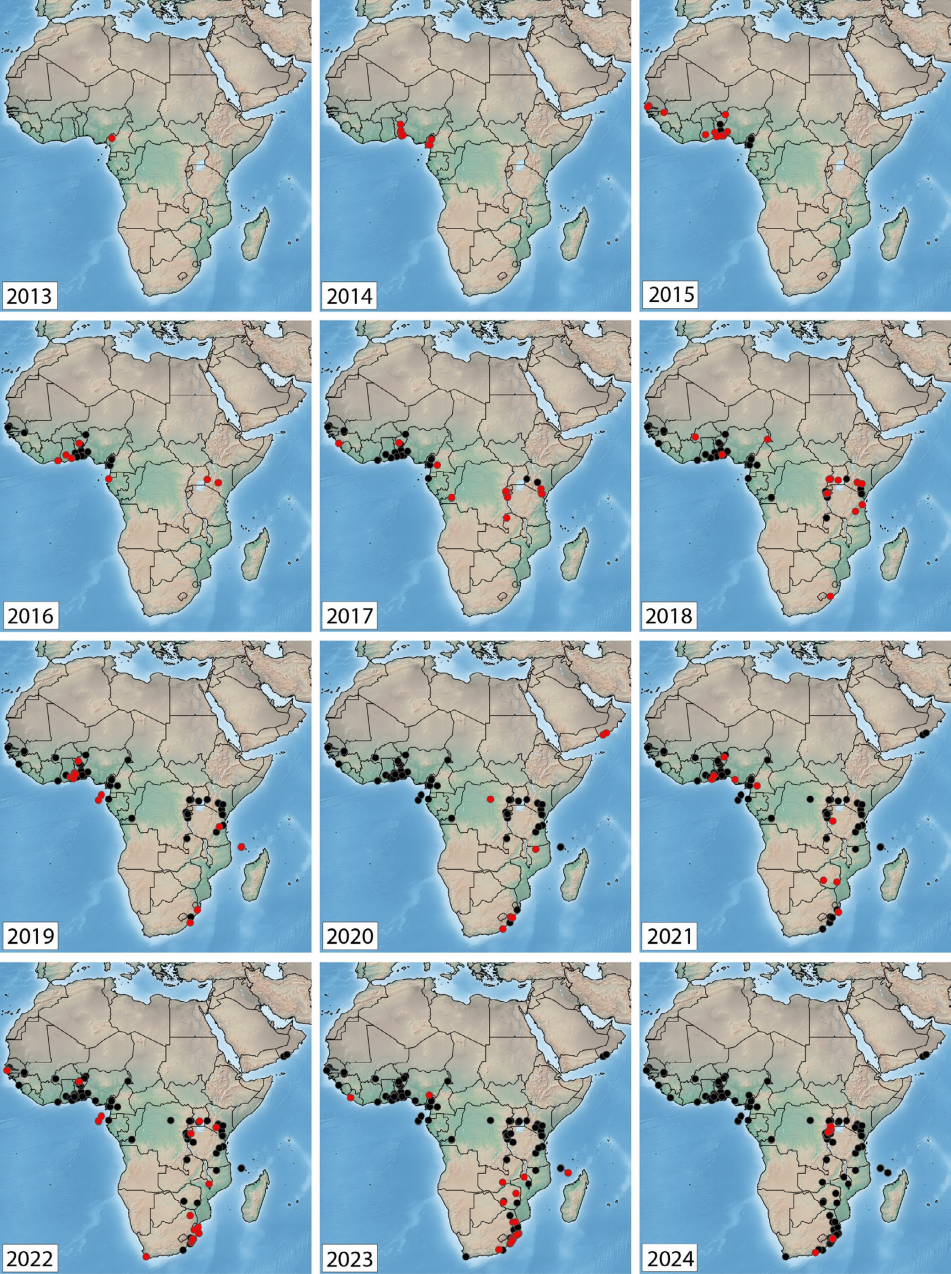
Temporal spread of the hover fly *Toxomerus floralis* in the Afrotropical region from December 2013–May 2024. Red dots represent new localities for that year; black dots are localities of previous years. Created in SimpleMappr (Shorthouse 2010).

The genus *Toxomerus* is one of the most speciose genera of Syrphidae and currently comprises more than 150 species confined to the New World (southern Canada to southern Chile and Argentina) (Thompson and Thompson 2006; Borges and Couri 2009). The majority of species are predatory, and apart from *T. floralis* , only *T. apegiensis* (Harbach, 1974) and *T. politus* (Say, 1823) are known pollen feeders, however, *T. politus* is also known to feed on the leaves and stalks of maize plants, with mouthparts not modified specifically for pollen feeding (Reemer and Rotheray 2009). The native distribution of *T. floralis* ranges from the USA (Texas and Florida) to southern South America (Thompson and Thompson 2006; Borges and Couri 2009; Thompson 2013). The species was recently introduced into the Afrotropical region (Figure 2) with the first records from Cameroon (2013), Benin (2014), Togo and Nigeria (both 2015). *Toxomerus floralis* is only the second known introduction of a hover fly from the New World into the Afrotropical region, with *O. obesa* being the first. The date and place of introduction remain unknown, as most regions of the Afrotropics have been inadequately sampled for Syrphidae, but the absence of records of *T. floralis* in the literature and the absence of the species in museum collections worldwide before 2013 (see Section 2) suggest that the introduction is recent (see Figure 3).

Invasive alien plants threaten biodiversity, ecosystems and service provision worldwide (Vilà et al. 2011). They can have positive and negative direct and indirect effects on herbivorous insects, including those that provide pollination services such as hover flies. Interestingly, the larvae of *T. floralis* feed on pollen from two such invasive plants: *Cyperus rotundus* L. and *Mitracarpus hirtus* (L.) D.C.

*Cyperus rotundus* (coco‐grass, Java grass, nut grass, purple nut sedge, red nut sedge) is a species of sedge (Cyperaceae) native to Africa, southern and central Europe (north to France and Austria), southern Asia and Australia. It has been introduced into other parts of Europe (e.g., The Netherlands, Czech Republic and the UK), the United States (from Florida north to New York and Minnesota and west to California and most of the states in between), and Central and South America where it is widespread (see https://powo.science.kew.org/taxon/urn:lsid:ipni.org:names:305797‐1; accessed on 29 July 2025). In several countries (both in its native and invaded ranges) the plant is described as one of the most invasive agricultural weeds, having spread worldwide in both tropical and temperate regions. It is known as a weed in over 90 countries, and affects over 50 cropping systems worldwide (Omezine and Harzallah‐Skhili 2009). Its capability to adapt to a wide range of environmental conditions, along with its aggressive growth habit and prolific reproduction, makes it a problematic weed in agricultural and non‐agricultural lands. It prefers dry conditions, but will tolerate moist soils, and often grows in wastelands and in crop fields. Similarly, *Mitracarpus hirtus* (girdlepod or small squareweed) is a widespread invasive, being an erect or spreading annual plant in the coffee family, Rubiaceae. Its native range extends from Mexico to Tropical South America, growing primarily in the seasonally dry tropical biome. It has been introduced into most of the Afrotropical, Indomalayan and parts of Australasian Regions (see https://powo.science.kew.org/taxon/urn:lsid:ipni.org:names:305797‐1; accessed on 29 July 2025).

The widespread occurrence of *C. rotundus* and *M. hirtus* , both of which are known to endure poor environmental and climatic conditions, may have provided a biological foundation For *T. floralis* in the Afrotropical region (Jordaens et al. 2015). However, whether host availability imposes additional constraints beyond climate could not be assessed, as hostplant layers were not incorporated as distribution rasters in the current study.

Here, we first characterised and compared the climatic niches of the hover fly *T. floralis* (Diptera: Syrphidae) in its native and invaded range to assess the potential for further expansion across Africa and beyond. Occurrence data of the species in the Afrotropical region were used to examine the spatial expansion dynamics of its range. This work allowed us to identify key environmental factors of the ecological niches of *T. floralis* in both its native and expanded range and provides insight into the spread of the species in the Afrotropical region, its potential spread into the Eastern Palaearctic, Indomalayan and Australasian Regions, and the association between the species' distribution and that of the larval food plants.

## 2 Materials and Methods

### 2.1 *Toxomerus floralis* Occurrence Data

To ensure the availability of sufficient data for model generation, occurrence data for native and invaded ranges were obtained from field sampling by authors, visiting the major recent collections of Afrotropical Syrphidae (Data S1) data mining of collections records, and extracting occurrence data from the Global Biodiversity Information Facility (GBIF), which also incorporates iNaturalist data. For the latter, occurrences were confirmed by identifying the pictures uploaded on iNaturalist (https://www.inaturalist.org/).

The GBIF data in the form of a DwC‐A (Darwin Core Archive) were downloaded (GBIF 2025) and occurrence records were filtered to include only those containing valid geographic coordinates and having an associated coordinate uncertainty of no more than 10 km. This was done to ensure that the spatial resolution of the records do not exceed that of the environmental variables used (5 arc minutes, equaling 9.28 km at equator), otherwise resulting in potentially erroneous models (Araújo et al. 2019; Feng et al. 2019). This filtered the original GBIF dataset down from 1229 to 521 records. The accessible area for native (*x*_min_: −129.3333, *x*_max_: −27.0000, *y*_min_: −60.3333, *y*_max_: 40.3333) and invaded (*x*_min_: −23.750, *x*_max_: 69.250, *y*_min_: −42.6667, *y*_max_: 40.8333) ranges are based on the extent of the spatial rasters used in analyses. The extents crop the native area to essentially the southern USA down to South American continent, and the invaded area to mostly Africa and the Arabian Peninsula. The extents also roughly follow the distribution on hostplants *C. rotundus* and *M. hirtus* in combination with known occurrences of *T. floralis* .

Additionally, a geospatial analysis was performed on the records using QGIS 3.28.6 to ensure that all points fall within the aforementioned bounds of the native and invaded ranges.

All records from the various sources above were combined, resulting in 451 records from the native range in the Americas and 181 invaded range records from Africa. To further reduce potential spatial autocorrelation among occurrence data, the R (R Core Team 2017) package ‘spThin’ (Aiello‐Lammens et al. 2015) was used to perform spatial thinning of the data at 10 km, repeating the thinning process 100 times on random selections to ensure that the final thinned dataset is more representative of the total distribution. The final thinned dataset was then subdivided into three occurrence ranges: native (235 records), invaded (128 records) and expanded (native and invaded records combined: 363 records). Figure S1 shows the final collection of occurrence records.

### 2.2 *Cyperus rotundus* and *Mitracarpus hirtus* Occurrence Data

Occurrence data for *Mitracarpus hirtus* (GBIF 2024a) and *Cyperus rotundus* (GBIF 2024b) were downloaded from GBIF. The data were cleaned and any records with geospatial issues were removed. The data were not thinned for autocorrelation as the data were not used for analyses but only for visual comparison. These were used to map known occurrences for both species for comparison to *T. floralis* and its modelled projected distributions (see Figures S2 and S3).

### 2.3 Environmental Variables

**FIGURE 4:**
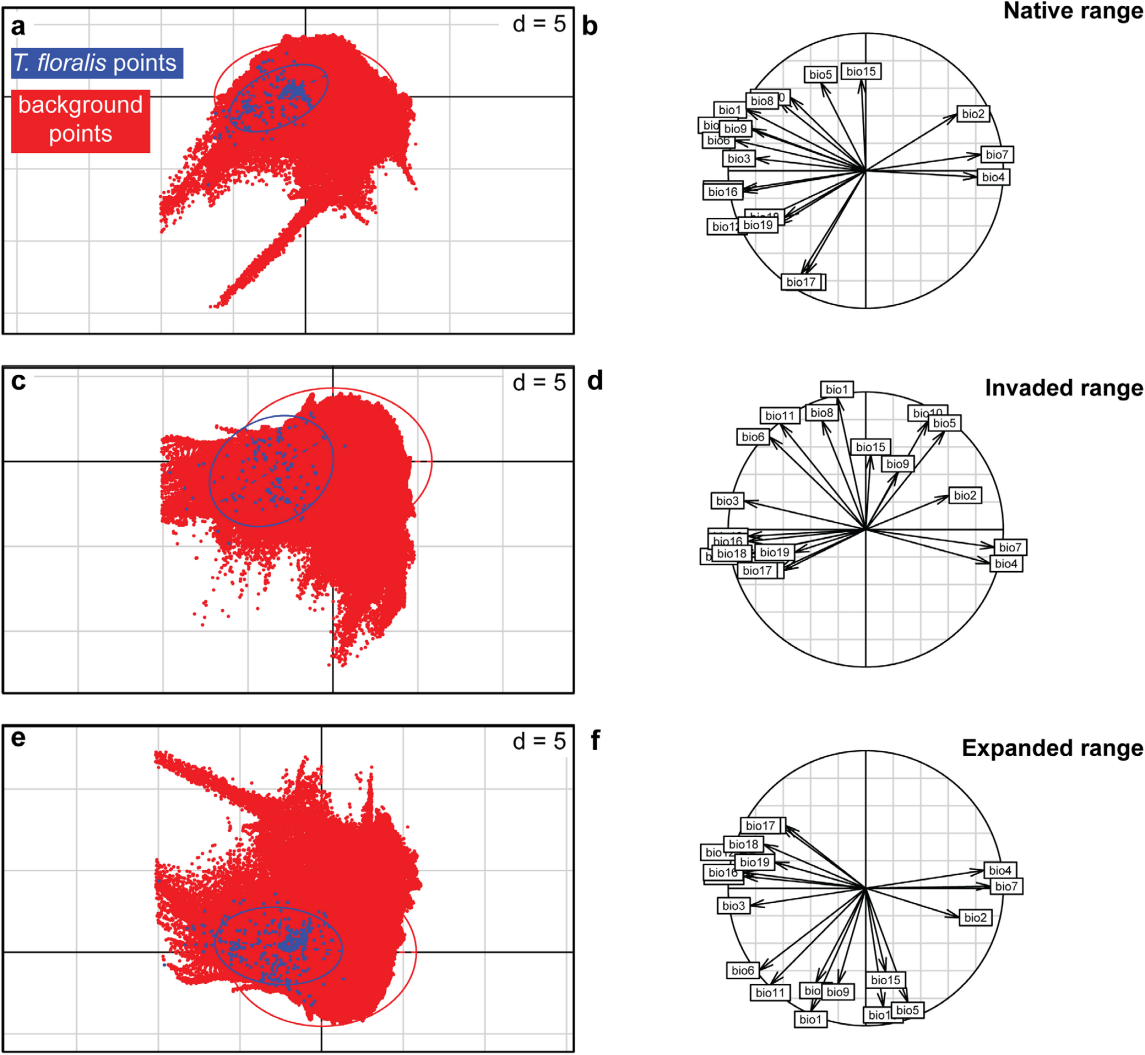
Duality diagram (a, c, e) and Principal Component Analyses of 19 bioclimatic variables of (b, d, f) occupied climatic space by *Toxomerus floralis* within the (a, b) native range (c, d) invaded range and (e, f) expanded range.

Only climatic variables were used in the current study. The climate data used initially consisted of 19 Worldclim 2.1 bioclimatic variables (Data S2) at a spatial resolution of 5 min (Fick and Hijmans 2017), representing ‘current’ climatic conditions from 1970 to 2000. However, using highly correlated variables can have unforeseen effects on the outcomes and performance of the resulting models, and can make interpreting the response curves of potentially important predictive variables especially difficult (Sillero and Barbosa 2020). Therefore, a principal component analysis (PCA) was performed to reduce the dimensionality of the 19 bioclimatic variables, and to determine orthogonality among them, in conjunction with a duality diagram to visualise the climatic space occupied by *T. floralis* (Figure 4). This analysis was performed separately for the assumed native range, invaded range and also for a combined native and invaded range (representing the expanded range which is used to predict the potential climatic niche) of *T. floralis* . These analyses were performed using the ‘dudi.pca()’ function from the ‘ade4’ package (Dray and Dufour 2007). After each initial run, any outliers were removed and the PCA was performed again. The occurrence data of *T. floralis* for each of the three ranges were then projected onto the first two principal component axes (PC1 and PC2, Figure 4a,c,e), which together capture the greatest portion of total climatic variance and allow for environmental space comparisons (Guisan et al. 2017). The horizontal (PC1) and vertical (PC2) axes therefore represent no individual variable but rather gradients of climatic variation. The duality diagrams (Figure 4b,d,f) display the loadings of each bioclimatic variable as arrows projected onto the same PC1–PC2 space, with the arrow direction indicating the variables' contribution to each axis, and the length of the arrow showing the strength of the contribution. The degree of angular separation between arrows indicates the level of co‐linearity, whereby arrows pointing in the same direction are considered positively correlated, those that are orthogonal to one another are considered uncorrelated and those in opposite directions are considered negatively correlated. These analyses allow for a visual representation and interpretation of co‐linearity within the variables.

Additionally, the Variable Inflation Factor (VIF), using the ‘usdm’ package (Naimi et al. 2014), was determined for each of the 19 bioclimatic variables within all three aforementioned geographic ranges to confirm that the variables selected to perform the PCA did, in fact, have low collinearity. The VIF of the variables was calculated pairwise and compared using a maximum linear correlation threshold of 0.9 together with a stepwise comparison with a threshold of 10 (Data S3). Ultimately five variables were retained: Bio18 (Precipitation of Warmest Quarter), Bio15 (Precipitation Seasonality [Coefficient of Variation]), Bio8 (Mean Temperature of Wettest Quarter), Bio3 (Isothermality) and Bio2 (Mean Diurnal Range). These were then combined into raster stacks for use during the modelling process.

### 2.4 Generation of Pseudo‐Absence Data

The final thinned occurrence datasets were used to generate data frames in R that contain a combination of presence data, pseudo‐absence data and the above retained bioclimatic variables using the ‘BIOMOD_FormatingData’ function of the ‘biomod2’ package (Guéguen et al. 2025). The bioclimatic variables used in generating pseudo‐absences were all restricted to the extents of the defined accessible areas of the native and invaded ranges. This allows for the generation of pseudo‐absence data that fall within the constraints of the defined accessible areas and avoids generating data that fall too far outside of areas of realistic dispersal. The recommendation of the ‘biomod2’ documentation was followed and a number three times that of the presence data points was used to generate pseudo‐absence data, namely 705 for native, 384 for invaded and a total of 1089 for expanded ranges.

Background selection has well‐documented consequences for niche comparison and model transferability (Barve et al. 2011; Merow et al. 2013). For the current study, the ‘random’ strategy was followed to generate pseudo‐absence or background points, whereby the random points are sampled within the study area that excludes presence points, but still fall within the defined accessible areas for native and invaded (and by combination the expanded) ranges. A random strategy was preferred over surface range envelope or disk‐based (buffered) approaches, as the fundamental niche of the species is not at present fully understood, and its spread has not seemingly stabilised, which makes any environmental or geographical constrained pseudo‐absence generation potentially misleading. By restricting random generation to the defined accessible areas of native and invaded ranges, the background is constrained to plausible climatic conditions, reducing the risk of sampling climatically irrelevant conditions affecting the accuracy of predictions and potentially introducing noise into model training. Additionally, transferability of the models to future projections may be influenced by how well the generated background points capture the climatic range of the species, making background selection an important step.

### 2.5 Traditional Modelling

The ‘BIOMOD_Modeling’ function of the ‘biomod2’ package was used to generate the predictive models. All 12 modelling algorithms (Data S4) available within the package were run. As an ensemble modelling approach would be followed, inclusion of all of the algorithms in the initial evaluation was seen as the best approach by the authors as only the best performing models (as determined by predetermined thresholds, see below) and iterations of each model would be included in the resulting ensemble model.

Models for native range and expanded range were run for comparison. A randomised cross‐validation strategy was followed with 10 validation sets for each of the models for every set of pseudo‐absence (PA) data, including a combined run, totaling 1332 models being created. An 80/20 split for model training and validation was used, with three permutations for each bioclimatic variable to measure variable importance. Evaluation metrics used to measure model performance were true skill statistic (TSS), receiver operating characteristic (ROC) and Cohen's Kappa coefficient (Kappa). While ROC is the more traditional and threshold independent indicator of model performance, it does not measure positive or negative predictive value (Chicco and Jurman 2023), thus using it in isolation is not best practice. Similarly, Kappa is widely used for presence‐absence models, but is sensitive to species prevalence and regional differences (Allouche et al. 2006). TSS avoids these limitations and is considered to be one of the most practical methods of evaluating species distribution models based on machine learning (Allouche et al. 2006; Yoon and Lee 2023). Given these differences between the metrics, TSS, ROC and Kappa were used in combination to evaluate the performance of the models, with TSS serving as the selection metric for ensemble modelling.

### 2.6 Ensemble Modelling

**FIGURE 5:**
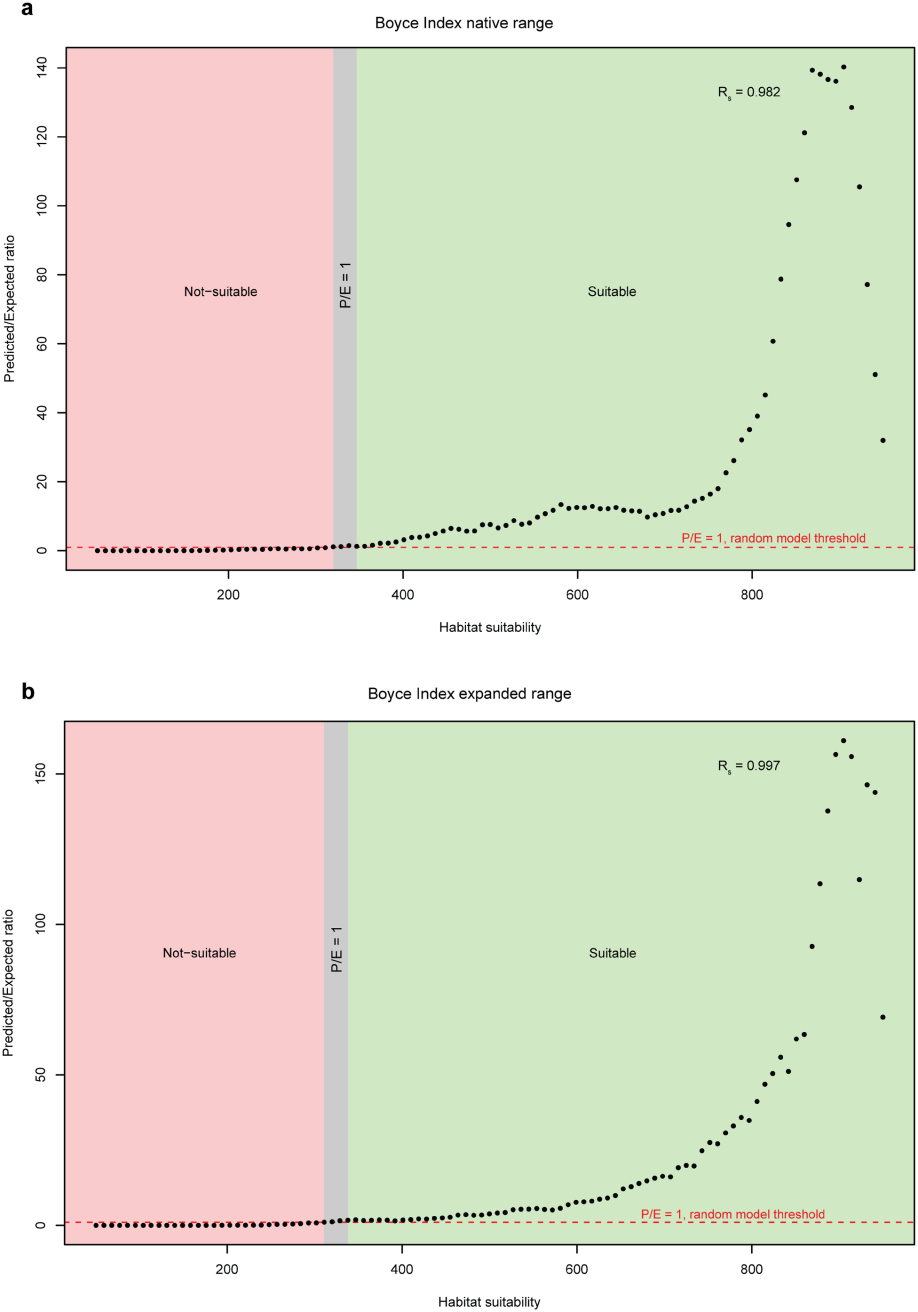
Continuous Boyce Index (CBI) of potential niches occupied by *Toxomerus floralis* , based on the Spearman Rank correlation coefficient (*R*_s_) of the predicted to expected ratio of species occurrence compared to habitat suitability for both (a) native and (b) expanded ranges.

The ‘BIOMOD_EnsembleModeling’ function of the ‘biomod2’ package was used to generate ensemble models. The ensemble models were assembled by combining all the previously generated models into one. Six modelling techniques or algorithms were used to create the ensemble models: Mean of probabilities over the selected models (EMmean), Median of probabilities over the selected models (EMmedian), Coefficient of variation of probabilities over the selected models (EMcv), Confidence interval around the mean of probabilities of the selected models (EMci), committee averaging (EMca) and weighted probability mean (EMwmean). For EMwmean, its EMwmean.decay parameter is set to proportional, meaning the weights are assigned proportionally to their evaluation scores, resulting in a fairer discrimination (Thuiller et al. 2024). The TSS of all the traditional single models was evaluated, and any with a TSS score lower than the 0.7 or 70% threshold were excluded from the ensemble. The TSS threshold 0.7 acts as a quality filter, ensuring that only robust models with good performance are retained. The remaining models are then combined and the six ensemble models run, using the same evaluation metrics as for the traditional modelling (TSS, ROC and Kappa) and three variable importance permutations. The EMci generates two ensemble models and a confidence interval alpha of 0.05 is used as significance level to estimate the upper and lower intervals. The ensemble model performance was further evaluated using the Continuous Boyce Index (CBI), based on the Spearman Rank correlation coefficient (*R*_s_) of the predicted to expected ratio of species occurrence compared to habitat suitability (Figure 5). The CBI was generated using the ‘ecospat.boyce’ function of the ‘ecospat’ package (Di Cola et al. 2017).

### 2.7 Global Climate Models (2021–2100)

At present there are 14 Global Climate Models (GCMs) that form part of WorldClim 2.1 datasets and that cover SSP 1–2.6 to SSP5–8.5. Of these, only 12 cover SSP2–4.5 and SSP5–8.5. These models all have varying levels of performance and associated biases, making it difficult to compare their performance when applying them to the Afrotropical region, or any potential regions where invasion might take place. M'mame and Ngongondo (2024) attempted to qualify the performance of 19 GCMs produced by several modelling groups (M'mame and Ngongondo 2024) by testing the models' performance in simulating climate extremes in Malawi. While the results did not provide a clear winner in terms of performance, the analyses did provide percentage bias in terms of predicting temperature and precipitation, variables that are important contributors in predicting the potential distribution of *T. floralis* .

These biases were, however, only measured for Malawi. To this end, a Multivariate Environmental Similarity Surfaces (MESS) analysis was performed on the applicable 12 Worldclim 2.1 GCMs to determine their extrapolation risk. This was done using the ‘terra’ and ‘predicts’ R packages to compare the known occurrences of *T. floralis* (both native and invaded), current bioclimatic variables (Bio2, Bio3, Bio8, Bio15, Bio18) and 12 GCMs (see Data S5).

EC‐Earth3‐Veg, MPI‐ESM1‐2‐HR and MRI‐ESM2‐0 were chosen, together with shared socioeconomic pathways SSP2–4.5 (regarded as middle of the road, with medium challenges to mitigation and adaptation) and SSP5–8.5 (fossil‐fueled development, with high challenges to mitigation, low challenges to adaptation). These three GCMs performed best under both shared socioeconomic pathways and showed the smallest divergence between emission scenarios, which makes them the most robust choice. The five suitable bioclimatic variables identified during the test for co‐linearity were then extracted from each of the GCMs and converted into 24 raster stacks, each representing unique combinations of models (EC‐Earth3‐Veg, MPI‐ESM1‐2‐HR or MRI‐ESM2‐0, SSP2–4.5, SSP5–8.5) and year range (2021–2040, 2041–2060, 2061–2080, 2081–2100).

The generated ensemble models for the native and expanded ranges of *T. floralis* were projected using the three GCMs, two SSPs and four different future date ranges (2021–2040, 2041–2060, 2061–2080, 2081–2100) with the intention of generating potential forecasts of *T. floralis* invasion in the future, as well as determining the gain or loss in potential distribution. The ‘BIOMOD_RangeSize’ function of the ‘biomod2’ package was used to compare pixel differences between current native and expanded projections and future projections of the potential distribution of *T. floralis* .

### 2.8 Niche Conservatism

**FIGURE 6:**
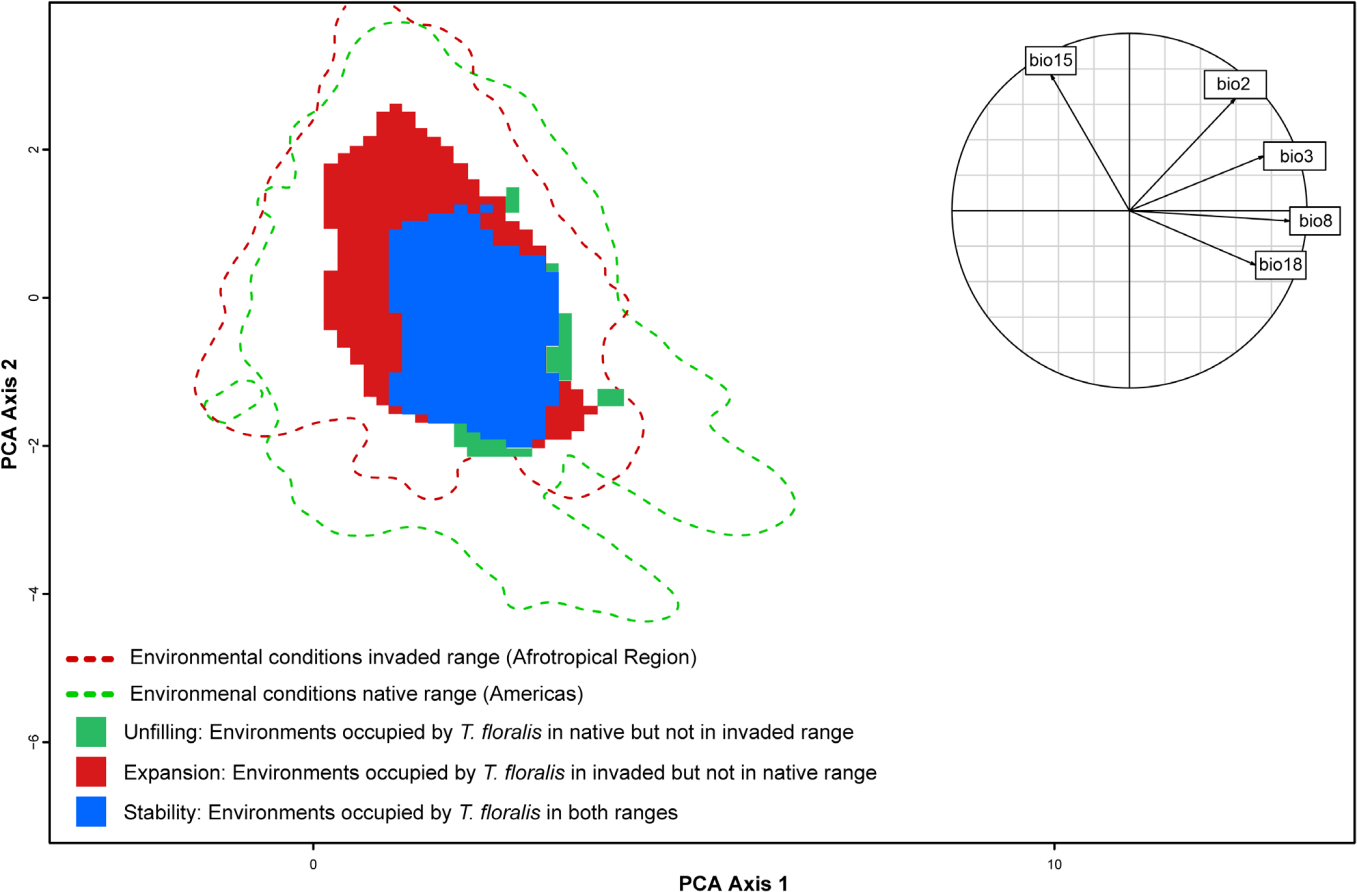
Climatic niche grids based on principal component analysis of bioclimatic variables (Bio2, Bio3, Bio8, Bio15 and Bio18) within the native and invaded ranges of *Toxomerus floralis* , allowing for a visual representation of the niche dynamics, that is, niche unfilling in green, expansion in red and stability in blue, within the environments occupied by *T. floralis* .

**FIGURE 7:**
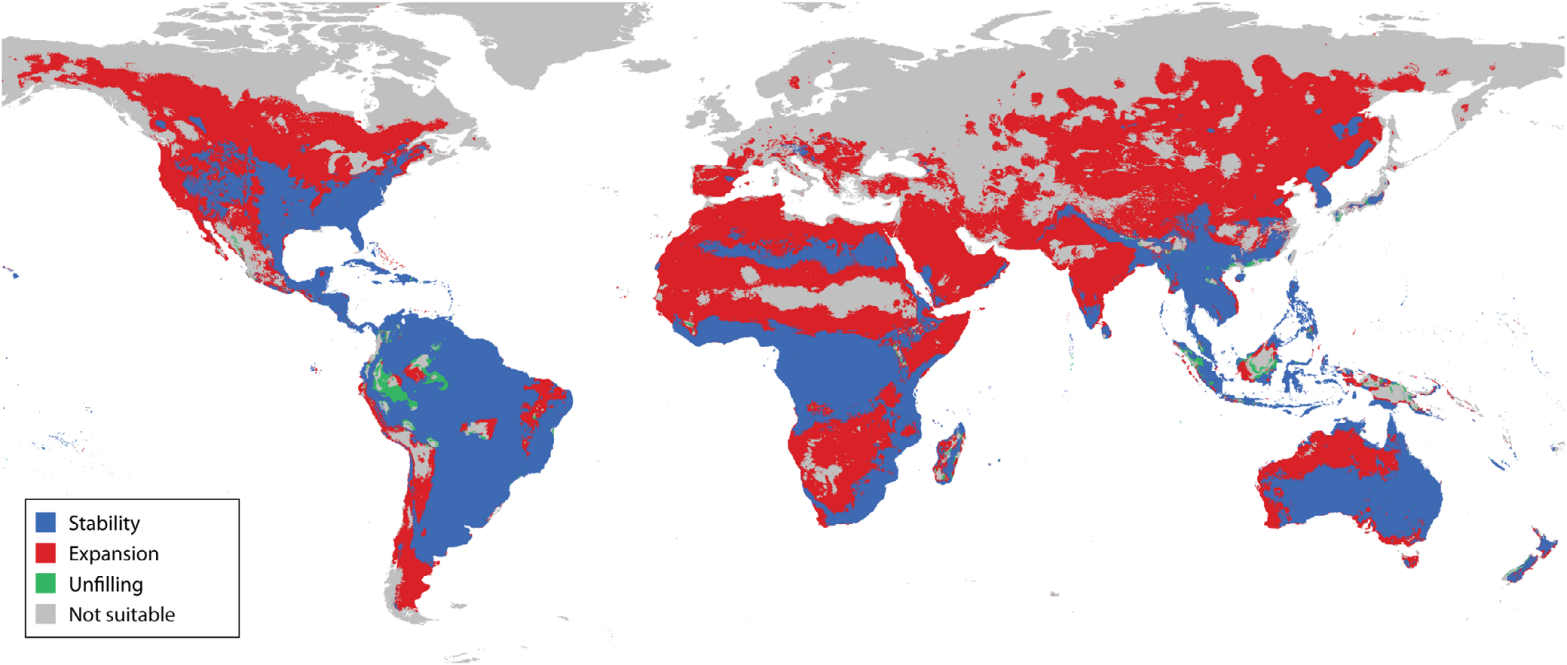
World map showing the niche dynamics for *Toxomerus floralis* between native and invaded ranges.

To analyse niche conservatism and whether a shift in niche utilisation has potentially occurred for *T. floralis* the ‘ecospat’ package was used. The species occurrences and bioclimatic variables (Bio2, Bio3, Bio8, Bio15 and Bio18) for the native range were compared to that of the invaded range. A PCA (using the ‘dudi.pca()’ function) was performed on the global environmental matrix as defined by the aforementioned selected bioclimatic variables to create a global score. The native and invaded range environmental and occurrence data are then projected into the PCA space and climatic niche grids were created using the ‘ecospat.grid.clim.dyn()’ function in ‘ecospat’, allowing the calculation of niche dynamics indices for examination (Figure 6). The climatic niche grids were then projected into geographic space to better visualise them (Figure 7). Finally a niche similarity test was performed to determine whether niche conservatism is present for *T. floralis* , determining the statistical significance of potential niche expansion, stability and whether the niche is unfilled, as well as the significance of Schoener's *D* and Hellinger's *I* values to measure niche overlap (Table S1). The test was performed with 2000 repetitions for increased randomisation.

### 2.9 Additional Information

Final distribution model projections and occurrence data of *T. floralis* , *C. rotundus and M. hirtus* were exported and mapped in qgis 3.28.6. All figure plates were made using adobe illustrator CS6 and photoshop cs5. All diagrams and model projections generated in rstudio 2023.12.1+ using packages as described in sections above were exported for inclusion in plates (see Data S15 for the R‐script and code used). The OD‐map protocol of Fitzpatrick et al. (2021) was followed and is provided in Data S16.

## 3 Results

### 3.1 Native Range Single Models

Out of 1332 model runs, 8.11% (*n* = 108) models passed the threshold and were retained for ensemble modelling, with a TSS score averaging 0.73. The 108 retained models consisted of 1 ANN, 15 CTA, 4 FDA, 28 GBM, 1 GLM, 13 MARS, 19 MAXNET, 20 RF and 7 XGBOOST (Data S6).

Of the models that did not meet the threshold, or were excluded, only 2.33% (*n* = 31) of models had a TSS score lower than 0.4; 79.65% (*n* = 1061) of models scored 0.4 or above (considered fair to moderate), but below what would be considered acceptable performance (0.7), and 9.90% (*n* = 132) were excluded as these are combined model results generated by the ‘biomod2’ package and as such TSS is not calculated for them.

### 3.2 Expanded Range Single Models

Out of the 1332 model runs, only 2.40% (*n* = 32) models were retained, with a TSS score averaging 0.7194 for use in ensemble modelling. The 32 retained models consisted of 1 CTA, 1 FDA, 12 GBM, 3 MARS, 1 MAXNET, 13 RF and 1 XGBOOST (Data S7).

Of the models that did not meet the threshold and were excluded, only 0.45% (*n* = 6) of models had a TSS score < 0.4, with 87.23% (*n* = 1162) of models scoring 0.4 or above (considered fair to moderate) but below what would be considered acceptable performance (0.7), and 9.90% (*n* = 132) were excluded as these are combined model results generated by the ‘biomod2’ package and, as such, TSS is not calculated for them.

### 3.3 Ensemble Modelling

**FIGURE 8:**
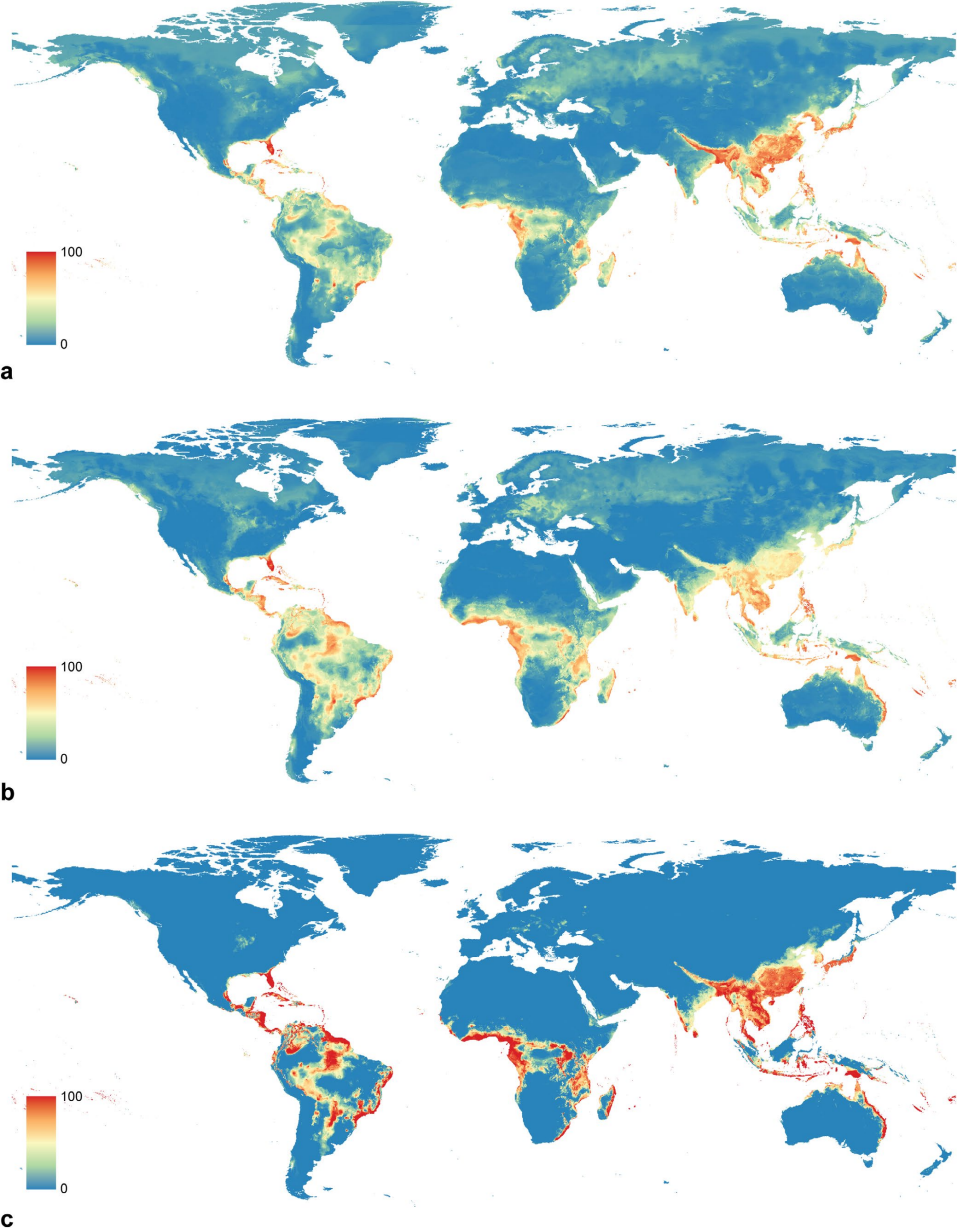
World projections of ensemble species distribution models for *Toxomerus floralis* based on (a) EMmedian modelling algorithm built on native range occurrence data, (b) EMmedian modelling algorithm built on expanded range (native + invaded) occurrence data, (c) EMca modelling algorithm built on expanded (native + invaded) occurrence data.

For the native range ensemble models, the median of probabilities (EMmedian) modelling technique performed the best (Data S8), with TSS = 0.824, ROC = 0.969, Kappa = 0.591. For the expanded range ensemble models, the committee averaging (EMca) modelling technique performed the best with TSS = 0.812, ROC = 0.971, Kappa = 0.641. However, EMmedian performed only marginally worse TSS = 0.805, ROC = 0.964, Kappa = 0.561 (Data S9) and when mapping the projections EMmedian was used instead for both ranges to allow for easier visual comparison. This is due to EMca working by committee averaging and is much more sensitive, resulting in a much more discriminant result, which could be considered more as a yes/no potential occurrence, compared to EMmedian which is the median of probabilities for the ensemble models and more conservative in its predictions. Figure 8 shows the world projections for *T. floralis* species distribution models and the differences between the native and expanded ranges and includes EMmedian and EMca for the expanded range to highlight the difficulty in comparing the two modelling techniques visually. All three projections show that there is a high probability of *T. floralis* spreading further, mostly throughout tropical to subtropical areas within the Afrotropical region. Additionally, the projections show that *T. floralis* can be expected to spread throughout the Eastern Palaearctic, Indomalayan and Australasian Regions in the near future.

The Continuous Boyce Index (CBI) was calculated for the native range ensemble model with a CBI Spearman correlation coefficient *R*_s_ of 0.982 indicating high model accuracy and predictive power (Figure 5a). Similarly, the expanded range ensemble model had a CBI Spearman correlation coefficient *R*_s_ of 0.997 indicating slightly higher model accuracy and predictive power (Figure 5b).

Utilising ensemble models allowed a combined approach to measure the variable importance of the five bioclimatic variables, giving insight into the potential effect these variables could have on the current and potential future distribution of *T. floralis* . Figures S4 and S5 show the resulting variable importance for all permutations, combined per modelling technique, for native and expanded ranges, respectively. For the native range Bio18 and Bio2 proved to be the two most important variables for all techniques, with the best performing technique, median of probabilities (EMmedian) having a mean variable importance of 42% (±0.9%) for Bio18 and 33% (±0.5%) for Bio2 (Data S10).

Similarly for the expanded range, Bio18 and Bio2 again proved to be the two most important variables for all techniques, being the only variables averaging above 20% variable importance for all techniques. The best performing technique for the expanded range, committee averaging (EMca), had a mean variable importance of 31% (±0.5%) for Bio18 and 62% (±0.9%) for Bio2 (Data S11).

This variable importance is also reflected in the response curves for both ranges (Figure S6) with Bio18 and Bio2 showing greatest influence to the species' potential distribution in response to changes in these climatic variables. Interestingly, the response curves for Bio2 (Figures S7B and S8B) show a rapid decline in occurrence probability for *T. floralis* when the mean of the difference of the monthly maximum and minimum temperature exceeds ca 8°C for the native range and ca 10.5°C for the expanded range. Similarly, the response curves for Bio18 (Figures S7A and S8A) show that the occurrence probability of *T. floralis* is highest where precipitation during the warmest 3 months of the year is around 600–650 mm for both ranges. The response curves for the native range plateau after this with a steady decline compared to that of the expanded range where it similarly plateaus but is stable at higher precipitation. These curves show that *T. floralis* would potentially do best in humid tropical or subtropical zones. This is also evident from the occurrence probability maps generated from the ensemble models for the native range and expanded ranges (Figure 8).

### 3.4 Host Plants

The distributions of the host plants *C. rotundus* and *M. hirtus* in the Afrotropical region (Figure S2) and Eastern Palaearctic, Indomalayan and Australasian Regions (Figure S3) are mapped to the native and expanded range potential distributions of *T. floralis* . There is a large degree of overlap between host records and projected potential distributions of *T. floralis* . However, the projections show that the host plants also occur in areas outside of the apparent climatic niches of *T. floralis* . At present, the importance of host plants with regards to the distribution of *T. floralis* has not been tested for.

### 3.5 Niche Conservatism

Figure 6 shows the PCA plot of the niche dynamics for *T. floralis* . It was created from a global environmental matrix using non‐correlated bioclimatic variables (Bio2, Bio3, Bio8, Bio15 and Bio18) as defined in the Section 2. PCA Axis 1 (48.97% of variance) is represented by a gradient with strong loadings from Bio8 (*r* = 0.904), Bio3 (*r* = 0.757) and Bio18 (*r* = 0.711), with moderate contribution by Bio2 (*r* = 0.597) and a negative contribution by Bio15 (*r* = −0.443), inferring a thermally stable, warm‐season moisture gradient (based on loadings by bioclimatic variables). Conversely, PCA Axis 2 (23.63% of variance) is represented by a gradient with strong loadings from Bio15 (*r* = 0.767) and Bio2 (*r* = 0.633) with minor contributions from Bio3 (*r* = 0.308), a negative contribution by Bio18 (*r* = −0.307) and a negligible contribution from Bio8 (*r* = −0.058), inferring a gradient that is characterised by precipitation seasonality and diurnal temperature range (see Data S12). Figure 6 has dashed lines representing the environmental conditions available for the invaded (red) and native (green) ranges, showing considerable overlap in conditions.

This aforementioned overlap is also reflected in the blue area of the plot, representing niche overlap, that is, available environmental conditions present in both ranges, supported by a niche dynamic index stability value of 0.764. Conversely, the red area represents niche expansion, that is, environmental conditions present in only the invaded but not native range, supported by a niche dynamic index expansion value of 0.236. Finally, the green area represents niche unfilling, that is, environmental conditions only available in the native range, supported by a niche dynamic index unfilling value of 0.012 (Table S1).

A niche similarity test which was performed to test for niche conservatism and statistical significance of the resulting indices (Table S1). The two indices, Schoener's *D* and Hellinger's *I*, returned values of 0.275 (*p* = 0.020) and 0.512 (*p* = 0.014), respectively. These indices are commonly used to measure niche conservatism with a value of 0 indicating no overlap and a value of 1 indicating complete overlap. The Schoener's *D* and Hellinger's *I* values are statistically significant (*p* < 0.05), and indicate that there is only minor to moderate niche overlap between the native and invaded ranges, indicating meaningful niche differentiation between native and invaded ranges for *T. floralis* .

Additionally, all the *p*‐values for the niche dynamic indices of expansion (*p* = 0.048), stability (*p* = 0.048) and unfilling (*p* = 0.025) are statistically significant. This indicates that *T. floralis* is able to expand into novel environmental conditions in the invaded range (niche expansion ≈24%), while still maintaining the majority of its native niche (≈76%). Less than ~1% of the native niche remains unfilled/is not utilised.

### 3.6 Global Climate Models (2021–2100)

When projecting current ensemble models to global climate maps, both the native and expanded range models were used as the apparent niche expansion in the Afrotropical region (see above section) necessitates also including expanded range data, as the species is clearly not limited to native range environmental conditions only (i.e., ~24% niche expansion) and the entire range of potential niches or differing climatic conditions it occupies need to be taken into consideration when projecting the models to future potential climates.

A total of 48 future projections were done, 24 (eight EC Earth3‐Veg, eight MRI‐ESM2‐0 and eight MPI‐ESM1‐2) each consisting of both SSP2‐4.5 and SSP5‐8.5 socioeconomic pathways and four date ranges: 2021–2040, 2041–2060, 2061–2080 and 2081–2100. These projections were made on the current native and expanded ensemble models, with the resulting range changes for each computed by comparing the resulting projections to the current native and expanded range projections for *T. floralis*. The 48 range change map figures are available in Figure S9, with Data S13 providing a summary of the resulting gain or loss of potential distribution for *T. floralis* . Figure S10 visualises the percentage gain and loss of potential distribution for the four future periods from 2021 to 2100 for each model and socioeconomic pathway based on the native range and expanded ranges. Figure S10 shows that *T. floralis* will potentially expand its range due to climate with no apparent contractions (see Data S13 for values).

## 4 Discussion

The Nearctic hover fly *Toxomerus floralis* was first detected in the Afrotropical region in 2013, in Sincoa, Cameroon (Jordaens et al. 2015), however, the initial point of entry into the Region remains unknown. Since first discovery, the species has been detected across much of the Afrotropical region, with first records from Kenya in 2016, South Africa in 2018 and Oman in 2020. By 2021, *T. floralis* was also reported from the island archipelagos of the Comoros and São Tomé. As of the end of July 2025, we have compiled a total of 181 occurrence records from over 25 Afrotropical countries (see Data S14 for our own non‐GBIF records). This would indicate a rapid expansion from its first detection based on current evidence. To date, *T. floralis* has not been recorded from Namibia, Angola or Madagascar, but this may partly reflect limited sampling effort and general underrepresentation of hover fly surveys in these regions.

**FIGURE 9:**
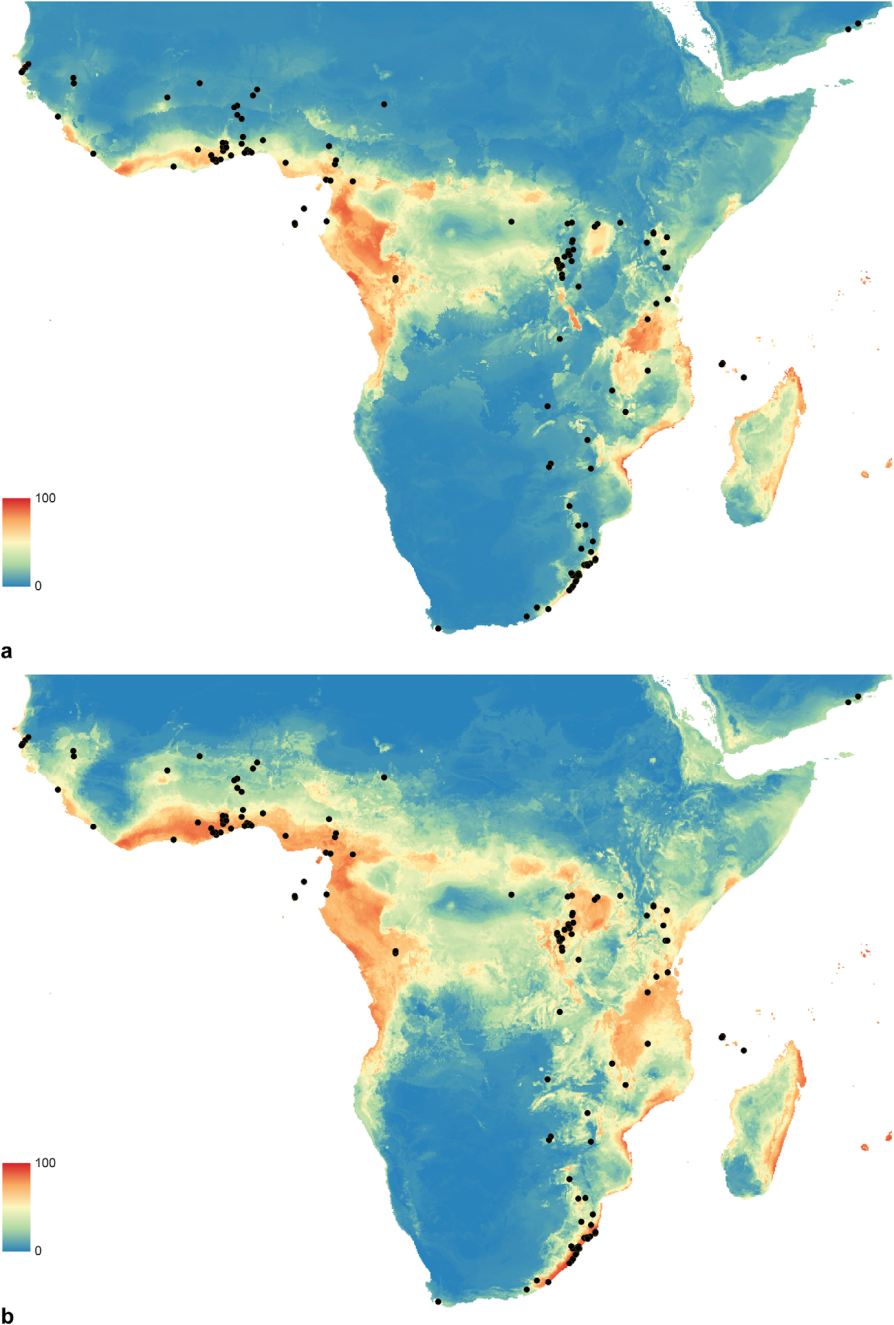
Afrotropical projection of (a) native range and (b) expanded range ensemble species distribution model of *Toxomerus floralis* based on the EMmedian modelling algorithm. Black circles indicated known occurrence data for *T. floralis* in the Afrotropics.

Ecological niche modelling, incorporating both native and climatic niche projections (see Figure 9), suggests that *T. floralis* has a high likelihood of occurrence in western Angola. In contrast, Namibia and Botswana are predicted to be largely climatically unsuitable, likely due to their semi‐arid conditions and cooler winter temperatures, factors that contrast with the apparent importance of rainfall and diurnal temperature range for the species. However, one post‐analysis record, an observation from Gaborone, Botswana (19 April 2025; iNaturalist observation 271895122), indicates that *T. floralis* is capable of establishing in at least some areas deemed unsuitable by the current models. It also highlights the need for more sampling in Namibia and Botswana to better understand the conditions in which *T. floralis* is capable of establishing.

The maximum West–East distribution range (between Senegal and Oman) is approximately 7600 km, while the North–South range (western Senegal to eastern Kenya and southern South Africa) exceeds 10,000 km. The broad geographical spread of *T. floralis* , combined with its presence across diverse habitats (from mesic and xeric savannas to Afromontane environments), high population densities and confirmed breeding, as evidenced by the presence of eggs, larvae and pupae at several locations, strongly suggests that the species is well‐established in the Afrotropical region. The aforementioned collectively satisfy recognised criteria for successful establishment of alien species in an invaded range, as laid out by the frameworks of Blackburn et al. (2011), but also Richardson et al. (2000).

Ensemble modelling and niche shift analyses further indicate that *T. floralis* is not only capable of colonising regions with bioclimatic conditions similar to its native range, but also of exploiting novel environmental conditions. Its current and projected distribution closely aligns with the range of its larval host plants, on whose pollen the larvae feed. Interestingly, the distribution of these host plants extends beyond the apparent climatic envelope of *T. floralis* . While this pattern is consistent with the hypothesis that the species' spread may be more constrained by its own physiological tolerances than by the distribution of its host plants, we caution that this inference is based on visual comparison only and was not formally tested for within the current modelling framework. Alternatively, these areas may be under‐sampled or not yet colonised due to limited dispersal time since introduction.

The low pass rates (2.33%; 8.11%) of native and expanded range models with regards to the true skill statistic (TSS) value (0.7) which was used as the primary metric to filter models before performing ensemble niche modelling should also be noted. While a cut‐off was set to ensure that only the most robust, well performing models were retained for analyses, respectively 79.65% and 87.23% of excluded native range and expanded range models still scored > 0.4, which is considered fair to moderate in terms of performance. The strict cut‐off was implemented as to ensure that ensemble performance and predictive accuracy would be as optimal as possible. The possibility that the TSS scores were penalised by spatial bias should not be discounted. The specimen records used in this study span a broad geographic gradient within the native range, and are far more clustered in the invaded range; such sampling asymmetry could make it harder for some algorithms to generalise and predict species' potential distribution. While we did implement spatial thinning, we could not necessarily negate other sampling biases and heterogeneity such as sampling effort, intensity and gaps which also affect traditional fieldwork sampling and citizen science records as contained on iNaturalist. Additionally, PCA Axis 1 (Figure 6) of the niche dynamics of *T. floralis* showed that 48.97% of the variance is characterised by mostly thermal stability. If large parts of the invaded range are homogenous in this regard it could also account for the lower individual model scores. The overall low percentage of individual models could also be a reflection of the ongoing nature of the invasion, as the species may not yet occupy all climatically suitable areas.

The niche dynamics analysis indicates that invasion by *T. floralis* into the Afrotropics is characterised primarily by niche conservatism; it is, however, also accompanied by a substantial degree of niche expansion into novel environmental conditions. A stability value of 0.764 (~76%) indicates that much of the native niche is retained in the invaded range, while the low, near zero unfilling value (0.012) shows that the majority of native range conditions remain occupied. Additionally, the analysis gives an expansion value of 0.236, suggesting ~24% of environmental conditions utilised in the invaded range are not present in the native range. This observed pattern is broadly consistent with Liu et al. (2020), who found that many invasive species largely conserve their climatic niches. However, the statistically significant similarity indices (Schoener's *D* = 0.275, *p* = 0.020; Hellinger's *I* = 0.512, *p* = 0.014) indicate only minor to moderate niche overlap, reflecting differences in the distribution and relative occupancy of environmental conditions between the native and invaded ranges rather than a total niche shift. Together, these results suggest that while *T. floralis* largely retains its native niche, it has also expanded into additional climatic conditions. Given the high stability (0.764), it is possible that this apparent expansion does not represent a true niche shift but rather an apparent niche shift, where a species appears to be using a different set of environmental conditions in a new area, but is actually just occupying a different part of its existing, stable fundamental niche (Guisan et al. 2014). However, at present we can only hypothesise, as not enough is known about all of *T. floralis* ' abiotic and biotic requirements within its environment. The apparent niche expansion could also possibly be due to sampling bias in both ranges. While effort was made to lessen the inherent bias through spatial thinning, the same issues as for low TSS scores crop up, with asymmetrical sampling and sampling effort also potentially affecting the niche expansion score due to under‐sampling and oversampling occurring in certain types of climates.

Both host plants have wide distributions. *Cyperus rotundus* is native to the Afrotropical region and introduced to the Americas, and *M. hirtus* is native to the Neotropics and introduced to the Afrotropical region. This bidirectional plant exchange possibly facilitated the intercontinental establishment of *T. floralis* . We hypothesise that *M. hirtus* was the original host in the native range, with a subsequent host shift to *C. rotundus* following introduction to the Afrotropical region. Notably, this host shift spans unrelated plant families (Rubiaceae and Cyperaceae), which is the only known event in hover flies with a pollinivorous larval feeding ecology. However, the lack of larval feeding data from the native range makes it unclear whether the host shift occurred pre‐ or post‐introduction. In the Afrotropical region, larval feeding has only been confirmed in Benin, leaving the extent of trophic plasticity across the introduced range unresolved. If larvae are indeed able to utilise both hosts across their entire range, this could further enhance the species' invasive potential. The possibility that *T. floralis* could utilise additional new hosts as it spreads should not be discounted, but at present no additional information is available. The only other known pollinivorous species of *Toxomerus* within the native range of *T. floralis* are *T*. *apegiensis* (Harbach, 1974), known from Suriname, French Guiana and Brazil and *T. politus* (Say, 1823), which is widespread throughout the Americas. *Toxomerus apegiensis* feeds on the pollen of the bambusoid grass *Olyra obliquifolia* Steud. and *T. politus* feeds on maize, *Zea mays* L., pollen (Reemer and Rotheray 2009), both Poaceae and unrelated to hosts of *T. floralis* . As such, there is no evidence to suggest that pollinivorous species in the native range of *T. floralis* could potentially limit its distribution or that the absence of other pollinivorous species in the invaded range is allowing for an expanded climatic niche.

The citizen science platform iNaturalist has played a pivotal role in tracking the expansion of *T. floralis* . Users upload geotagged photographs for species identification, which are subsequently verified by a global community (e.g., Unger et al. 2020). Approximately 60 of the 181 distribution records (i.e., ~33%) used in this study were sourced from iNaturalist (accessed on 31 July 2025). The earliest iNaturalist record of *T. floralis* is from 24 September 2015 in Kumasi, Ghana. Between January and July 2025, 35 additional Afrotropical observations, including the first from Botswana, were added to the platform. Intriguingly, recent records may also suggest a southward range expansion within its native Neotropical range, with all nine records from Brazil postdating 2021, and two new records from Ecuador in early 2023. An alternative explanation would be increased activity on iNaturalist or an increase in citizen science engagement from these countries, however this is similarly speculative.

The model projections indicate that more suitable habitat is likely to become available in the native range under future climatic scenarios. The case of *T. floralis* exemplifies how smartphone applications and citizen science can significantly enhance the monitoring of non‐native invasive species, as noted in recent studies (Howard et al. 2022; Dimson et al. 2023). The high model accuracy (CBI > 0.98), identified habitat suitability of the Indomalayan, Australasian and Eastern Palaearctic Regions and the rapid spread of *T. floralis* thus far suggests an imminent risk of invasion to the aforementioned regions. Imported plant materials should be monitored more closely, especially those associated with Cyperaceae and Rubiaceae. While the association between *T. floralis* and its current host plants is well‐established, further work integrating host plant distributions into predictive models would strengthen the basis for recommendation with regards to hosts or even other potential Cyperaceae and Rubiaceae hosts. However, given the demonstrated host shift from one plant family to another and the apparent ~24% niche expansion observed in the invaded range, *T. floralis* appears to occupy novel climatic conditions beyond those characterised or known in its native range, which could suggest a degree of ecological flexibility that warrants further investigation into its physiological tolerances and invasive potential. It would therefore be important that any attempted management or monitoring practices take into account the known and predicted distributions of *T. floralis* or risk underestimating its potential spread. With its shift to *C. rotundus* , its potential impact on native plant‐pollinator networks can also expand as it spreads with its hosts. While hoverflies are typically seen as beneficial pollinators, a highly successful (at least in terms of sheer rapid spread) pollinivorous generalist such as *T. floralis* could disrupt native pollination networks. The adults of *T. floralis* may compete with other pollinivorous insects for resources, and while unconfirmed, they may also act as inadvertent pollinators of their weed hosts. This could possibly increase the reproductive potential of its weed hosts, thereby further degrading native habitats where the weeds have invaded. Judging by the current and predicted future spread of the species, in conjunction with future climate projections, and the observed resilience and wide spread of *T. floralis* , long term studies on the interactions of the species with native syrphids and other insects will be needed to quantify its true impact.

## Author Contributions

**Burgert Muller:** conceptualization (lead), data curation (equal), formal analysis (lead), investigation (equal), methodology (equal), visualization (lead), writing – original draft (lead), writing – review and editing (equal). **John Midgley:** conceptualization (lead), data curation (equal), formal analysis (equal), investigation (equal), methodology (equal), visualization (equal), writing – original draft (equal), writing – review and editing (equal). **Georg Goergen:** data curation (equal), investigation (supporting), writing – review and editing (equal). **Ali Al Jahdhami:** data curation (equal), investigation (supporting), writing – review and editing (equal). **Michelson Azo'o Ela:** data curation (equal), investigation (supporting), writing – review and editing (equal). **Terence Bellingan:** data curation (equal), investigation (supporting), writing – review and editing (equal). **Simon Cavaillès:** data curation (equal), investigation (supporting), writing – review and editing (equal). **Robert Copeland:** data curation (equal), investigation (supporting), writing – review and editing (equal). **Marc De Meyer:** data curation (equal), investigation (supporting), writing – review and editing (equal). **Martin Hauser:** data curation (equal), investigation (supporting), writing – review and editing (equal). **Allen Holmes:** data curation (equal), investigation (supporting), writing – review and editing (equal). **Ximo Mengual:** data curation (equal), investigation (supporting), writing – review and editing (equal). **Gabriel Nève:** data curation (equal), investigation (supporting), writing – review and editing (equal). **Menno Reemer:** data curation (equal), investigation (supporting), writing – review and editing (equal). **Jeff Skevington:** conceptualization (equal), data curation (equal), investigation (supporting), writing – review and editing (equal). **Gunilla Ståhls:** data curation (equal), investigation (supporting), writing – review and editing (equal). **Eugène Sinzinkayo:** data curation (equal), investigation (supporting), writing – review and editing (equal). **John Smit:** data curation (equal), investigation (supporting), writing – review and editing (equal). **Axel Ssymank:** data curation (equal), investigation (supporting), writing – review and editing (equal). **Genevieve Theron:** data curation (equal), investigation (supporting), writing – review and editing (equal). **Kurt Jordaens:** conceptualization (lead), data curation (equal), formal analysis (supporting), funding acquisition (lead), investigation (equal), methodology (supporting), project administration (lead), visualization (supporting), writing – original draft (lead), writing – review and editing (equal).

## Funding

This project was financed through the JRS Biodiversity Foundation projects 60512 and 60868 PINDIP (Pollinator Information Network for two‐winged insects (Diptera); www.pindip.org), Belgian Federal Science Policy Office, BL/37/SA4, Belspo‐NRF joint network project DIPTATEACH (Diptera Museum collections as a source for Taxonomic research and Teaching activities) and DIPoDIP (Diversity of Pollinating Diptera in South African biodiversity hotspots) which is financed by the Directorate‐general Development Cooperation and Humanitarian Aid through the Framework agreement with RMCA. Field work in São Tomé and Príncipe was financed through Microland NGO.

## Conflicts of Interest

The authors declare no conflicts of interest.

## Supporting information


**Figure S1:** Occurrence records of *Toxomerus floralis* across the entire expanded range of the species. 235 native (yellow) and 128 invaded (purple).

**Figure S2:** Afrotropical projection of (a, c) native range and (b, d) expanded range ensemble species distribution model of *T. floralis* based on the EMmedian modelling algorithm versus (a, c) *Cyperus rotundus* and (b, d) *Mitracarpus hirtus* . Clear circles indicated occurrence data for host plants.

**Figure S3:** Eastern Palaearctic, Indomalayan and Australasian Regions projection of expanded range ensemble species distribution model of *T. floralis* based on the EMmedian modelling algorithm compared to the current known distribution of (a) *Cyperus rotundus* and (b) *Mitracarpus hirtus* . Clear circles indicated occurrence data for host plants.

**Figure S4:** Boxplot of variable importance for all ensemble modelling techniques combined for each selected bioclimatic variable (Bio2, Bio3, Bio8, Bio15 and Bio18) for native range.

**Figure S5:** Boxplot of variable importance for all ensemble modelling techniques combined for each selected bioclimatic variable (Bio2, Bio3, Bio8, Bio15 and Bio18) for expanded range.

**Figure S6:** Bioclimatic variable response curves for native and expanded ranges.

**Figure S7:** Response curves of Bio18 (Precipitation of Warmest Quarter) and Bio2 (Mean Diurnal Range) for each of the four algorithms (EMmean, EMmedian, EMca & Emwmean) used to generate native aensemble *T. floralis* distribution model.

**Figure S8:** Response curves of Bio18 (Precipitation of Warmest Quarter) and Bio2 (Mean Diurnal Range) for each of the four algorithms (EMmean, EMmedian, EMca & Emwmean) used to generate expanded ensemble *T. floralis* distribution model.

**Figure S9:** Range change maps of all 48 projected future climatic models compared by pixel difference with current native and expanded ensemble models for *T. floralis*. Allowing for a visual representation of loss or gain in potential distribution.

**Figure S10:** Graphic summary of the range change percentage gain or loss of predicted potential distributions for 48 future projections were done, 24 (8 Earth3‐Veg, 8 MRI‐ESM2‐0 and 8 MPI‐ESM1‐2) each consisting of both SSP2‐4.5 and SSP5‐8.5 socioeconomic pathways and 4 date ranges: 2021–2040, 2041–2060, 2061–2080 and 2081–2100.

**Table S1:** Niche dynamic indices and statistical significance tests for niche conservatism.

**Data S1:** Museums or private collections visited to look for specimens of *T. floralis* .

**Data S2:** WorldClim 2.1 Bioclimatic variable descriptions (https://www.worldclim.org).

**Data S3:** Variable Inflation Factors for all 19 bioclimatic variables (https://www.worldclim.org) within the native, invaded and expanded ranges of *Toxomerus floralis* , performed as supporting analysis of collinearity between the bioclimatic variables.

**Data S4:** Traditional modelling techniques, abbreviations and full names, based on information as provided by the ‘biomod2’ package (Guéguen et al. 2025).

**Data S5:** MESS analysis of 12 global climate models.

**Data S6:** Native range single model evaluation metrics: True skill statistic (TSS), receiver operating characteristic (ROC) and Cohen's Kappa coefficient (Kappa) to measure single model performance to determine potential occurrence of *Toxomerus floralis* .

**Data S7:** Expanded range single model evaluation metrics: True skill statistic (TSS), receiver operating characteristic (ROC) and Cohen's Kappa coefficient (Kappa) to measure single model performance to determine potential occurrence of *Toxomerus floralis* .

**Data S8:** Native range ensemble model evaluation metrics: True skill statistic (TSS), receiver operating characteristic (ROC) and Cohen's Kappa coefficient (Kappa) to measure ensemble model performance of four modelling algorithms: EMmean, EMmedian, EMca and EMwmean. To predict the potential occurrence of *Toxomerus floralis*.

**Data S9:** Expanded range ensemble model evaluation metrics: True skill statistic (TSS), receiver operating characteristic (ROC) and Cohen's Kappa coefficient (Kappa) to measure ensemble model performance of four modelling algorithms: EMmean, EMmedian, EMca and EMwmean. To predict the potential occurrence of *Toxomerus floralis*.

**Data S10:** Variable importance and contributions of the five bioclimatic variables (Bio2, Bio3, Bio8, Bio15 and Bio18) used in the generation of ensemble niche models of the potential distribution of *T. floralis* based on data from the native geographic range.

**Data S11:** Variable importance and contributions of the five bioclimatic variables (Bio2, Bio3, Bio8, Bio15 and Bio18) used in the generation of ensemble niche models of the potential distribution of *T. floralis* based on data from the expanded geographic range.

**Data S12:** Determination of gradients of environmental variation across the entire climatic space using eigenvalues to determine loadings of bioclimatic variables on each PCA axis.

**Data S13:** Summary of range change between current and future projected potential distribution of *T. floralis* based on native and expanded distribution ranges.

**Data S14:** Geographic information on localities where we collected *Toxomerus floralis* (‘own data’) or observations taken from iNaturalist (‘iNaturalist’) with decimal degrees for latitudes and longitudes and the date of the observation for Afrotropical records. In the case multiple observations were made on the same locality but on various dates, we only considered the earliest observation.

**Data S15:** R‐script used in generating predictive models, projections and figures.

**Data S16:** OD‐map Protocol for niche modelling of *T. floralis*.

## Data Availability

All data supporting the findings of this study are available within the paper and its Supporting Information. All GBIF data utilised is listed in Section 2 of the manuscript and can be accessed at: http://dx.doi.org/10.15468/dl.dqh46y, http://dx.doi.org/10.15468/dl.xqaym4, http://dx.doi.org/10.15468/dl.84a7g3.

## References

[ece373838-bib-0001] Aiello‐Lammens, M. E. , R. A. Boria , A. Radosavljevic , and B. Vilela . 2015. “spThin: An R Package for Spatial Thinning of Species Occurrence Records for Use in Ecological Niche Models.” Ecography 38, no. 5: 541–545. 10.1111/ecog.01132.

[ece373838-bib-0002] Allouche, O. , A. Tsoar , and R. Kadmon . 2006. “Assessing the Accuracy of Species Distribution Models: Prevalence, Kappa and the True Skill Statistic (TSS).” Journal of Applied Ecology 43, no. 6: 1223–1232. 10.1111/j.1365-2664.2006.01214.x.

[ece373838-bib-0003] Araújo, M. B. , R. P. Anderson , A. M. Barbosa , et al. 2019. “Standards for Distribution Models in Biodiversity Assessments.” Science Advances 5: eaat4858. 10.1126/sciadv.aat4858. 30746437 PMC6357756

[ece373838-bib-0004] Barve, N. , V. Barve , A. Jiménez‐Valverde , et al. 2011. “The Crucial Role of the Accessible Area in Ecological Niche Modeling and Species Distribution Modeling.” Ecological Modelling 222: 1810–1819. 10.1016/j.ecolmodel.2011.02.011.

[ece373838-bib-0005] Blackburn, T. M. , P. Pyšek , S. Bacher , et al. 2011. “A Proposed Unified Framework for Biological Invasions.” Trends in Ecology & Evolution 26: 333–339. 10.1016/j.tree.2011.03.023. 21601306

[ece373838-bib-0006] Borges, Z. M. , and M. S. Couri . 2009. “Revision of *Toxomerus* Macquart, 1855 (Diptera: Syrphidae) From Brazil With Synonymic Notes, Identification Key to the Species and Description of Three New Species.” Zootaxa 2179: 1–72. 10.11646/zootaxa.2179.1.1.

[ece373838-bib-0007] Broennimann, O. , M. C. Fitzpatrick , P. B. Pearman , et al. 2012. “Measuring Ecological Niche Overlap From Occurrence and Spatial Environmental Data.” Global Ecology and Biogeography 21, no. 4: 481–497. 10.1111/j.1466-8238.2011.00698.x.

[ece373838-bib-0008] Broennimann, O. , U. A. Treier , H. Müller‐Schärer , W. Thuiller , A. Peterson , and A. Guisan . 2007. “Evidence of Climatic Niche Shift During Biological Invasion.” Ecology Letters 10, no. 8: 701–709. 10.1111/j.1461-0248.2007.01060.x. 17594425

[ece373838-bib-0009] Chicco, D. , and G. Jurman . 2023. “The Matthews Correlation Coefficient (MCC) Should Replace the ROC AUC as the Standard Metric for Assessing Binary Classification.” Biodata Mining 16: 4. 10.1186/s13040-023-00322-4. 36800973 PMC9938573

[ece373838-bib-0010] Cook, D. F. , S. C. Voss , J. T. D. Finch , R. C. Rader , J. M. Cook , and C. J. Spurr . 2020. “The Role of Flies as Pollinators of Horticultural Crops: An Australian Case Study With Worldwide Relevance.” Insects 11, no. 6: 341. 10.3390/insects11060341. 32498457 PMC7349676

[ece373838-bib-0011] Di Cola, V. , O. Broennimann , B. Petitpierre , et al. 2017. “Ecospat: An R Package to Support Spatial Analyses and Modeling of Species Niches and Distributions.” Ecography 40, no. 6: 774–787. 10.1111/Fecog.02671.

[ece373838-bib-0012] Dimson, M. , L. B. Fortini , J. Morgan , W. Tingley , and T. W. Gillespie . 2023. “Citizen Science Can Complement Professional Invasive Plant Surveys and Improve Estimates of Suitable Habitat.” Diversity and Distributions 29: 1141–1156. 10.1111/ddi.13749.

[ece373838-bib-0013] Dray, S. , and A. Dufour . 2007. “The ade4 Package: Implementing the Duality Diagram for Ecologists.” Journal of Statistical Software 22, no. 4: 1–20. 10.18637/jss.v022.i04.

[ece373838-bib-0014] Early, R. , and D. F. Sax . 2014. “Niche Shift During Naturalization.” Global Ecology and Biogeography 23: 1356–1365. 10.1111/geb.12208.

[ece373838-bib-0015] Environmental Protection Authority . 2025. “Application for Approval to Import for Release or Release From Containment Any New Organism Under Section 34 of the Hazardous Substances and New Organisms Act 1996 (Form 1).” Environmental Protection Authority, Wellington, New Zealand. https://www.epa.govt.nz/assets/FileAPI/hsno‐ar/NOR00001/6b046d0b38/Application‐NOR00001.pdf.

[ece373838-bib-0016] Feng, X. , D. S. Park , C. Walker , A. Townsend Peterson , C. Merow , and M. Papeş . 2019. “A Checklist for Maximizing Reproducibility of Ecological Niche Models.” Nature Ecology & Evolution 3: 1382–1395. 10.1038/s41559-019-0972-5. 31548646

[ece373838-bib-0017] Feng, X. , A. T. Peterson , L. J. Aguirre‐López , J. R. Burger , X. Chen , and M. Papeş . 2024. “Rethinking Ecological Niches and Geographic Distributions in Face of Pervasive Human Influence in the Anthropocene.” Biological Reviews 99: 1481–1503. 10.1111/brv.13077. 38597328

[ece373838-bib-0018] Fick, S. E. , and R. J. Hijmans . 2017. “WorldClim 2: New 1km Spatial Resolution Climate Surfaces for Global Land Areas.” International Journal of Climatology 37, no. 12: 4302–4315. 10.1002/joc.5086.

[ece373838-bib-0019] Fitzpatrick, M. C. , S. Lachmuth , and N. T. Haydt . 2021. “The ODMAP Protocol: A New Tool for Standardized Reporting That Could Revolutionize Species Distribution Modeling.” Ecography 44: 1067–1070. 10.1111/ecog.05700.

[ece373838-bib-0020] GBIF (GBIF.org) . 2024a. “GBIF Occurrence Download.” 10.15468/dl.dqh46y.

[ece373838-bib-0021] GBIF (GBIF.org) . 2024b. “GBIF Occurrence Download.” 10.15468/dl.xqaym4.

[ece373838-bib-0022] GBIF (GBIF.org) . 2025. “GBIF Occurrence Download.” 10.15468/dl.84a7g3.

[ece373838-bib-0023] González‐Moreno, P. , J. M. Diez , D. M. Richardson , and M. Vilà . 2015. “Beyond Climate: Disturbance Niche Shifts in Invasive Species.” Global Ecology and Biogeography 24, no. 3: 360–370. 10.1111/geb.12271.

[ece373838-bib-0024] Guéguen, M. , H. Blancheteau , and W. Thuiller . 2025. “biomod2: Ensemble Platform for Species Distribution Modeling.” R package version 4.3‐2‐2. https://biomodhub.github.io/biomod2/.

[ece373838-bib-0025] Guisan, A. , B. Petitpierre , O. Broennimann , C. Daehler , and C. Kueffer . 2014. “Unifying Niche Shift Studies: Insights From Biological Invasions.” Trends in Ecology & Evolution 29, no. 5: 260–269. 10.1016/j.tree.2014.02.009. 24656621

[ece373838-bib-0026] Guisan, A. , W. Thuiller , and N. E. Zimmermann . 2017. “The Biomod2 Modeling Package Examples.” In Habitat Suitability and Distribution Models: With Applications in R. Ecology, Biodiversity and Conservation, 357–400. Cambridge University Press. 10.1017/9781139028271.

[ece373838-bib-0027] Gurevitch, J. , and D. K. Padilla . 2004. “Are Invasive Species a Major Cause of Extinctions?” Trends in Ecology & Evolution 19, no. 9: 470–474. 10.1016/j.tree.2004.07.005. 16701309

[ece373838-bib-0028] Howard, L. , C. B. van Rees , Z. Dahlquist , G. Luikart , and B. K. Hand . 2022. “A Review of Invasive Species Reporting Apps for Citizen Science and Opportunities for Innovation.” NeoBiota 71: 165–188. 10.3897/neobiota.71.79597.

[ece373838-bib-0029] Hulme, P. E. 2006. “Beyond Control: Wider Implications for the Management of Biological Invasions.” Journal of Applied Ecology 43: 835–847. 10.1111/j.1365-2664.2006.01227.x.

[ece373838-bib-0030] Jiménez‐Valverde, A. , A. T. Peterson , J. Soberón , J. M. Overton , P. Aragón , and J. M. Lobo . 2011. “Use of Niche Models in Invasive Species Risk Assessments.” Biological Invasions 13, no. 12: 2785–2797. 10.1007/s10530-011-9963-4.

[ece373838-bib-0031] Jordaens, K. , G. Goergen , A. H. Kirk‐Spriggs , A. Vokaer , T. Backeljau , and M. De Meyer . 2015. “A Second New World Hoverfly, *Toxomerus floralis* (Fabricius) (Diptera: Syrphidae), Recorded From the Old World, With Description of Larval Pollen‐Feeding Ecology.” Zootaxa 4044: 567–576. 10.11646/zootaxa.4044.4.6. 26624726

[ece373838-bib-0032] Keane, R. M. , and M. J. Crawley . 2002. “Exotic Plant Invasions and the Enemy Release Hypothesis.” Trends in Ecology & Evolution 17, no. 4: 164–170. 10.1016/S0169-5347(02)02499-0.

[ece373838-bib-0033] Kondo, T. , R. Rosero , and J. Gaviria . 2024. “ *Eristalinus taeniops* (Wiedemann, 1818) (Diptera: Syrphidae), an Exotic Flower Fly Rapidly Spreading in South America: A Review.” Revista Chilena de Entomología 50, no. 3: 589–599. 10.35249/rche.50.3.24.17.

[ece373838-bib-0034] Liu, C. , C. Wolter , W. Xian , and J. M. Jeschke . 2020. “Most Invasive Species Largely Conserve Their Climatic Niche.” Proceedings of the National Academy of Sciences of the United States of America 117, no. 38: 23643–23651. 10.1073/pnas.2004289117. 32883880 PMC7519298

[ece373838-bib-0035] Merow, C. , M. J. Smith , and J. A. Silander Jr. 2013. “A Practical Guide to MaxEnt for Modeling Species' Distributions: What It Does, and Why Inputs and Settings Matter.” Ecography 36: 1058–1069. 10.1111/j.1600-0587.2013.07872.x.

[ece373838-bib-0036] M'mame, B. , and C. Ngongondo . 2024. “Evaluation of CMIP6 Model Skills in Simulating Tropical Climate Extremes Over Malawi, Southern Africa.” Modeling Earth Systems and Environment 10: 1695–1709. 10.1007/s40808-023-01867-3.

[ece373838-bib-0037] Naimi, B. , N. Hamm , T. A. Groen , A. K. Skidmore , and A. G. Toxopeus . 2014. “Where Is Positional Uncertainty a Problem for Species Distribution Modelling.” Ecography 37: 191–203. 10.1111/j.1600-0587.2013.00205.x.

[ece373838-bib-0038] Oliveira, B. F. , G. C. Costa , and C. R. Fonseca . 2018. “Niche Dynamics of Two Cryptic Prosopis Invading South American Drylands.” Biological Invasions 20, no. 1: 181–194. 10.1007/s10530-017-1525-y.

[ece373838-bib-0039] Omezine, A. , and F. Harzallah‐Skhili . 2009. “Biological Behavior of *Cyperus rotundus* in Relation to Agro‐Ecological Conditions and Imposed Human Factors.” African Journal of Plant Science and Biotechnology 3: 63–69.

[ece373838-bib-0040] Ørsted, I. V. , and M. Ørsted . 2019. “Species Distribution Models of the Spotted Wing Drosophila (*Drosophila suzukii*, Diptera: Drosophilidae) in Its Native and Invasive Range Reveal an Ecological Niche Shift.” Journal of Applied Ecology 56: 423–435. 10.1111/1365-2664.13285.

[ece373838-bib-0041] Pearman, P. B. , A. Guisan , O. Broennimann , and C. F. Randin . 2008. “Niche Dynamics in Space and Time.” Trends in Ecology & Evolution 23, no. 3: 149–158. 10.1016/j.tree.2007.11.005. 18289716

[ece373838-bib-0042] Peterson, A. T. , and C. R. Robins . 2003. “Using Ecological‐Niche Modeling to Predict Barred Owl Invasions With Implications for Spotted Owl Conservation.” Conservation Biology 17, no. 4: 1161–1165. 10.1046/j.1523-1739.2003.02206.x.

[ece373838-bib-0043] R Core Team . 2017. R: A Language and Environment for Statistical Computing. R Foundation for Statistical Computing. https://www.R‐project.org/.

[ece373838-bib-0044] Ramage, T. , C. Sylvain , and X. Mengual . 2018. “Flower Flies (Diptera, Syrphidae) of French Polynesia, With the Description of Two New Species.” European Journal of Taxonomy 448: 1–37. 10.5852/ejt.2018.448.

[ece373838-bib-0045] Reemer, M. , and G. E. Rotheray . 2009. “Pollen Feeding Larvae in the Presumed Predatory Syrphine Genus *Toxomerus* Macquart (Diptera, Syrphidae).” Journal of Natural History 43, no. 15–16: 939–949. 10.1080/00222930802610576.

[ece373838-bib-0046] Richardson, D. M. , P. Pyšek , M. Rejmánek , M. G. Barbour , F. D. Panetta , and C. J. West . 2000. “Naturalization and Invasion of Alien Plants: Concepts and Definitions.” Diversity and Distributions 6, no. 2: 93–107. 10.1046/j.1472-4642.2000.00083.x.

[ece373838-bib-0047] Roy, H. E. , J. Peyton , D. C. Aldridge , et al. 2014. “Horizon Scanning for Invasive Alien Species With the Potential to Threaten Biodiversity in Great Britain.” Global Change Biology 20: 3859–3871. 10.1111/gcb.12603. 24839235 PMC4283593

[ece373838-bib-0048] Shorthouse, D. P. 2010. “SimpleMappr, an Online Tool to Produce Publication‐Quality Point Maps.” https://www.simplemappr.net.

[ece373838-bib-0049] Sillero, N. , and A. M. Barbosa . 2020. “Common Mistakes in Ecological Niche Models.” International Journal of Geographical Information Science 35: 213–226.

[ece373838-bib-0050] Skevington, J. H. , A. D. Young , M. M. Locke , and K. M. Moran . 2019. “New Syrphidae (Diptera) of North‐Eastern North America.” Biodiversity Data Journal 7: e36673. 10.3897/BDJ.7.e36673. 31543695 PMC6736894

[ece373838-bib-0051] Thomas, J. A. 2005. “Monitoring Change in the Abundance and Distribution of Insects Using Butterflies and Other Indicator Groups.” Philosophical Transactions of the Royal Society, B: Biological Sciences 360: 339–357. 10.1098/rstb.2004.1585. PMC1569450 15814349

[ece373838-bib-0052] Thompson, F. C. 2013. “Family Syrphidae.” In Systema Dipterorum, edited by F. C. Thompson and T. Pape Version 1.5. http://www.diptera.org.

[ece373838-bib-0053] Thompson, F. C. , and B. J. Thompson . 2006. “A New *Toxomerus* Species From Chile (Diptera: Syrphidae).” Studia Dipterologica 13: 317–331.

[ece373838-bib-0054] Thuiller, W. , D. Georges , M. Gueguén , et al. 2024. “biomod2: Ensemble Platform for Species Distribution Modeling.” R Package Version 4.2‐5‐2. https://biomodhub.github.io/biomod2/.

[ece373838-bib-0055] Thuiller, W. , D. M. Richardson , P. Pyšek , G. F. Midgley , G. O. Hughes , and M. Rouget . 2005. “Niche‐Based Modelling as a Tool for Predicting the Risk of Alien Plant Invasions at a Global Scale.” Global Change Biology 11, no. 12: 2234–2250. 10.1111/j.1365-2486.2005.001018.x. 34991288

[ece373838-bib-0056] Turner, R. M. , E. G. Brockerhoff , C. Bertelsmeier , et al. 2021. “Worldwide Border Interceptions Provide a Window Into Human‐Mediated Global Insect Movement.” Ecological Applications 31, no. 7: e02412. 10.1002/eap.2412. 34255404

[ece373838-bib-0057] Unger, S. , M. Rollins , A. Tietz , and H. Dumais . 2020. “iNaturalist as an Engaging Tool for Identifying Organisms in Outdoor Activities.” Journal of Biological Education 55: 537–547. 10.1080/00219266.2020.1739114.

[ece373838-bib-0058] Vilà, M. , J. L. Espinar , M. Hejda , et al. 2011. “Ecological Impacts of Invasive Alien Plants: A Meta‐Analysis of Their Effects on Species, Communities and Ecosystems.” Ecology Letters 14, no. 7: 702–708. 21592274 10.1111/j.1461-0248.2011.01628.x

[ece373838-bib-0059] Wiens, J. J. , and C. H. Graham . 2005. “Niche Conservatism: Integrating Evolution, Ecology, and Conservation Biology.” Annual Review of Ecology, Evolution, and Systematics 36: 519–539. 10.1146/annurev.ecolsys.36.102803.095431.

[ece373838-bib-0060] Yoon, S. , and W.‐H. Lee . 2023. “Application of True Skill Statistics as a Practical Method for Quantitatively Assessing CLIMEX Performance.” Ecological Indicators 146: 109830. 10.1016/j.ecolind.2022.109830.

