# Supplementary figures and images for: Invasion Patterns and Niche Dynamics of the Pollinivorous Florida Calligrapher, Toxomerus floralis (Diptera: Syrphidae) in the Afrotropical Region
Source: Ecol Evol. 2026 Jun 23;16(6):e73838. doi: 10.1002/ece3.73838 (PMC13288174; doi:10.1002/ece3.73838)

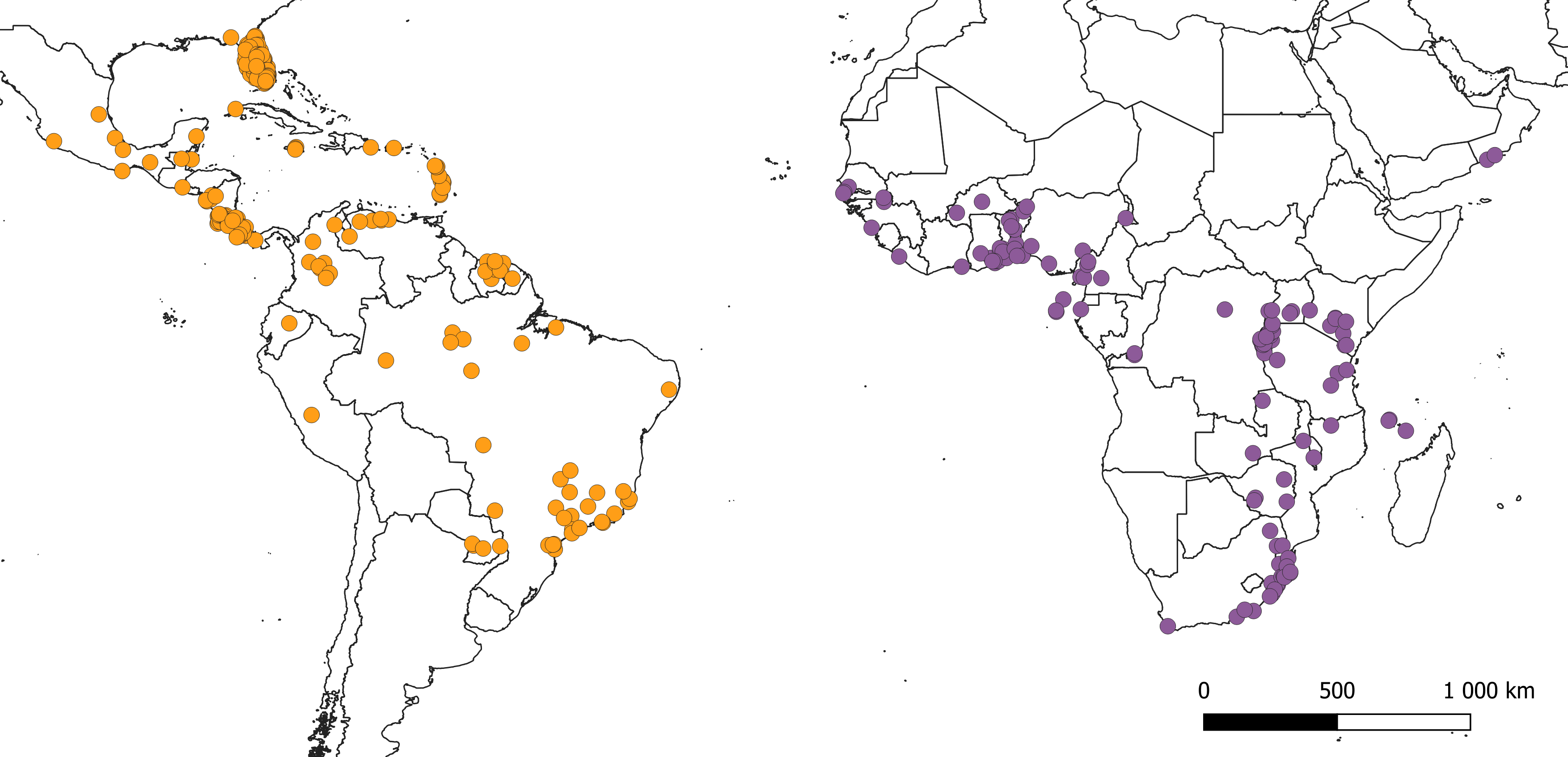

Supplement: Supplementary file 1 — Figure S1: Occurrence records of Toxomerus floralis across the entire expanded range of the species. 235 native (yellow) and 128 invaded (purple). [file ECE3-16-e73838-s005.tif]

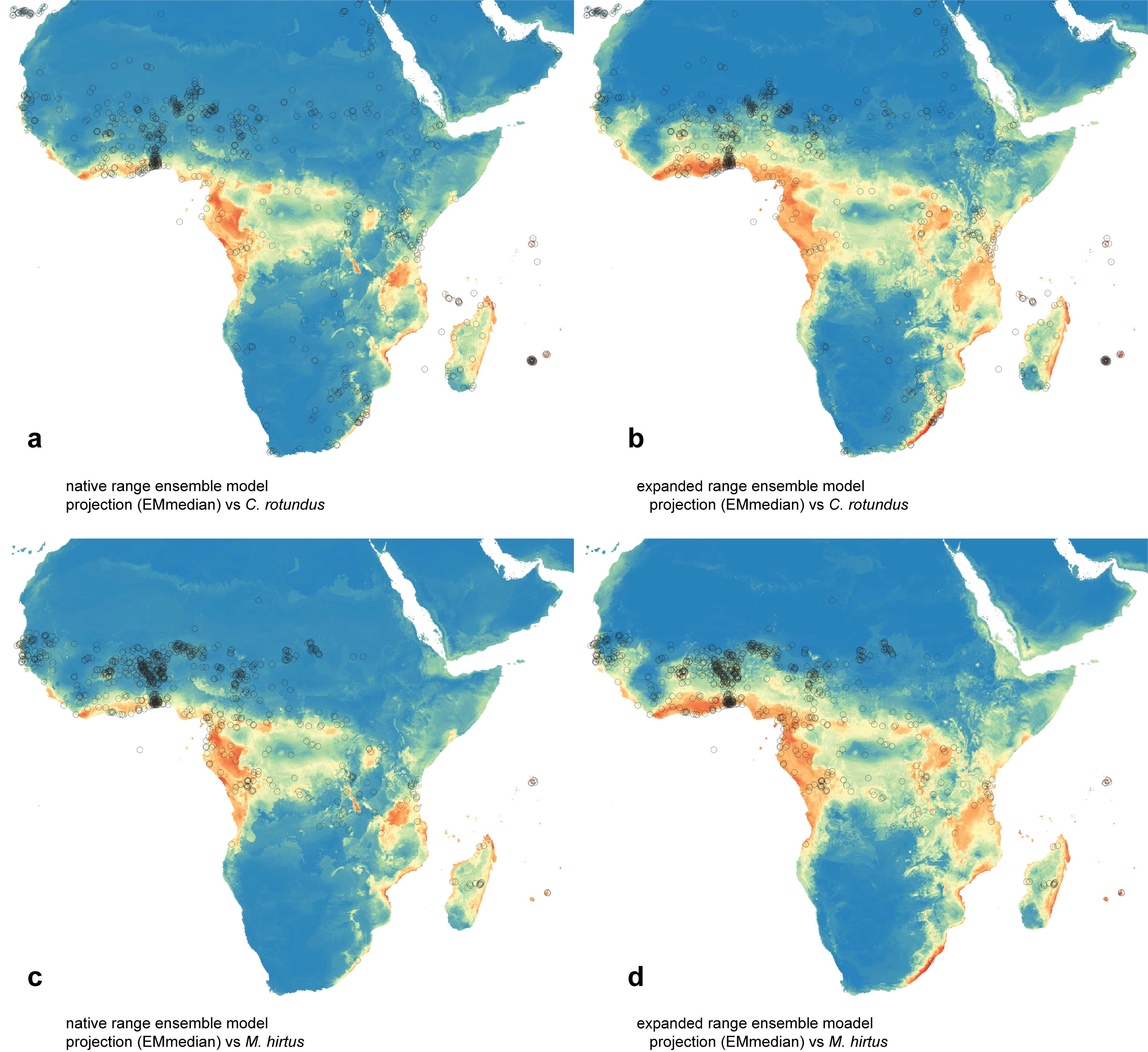

Supplement: Supplementary file 2 — Figure S2: Afrotropical projection of (a, c) native range and (b, d) expanded range ensemble species distribution model of T. floralis based on the EMmedian modelling algorithm versus (a, c) Cyperus rotundus and (b, d) Mitracarpus hirtus . Clear circles indicated occurrence data for host plants. [file ECE3-16-e73838-s003.tif]

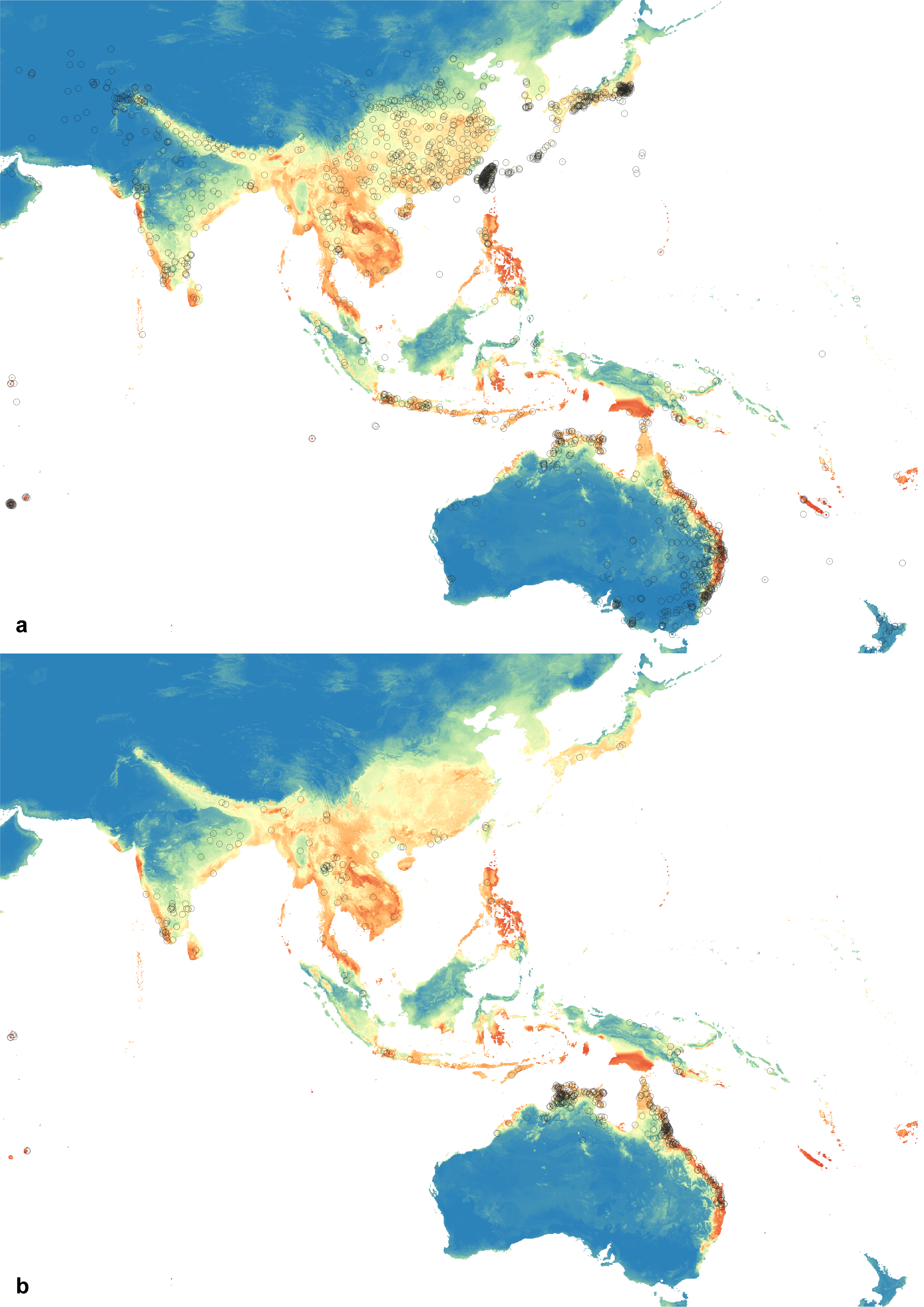

Supplement: Supplementary file 3 — Figure S3: Eastern Palaearctic, Indomalayan and Australasian Regions projection of expanded range ensemble species distribution model of T. floralis based on the EMmedian modelling algorithm compared to the current known distribution of (a) Cyperus rotundus and (b) Mitracarpus hirtus . Clear circles indicated occurrence data for host plants. [file ECE3-16-e73838-s018.tif]

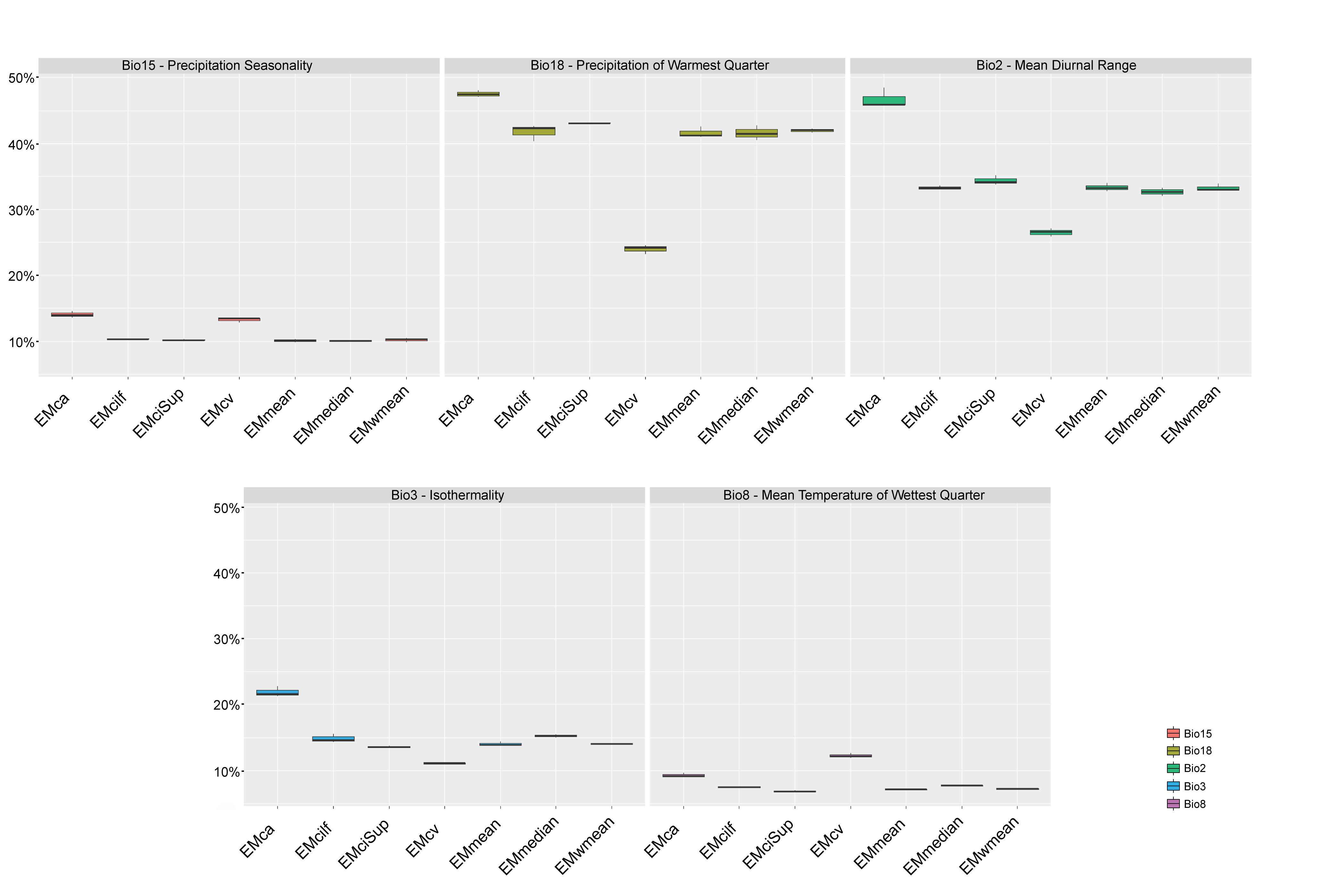

Supplement: Supplementary file 4 — Figure S4: Boxplot of variable importance for all ensemble modelling techniques combined for each selected bioclimatic variable (Bio2, Bio3, Bio8, Bio15 and Bio18) for native range. [file ECE3-16-e73838-s010.tif]

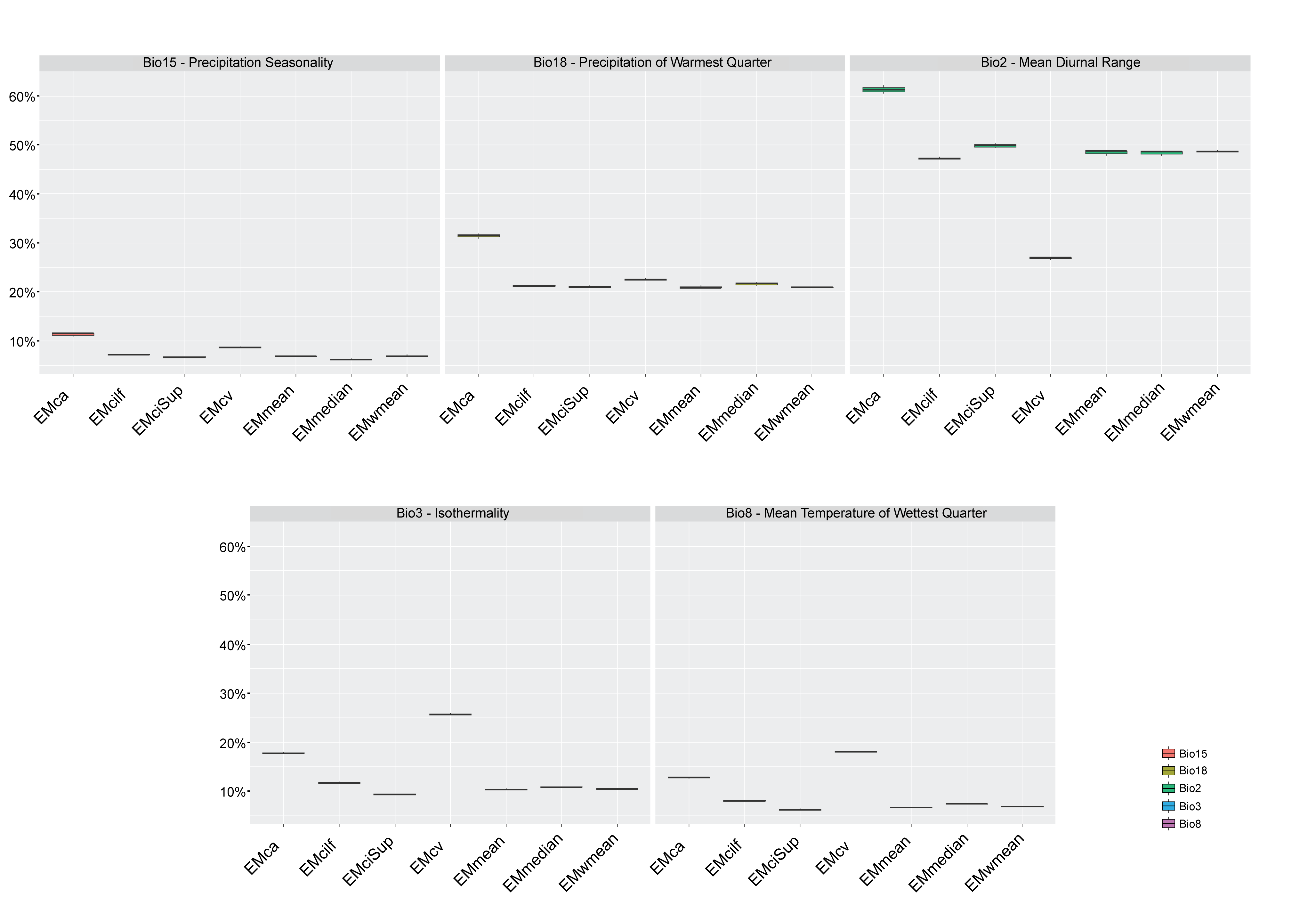

Supplement: Supplementary file 5 — Figure S5: Boxplot of variable importance for all ensemble modelling techniques combined for each selected bioclimatic variable (Bio2, Bio3, Bio8, Bio15 and Bio18) for expanded range. [file ECE3-16-e73838-s022.tif]

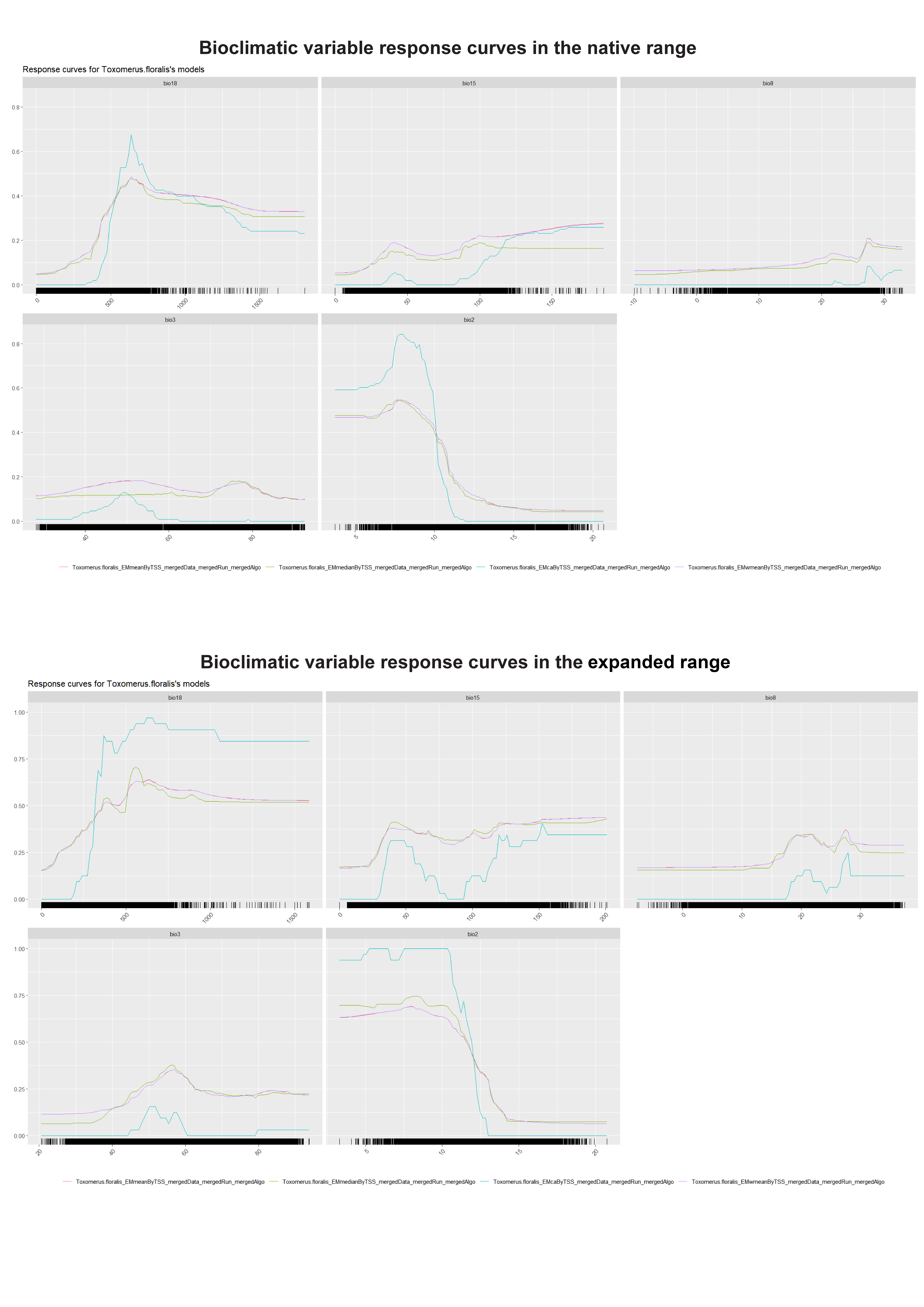

Supplement: Supplementary file 6 — Figure S6: Bioclimatic variable response curves for native and expanded ranges. [file ECE3-16-e73838-s002.tif]

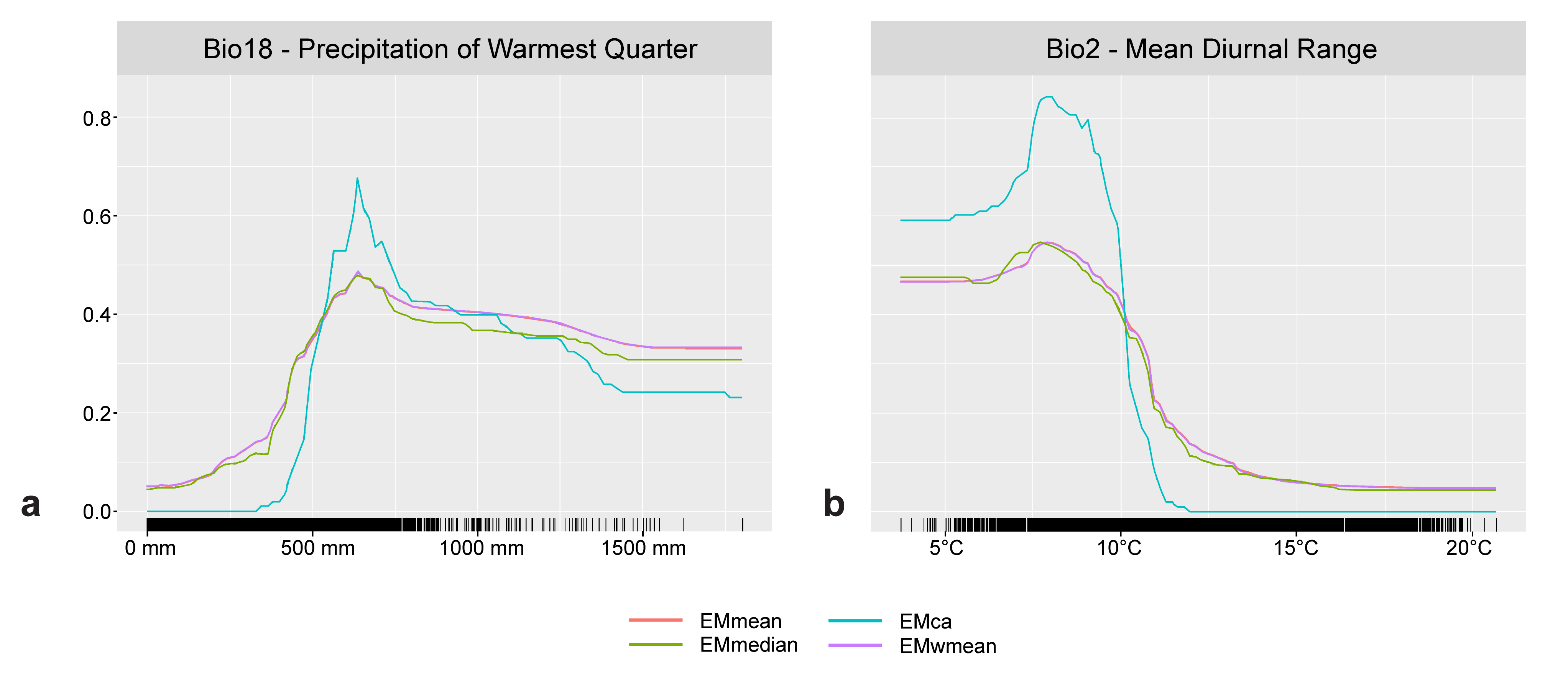

Supplement: Supplementary file 7 — Figure S7: Response curves of Bio18 (Precipitation of Warmest Quarter) and Bio2 (Mean Diurnal Range) for each of the four algorithms (EMmean, EMmedian, EMca & Emwmean) used to generate native aensemble T. floralis distribution model. [file ECE3-16-e73838-s009.tif]

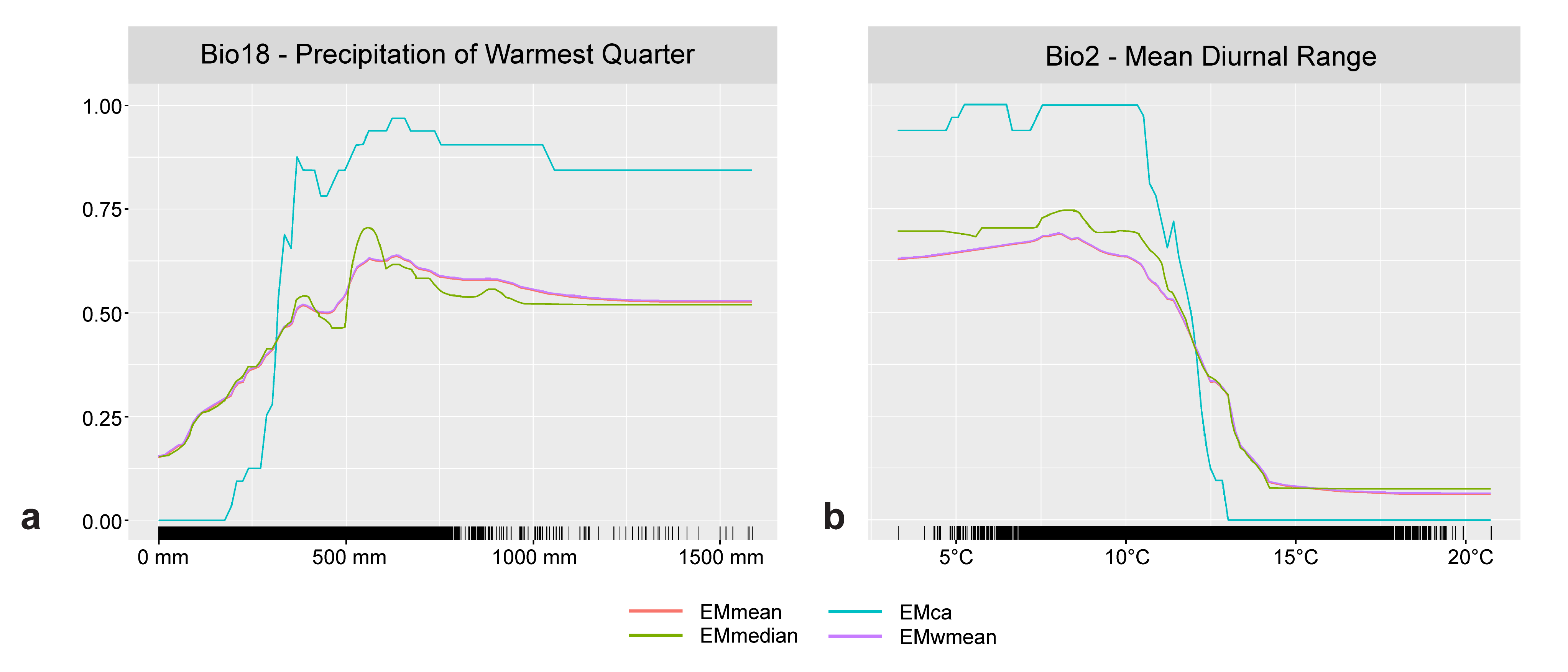

Supplement: Supplementary file 8 — Figure S8: Response curves of Bio18 (Precipitation of Warmest Quarter) and Bio2 (Mean Diurnal Range) for each of the four algorithms (EMmean, EMmedian, EMca & Emwmean) used to generate expanded ensemble T. floralis distribution model. [file ECE3-16-e73838-s013.tif]

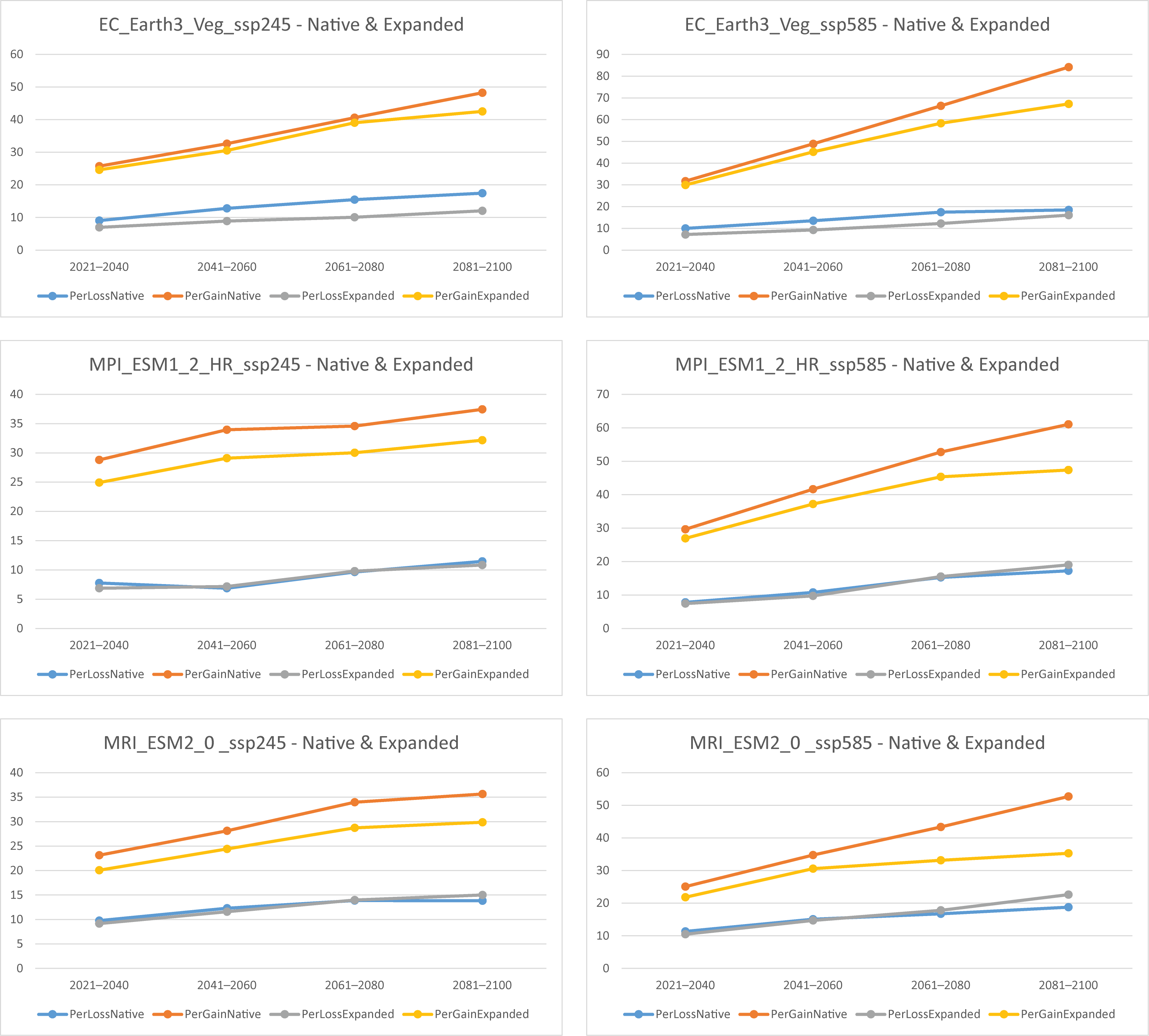

Supplement: Supplementary file 10 — Figure S10: Graphic summary of the range change percentage gain or loss of predicted potential distributions for 48 future projections were done, 24 (8 Earth3‐Veg, 8 MRI‐ESM2‐0 and 8 MPI‐ESM1‐2) each consisting of both SSP2‐4.5 and SSP5‐8.5 socioeconomic pathways and 4 date ranges: 2021–2040, 2041–2060, 2061–2080 and 2081–2100. [file ECE3-16-e73838-s026.tif]
