# Supplementary material for: Invasion Patterns and Niche Dynamics of the Pollinivorous Florida Calligrapher, Toxomerus floralis (Diptera: Syrphidae) in the Afrotropical Region
Source: Ecol Evol. 2026 Jun 23;16(6):e73838. doi: 10.1002/ece3.73838 (PMC13288174; doi:10.1002/ece3.73838)

Native Range Projections

EC-Earth3-Veg ssp245 (2021–2040) – Habitat Change

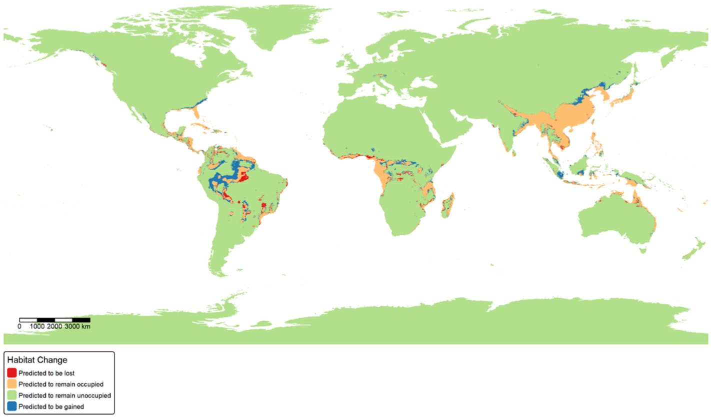

EC-Earth3-Veg ssp245 (2041–2060) – Habitat Change

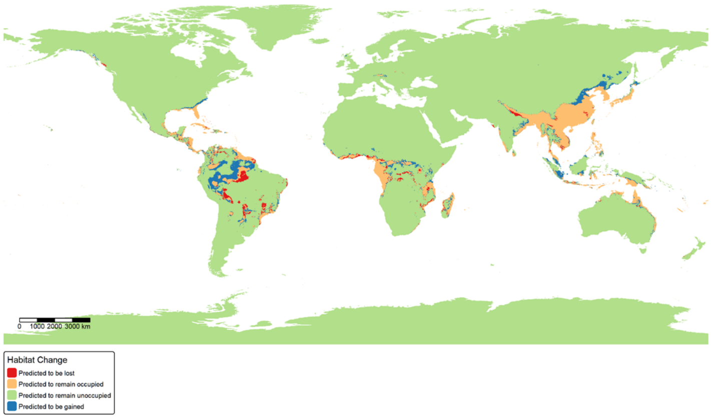

EC-Earth3-Veg ssp245 (2061–2080) – Habitat Change

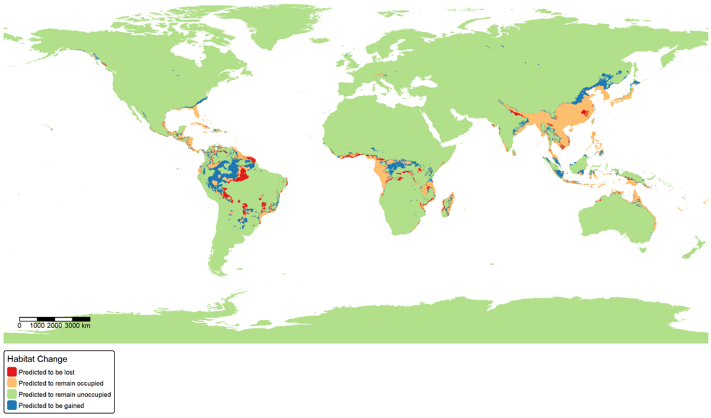

EC-Earth3-Veg ssp245 (2081–2100) – Habitat Change

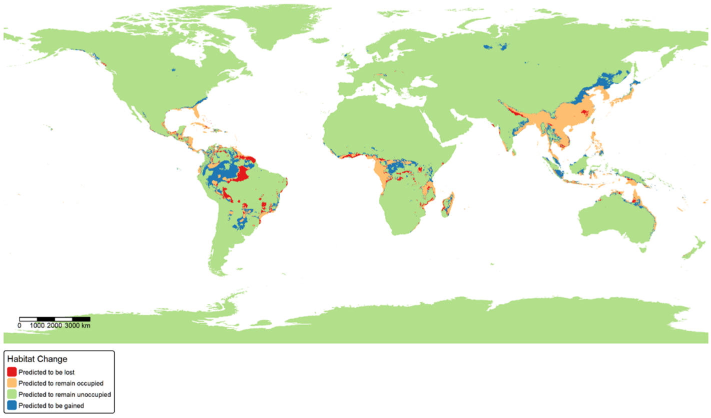

EC-Earth3-Veg ssp585 (2021–2040) – Habitat Change

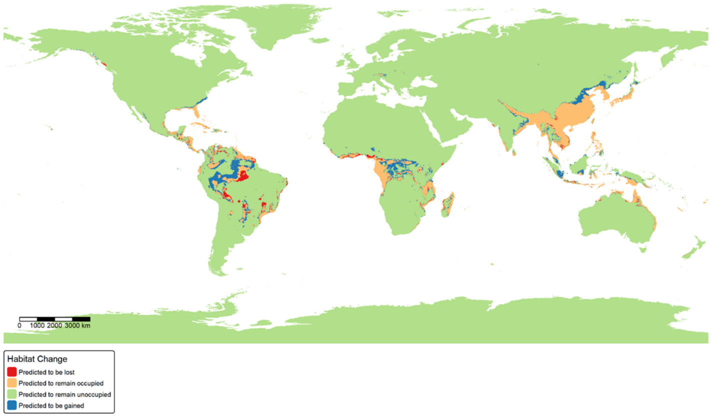

EC-Earth3-Veg ssp585 (2041–2060) – Habitat Change

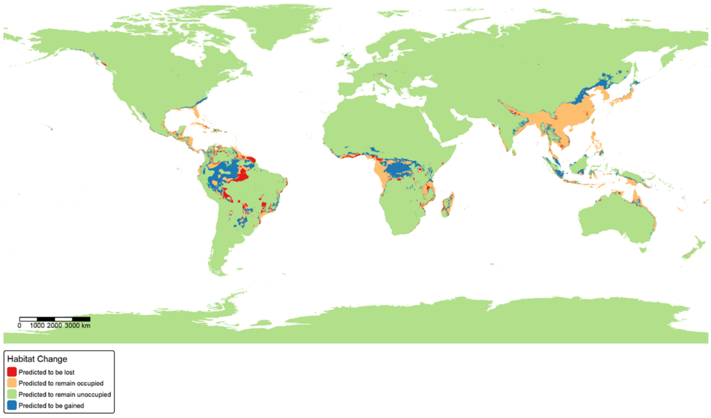

EC-Earth3-Veg ssp585 (2061–2080) – Habitat Change

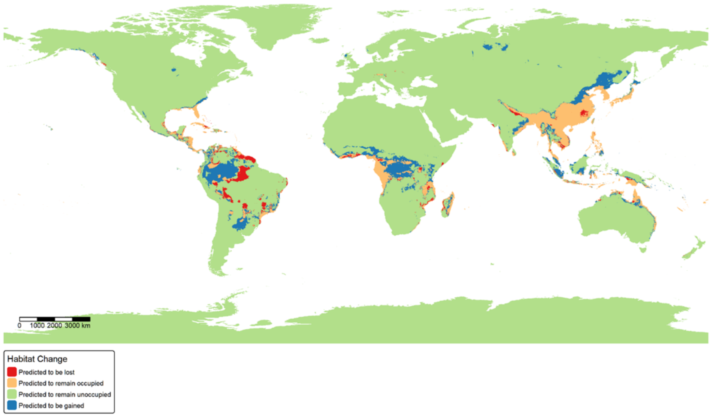

EC-Earth3-Veg ssp585 (2081–2100) – Habitat Change

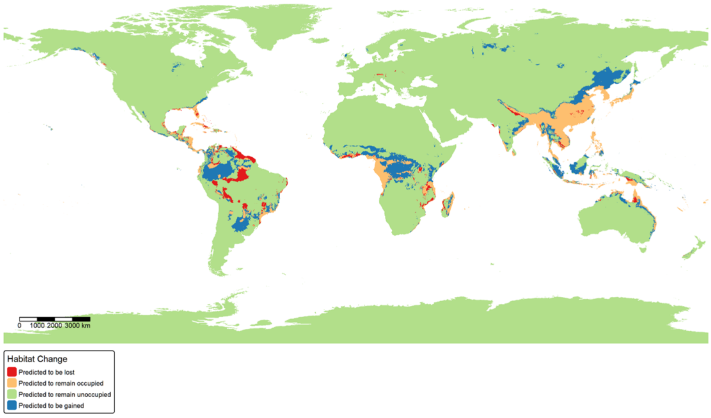

Native Range Projections

MPI-ESM1-2-HR ssp245 (2021–2040) – Habitat Change

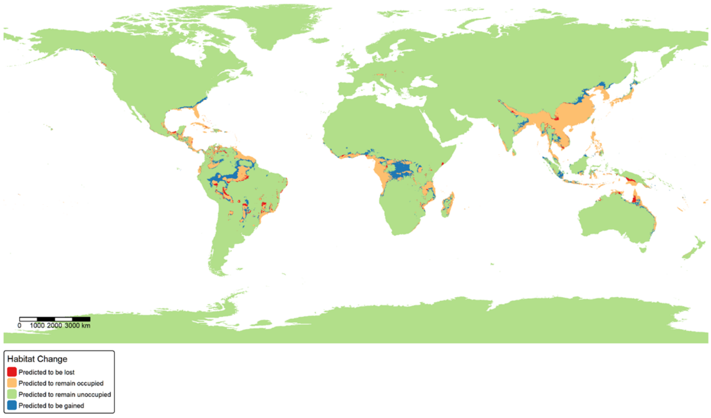

MPI-ESM1-2-HR ssp245 (2041–2060) – Habitat Change

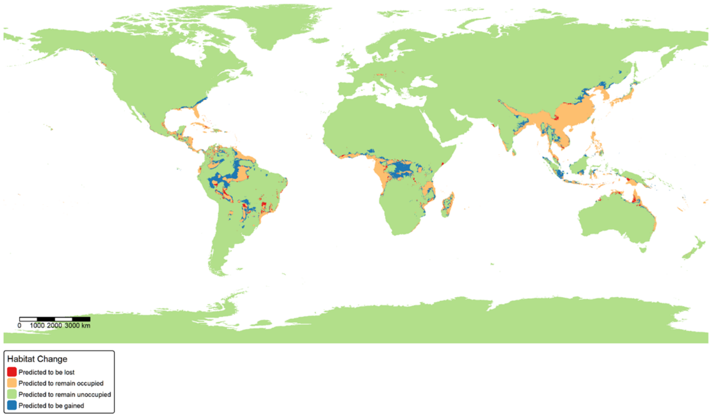

MPI-ESM1-2-HR ssp245 (2061–2080) – Habitat Change

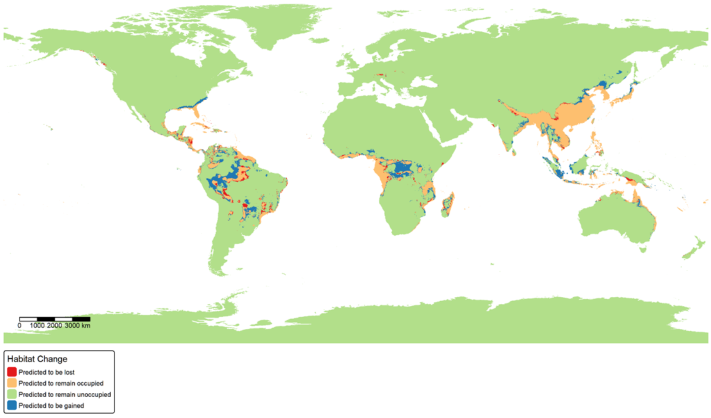

MPI-ESM1-2-HR ssp245 (2081–2100) – Habitat Change

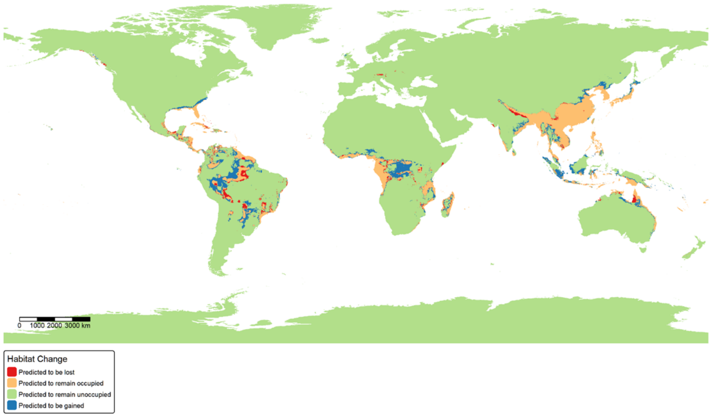

MPI-ESM1-2-HR ssp585 (2021–2040) – Habitat Change

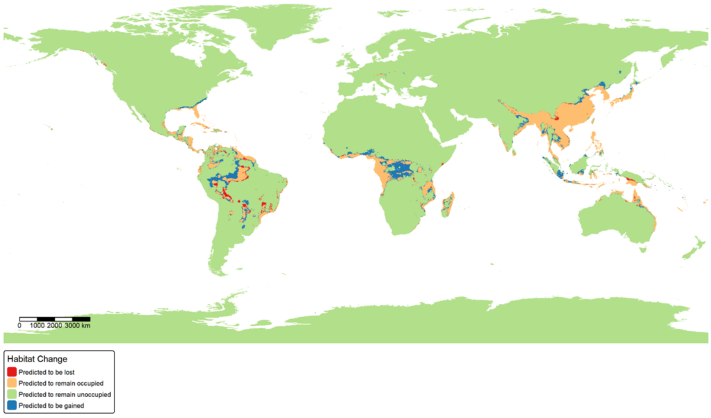

MPI-ESM1-2-HR ssp585 (2041–2060) – Habitat Change

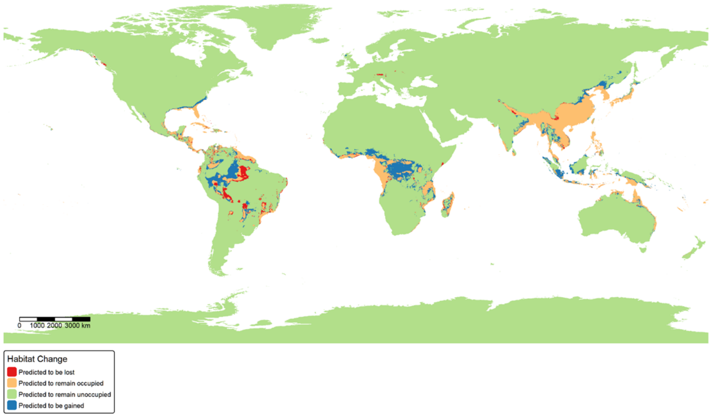

MPI-ESM1-2-HR ssp585 (2061–2080) – Habitat Change

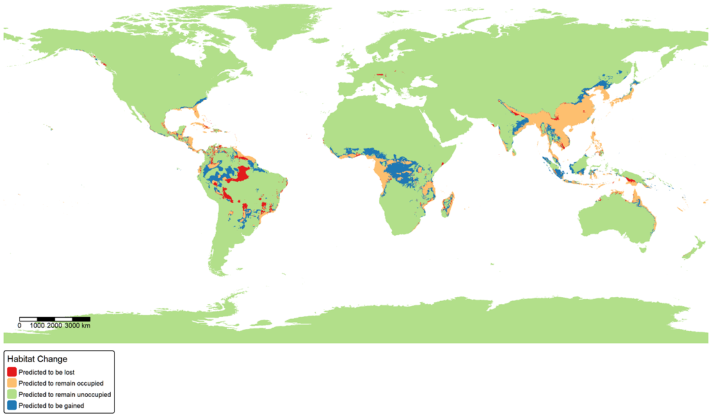

MPI-ESM1-2-HR ssp585 (2081–2100) – Habitat Change

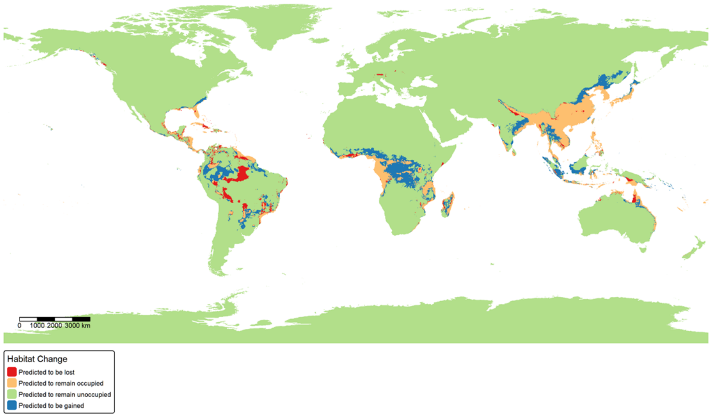

Native Range Projections

MRI-ESM2-0 ssp245 (2021–2040) – Habitat Change

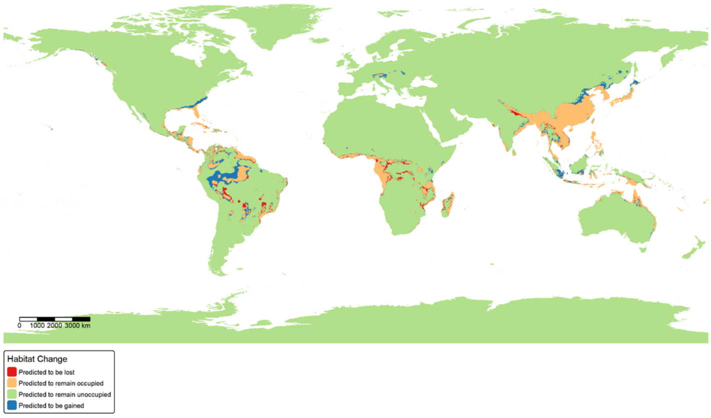

MRI-ESM2-0 ssp245 (2041–2060) – Habitat Change

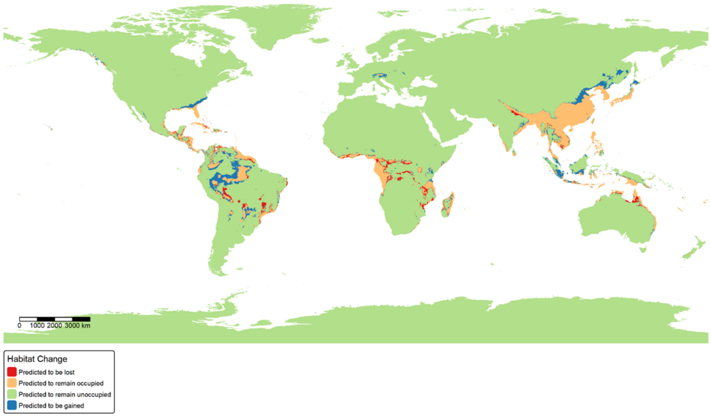

MRI-ESM2-0 ssp245 (2061–2080) – Habitat Change

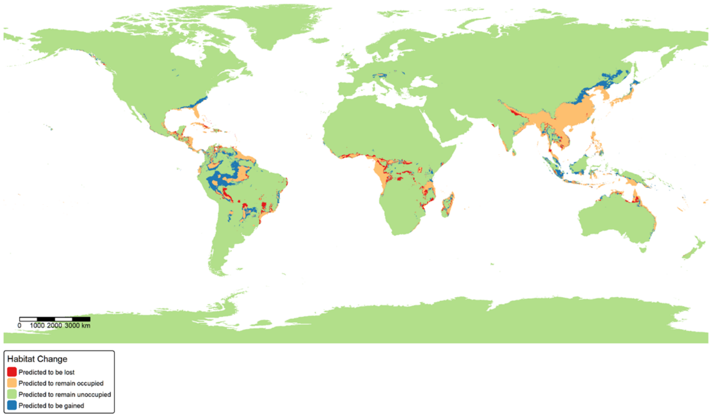

MRI-ESM2-0 ssp245 (2081–2100) – Habitat Change

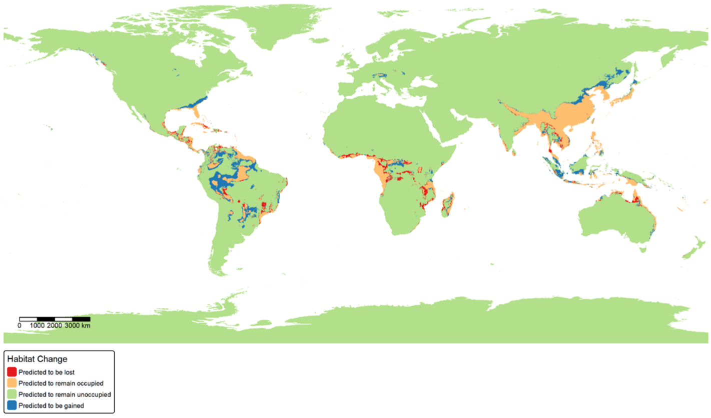

MRI-ESM2-0 ssp585 (2021–2040) – Habitat Change

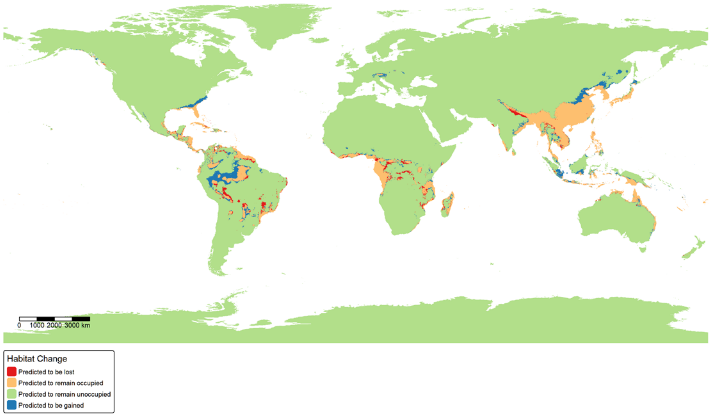

MRI-ESM2-0 ssp585 (2041–2060) – Habitat Change

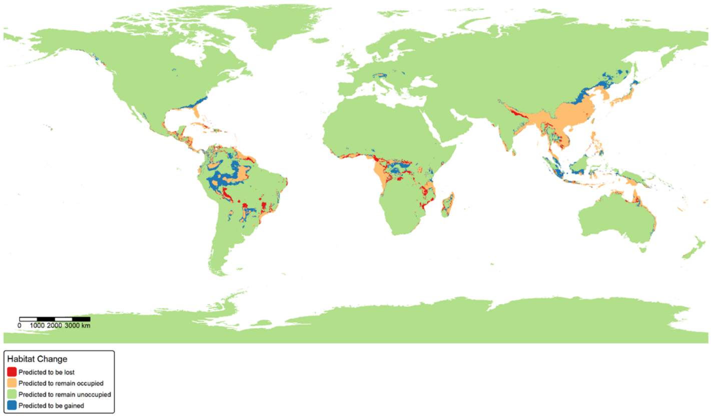

MRI-ESM2-0 ssp585 (2061–2080) – Habitat Change

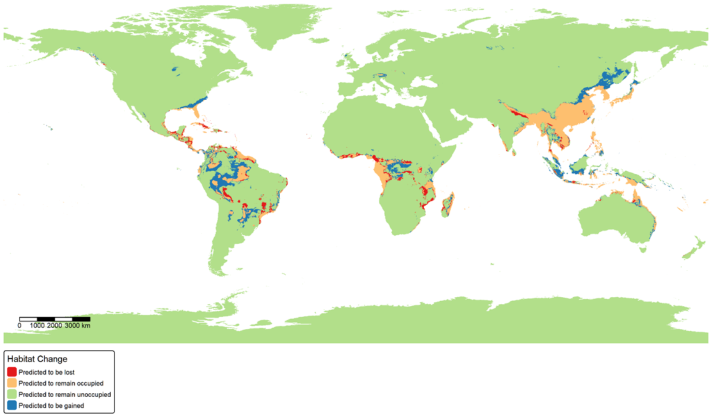

MRI-ESM2-0 ssp585 (2081–2100) – Habitat Change

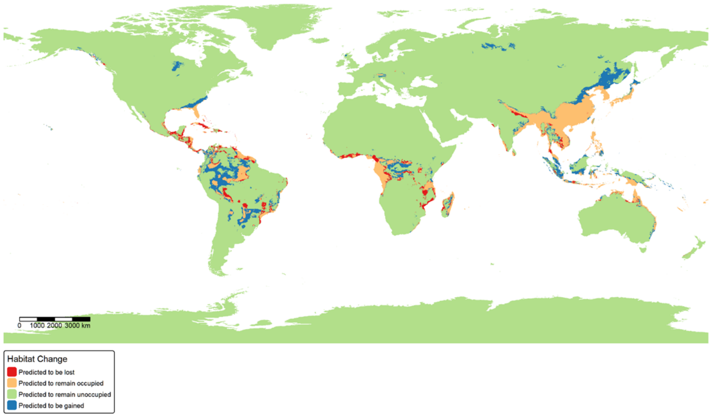

Expanded Range Projections

EC-Earth3-Veg ssp245 (2021–2040) – Habitat Change

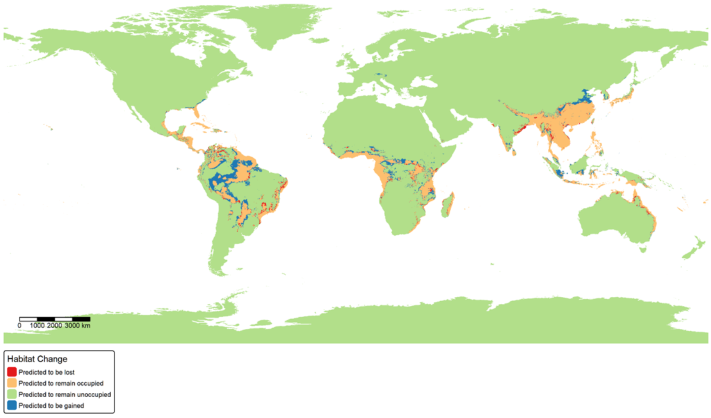

EC-Earth3-Veg ssp245 (2041–2060) – Habitat Change

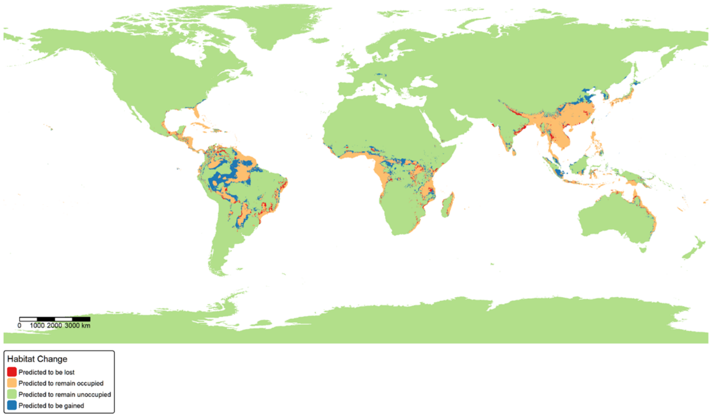

EC-Earth3-Veg ssp245 (2061–2080) – Habitat Change

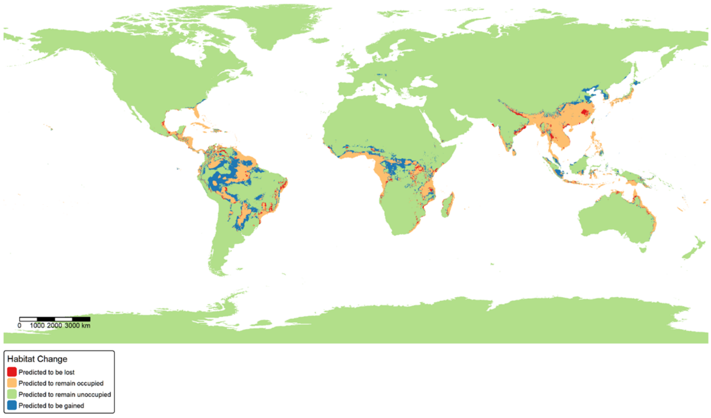

EC-Earth3-Veg ssp245 (2081–2100) – Habitat Change

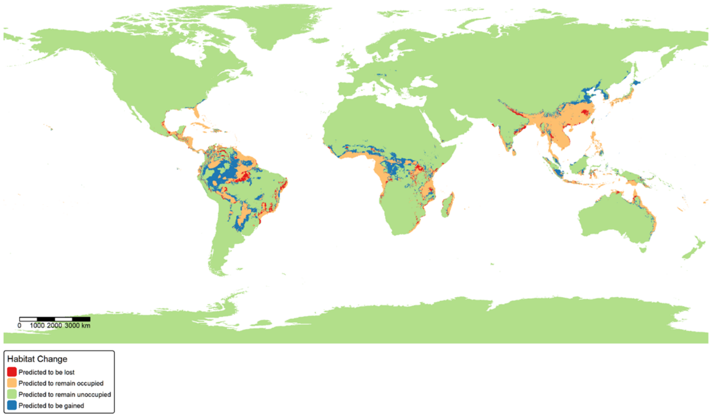

EC-Earth3-Veg ssp585 (2021–2040) – Habitat Change

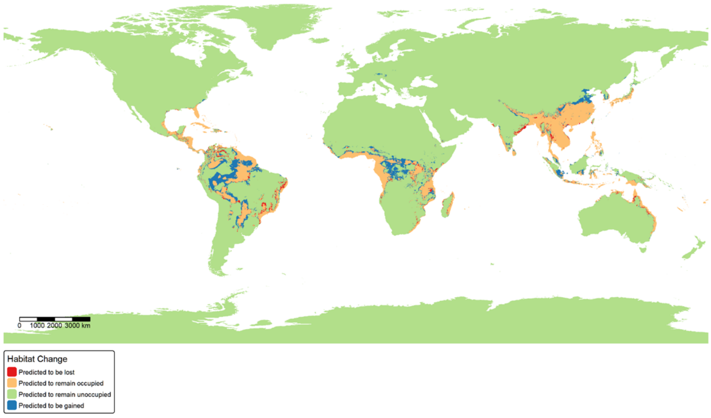

EC-Earth3-Veg ssp585 (2041–2060) – Habitat Change

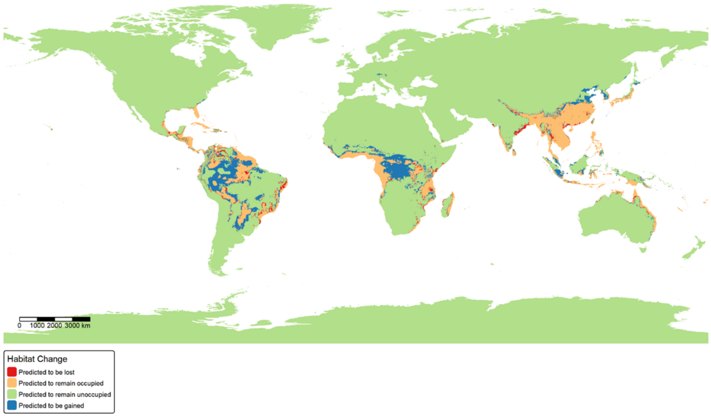

EC-Earth3-Veg ssp585 (2061–2080) – Habitat Change

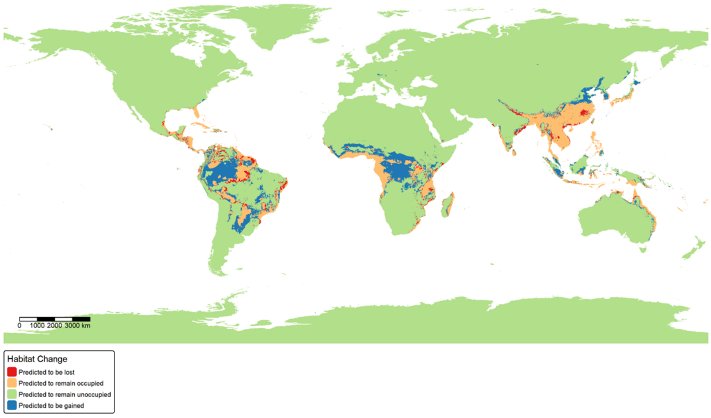

EC-Earth3-Veg ssp585 (2081–2100) – Habitat Change

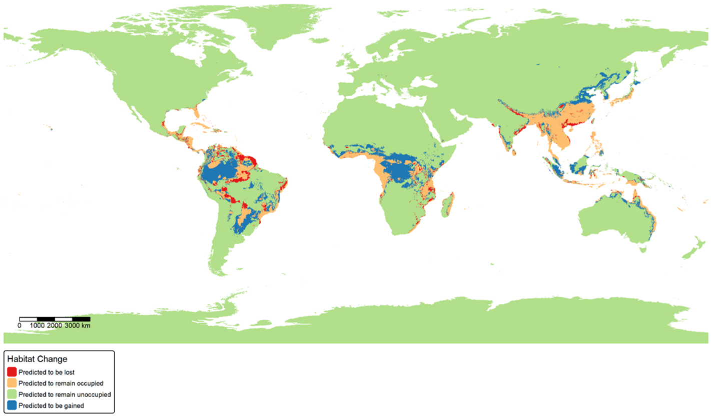

Expanded Range Projections

MPI-ESM1-2-HR ssp245 (2021–2040) – Habitat Change

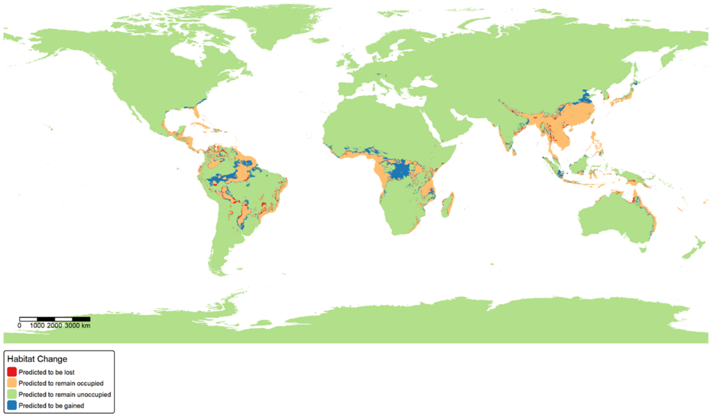

MPI-ESM1-2-HR ssp245 (2041–2060) – Habitat Change

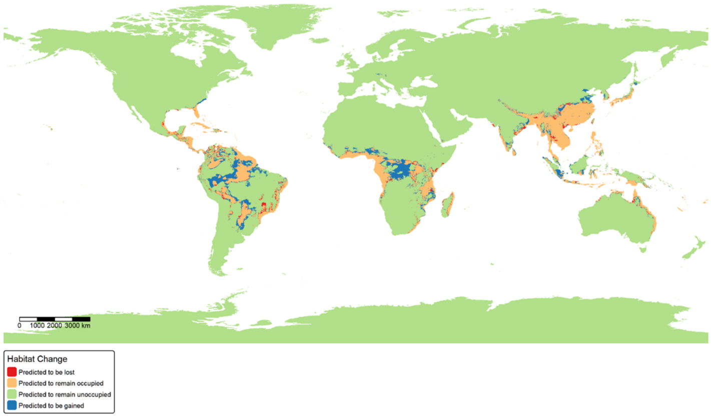

MPI-ESM1-2-HR ssp245 (2061–2080) – Habitat Change

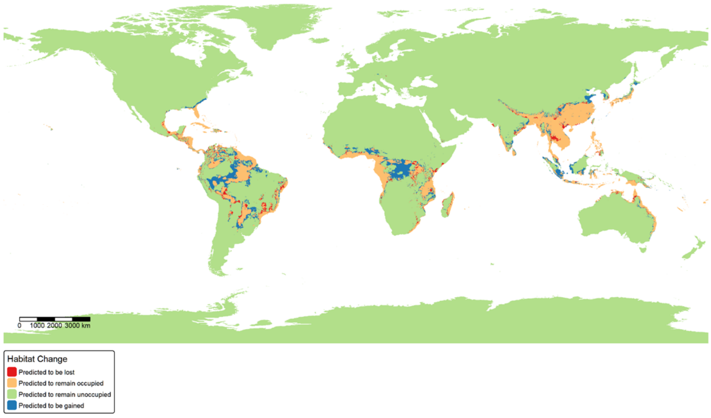

MPI-ESM1-2-HR ssp245 (2081–2100) – Habitat Change

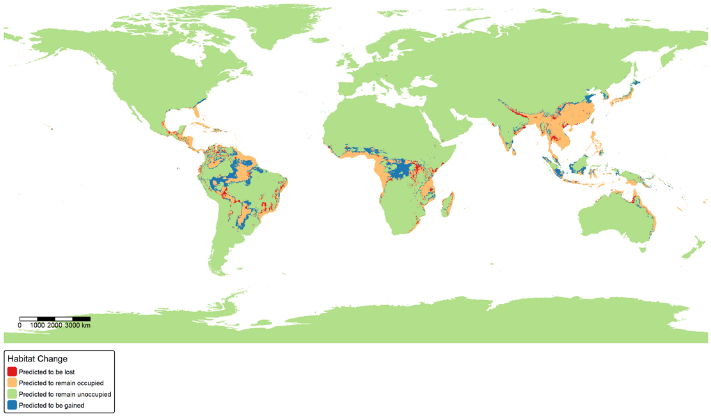

MPI-ESM1-2-HR ssp585 (2021–2040) – Habitat Change

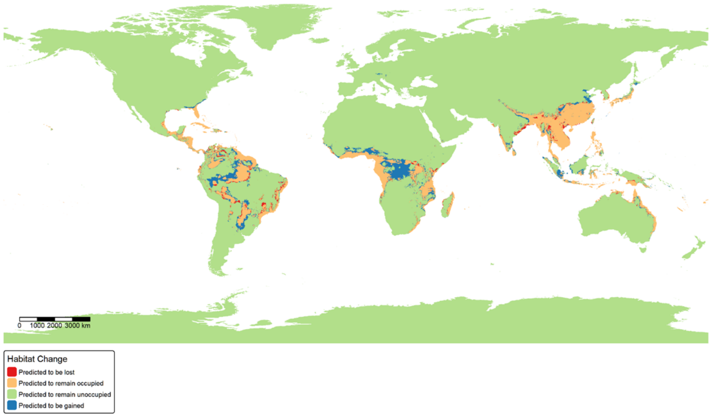

MPI-ESM1-2-HR ssp585 (2041–2060) – Habitat Change

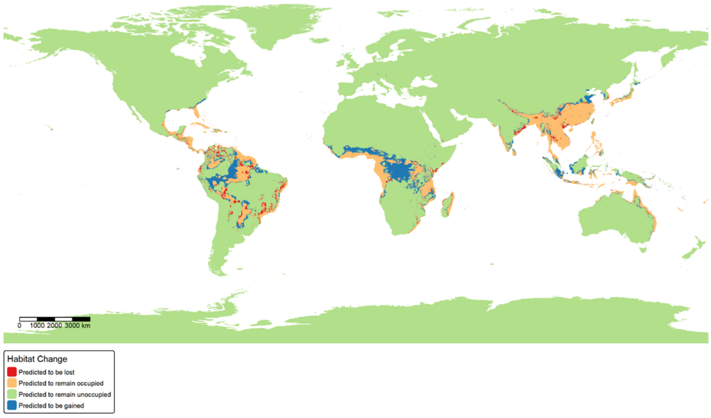

MPI-ESM1-2-HR ssp585 (2061–2080) – Habitat Change

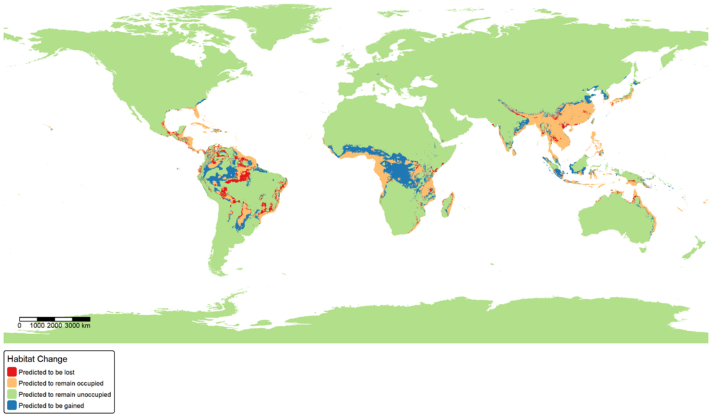

MPI-ESM1-2-HR ssp585 (2081–2100) – Habitat Change

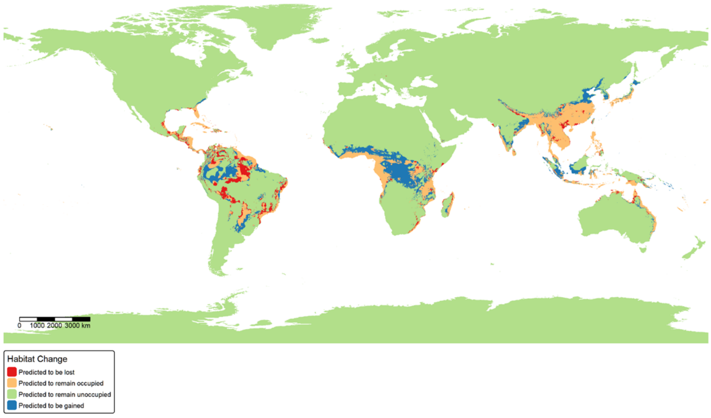

Expanded Range Projections

MRI-ESM2-0 ssp245 (2021–2040) – Habitat Change

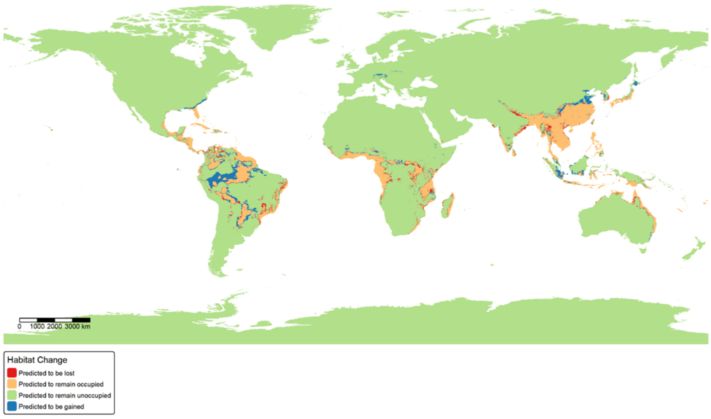

MRI-ESM2-0 ssp245 (2041–2060) – Habitat Change

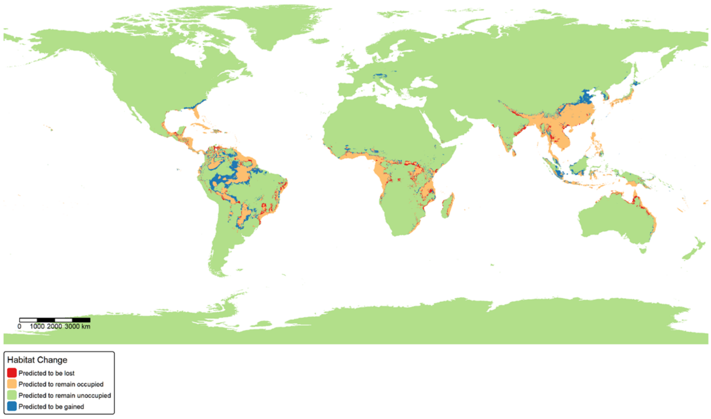

MRI-ESM2-0 ssp245 (2061–2080) – Habitat Change

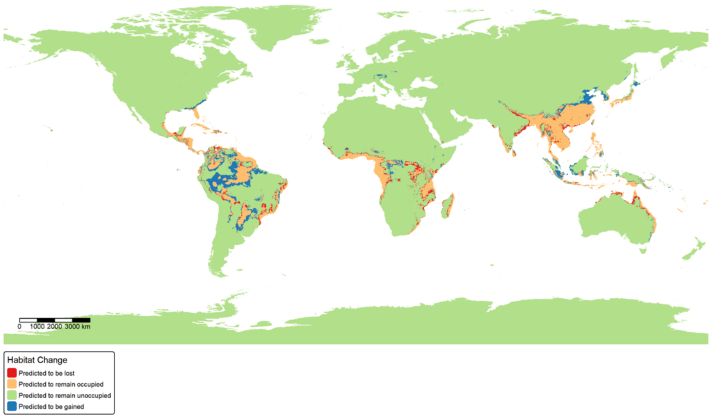

MRI-ESM2-0 ssp245 (2081–2100) – Habitat Change

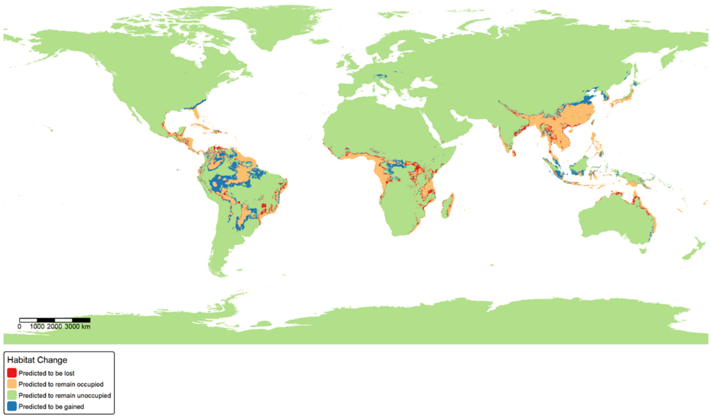

MRI-ESM2-0 ssp585 (2021–2040) – Habitat Change

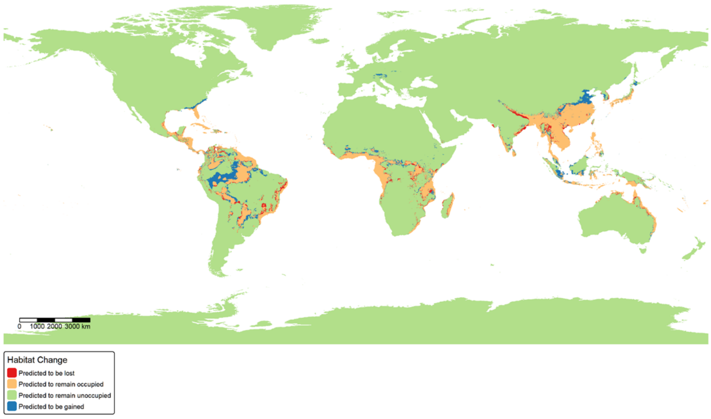

MRI-ESM2-0 ssp585 (2041–2060) – Habitat Change

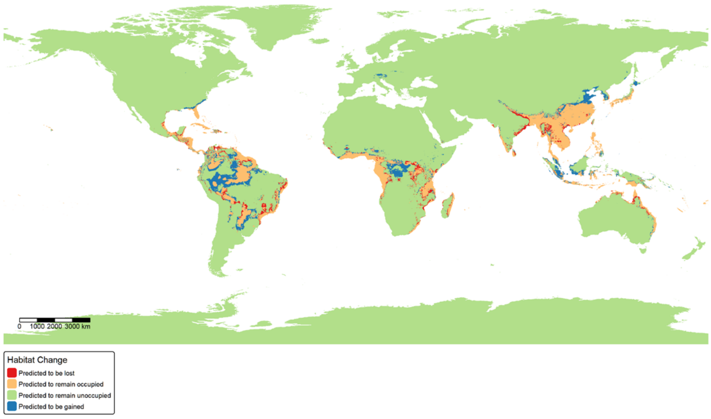

MRI-ESM2-0 ssp585 (2061–2080) – Habitat Change

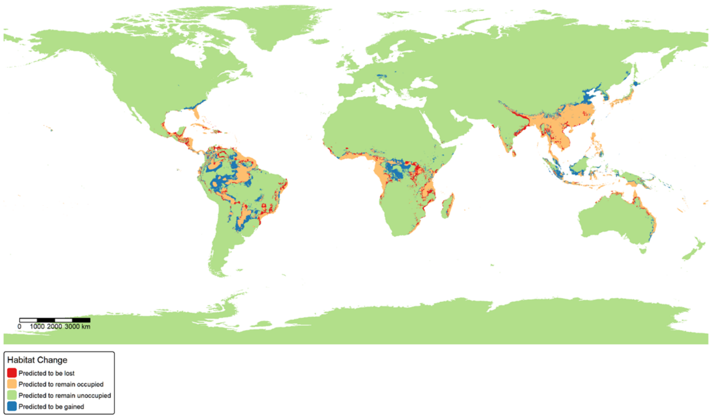

MRI-ESM2-0 ssp585 (2081–2100) – Habitat Change

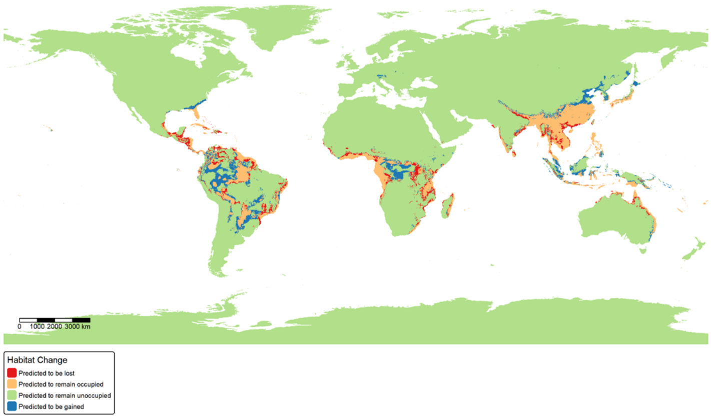

Supplement: Supplementary file 9 — Figure S9: Range change maps of all 48 projected future climatic models compared by pixel difference with current native and expanded ensemble models for T. floralis. Allowing for a visual representation of loss or gain in potential distribution. [file ECE3-16-e73838-s014.pdf]
