# Supplementary material for: Invasion Patterns and Niche Dynamics of the Pollinivorous Florida Calligrapher, Toxomerus floralis (Diptera: Syrphidae) in the Afrotropical Region
Source: Ecol Evol. 2026 Jun 23;16(6):e73838. doi: 10.1002/ece3.73838 (PMC13288174; doi:10.1002/ece3.73838)
Supplement: Supplementary file 11 — Table S1: Niche dynamic indices and statistical significance tests for niche conservatism. [file ECE3-16-e73838-s025.docx]

**Supplementary materials Table S1.**

**Table S1:** Niche dynamic indices and statistical significance tests for niche conservatism

| Metric | Observed Value | p-value |
| --- | --- | --- |
| Schoener's D | 0.2748 | 0.0200 |
| Hellinger's I | 0.5120 | 0.0140 |
| Expansion | 0.2363 | 0.0480 |
| Stability | 0.7637 | 0.0480 |
| Unfilling | 0.0116 | 0.0255 |

*Note: Statistical significance was assessed using ecospat.niche.similarity.test with 2000 replications to test for niche conservatism.*

**README**

**Metric definitions
Schoener’s D:** Measure of niche overlap between two distributions, ranging from 0 (no overlap) to 1 (complete overlap).

**Hellinger’s I:** Alternative metric of niche overlap, also ranging from 0 (no overlap) to 1 (complete overlap).

**Expansion:** Proportion of the invaded niche that occupies environmental conditions not present in the native range.

**Stability:**Proportion of the invaded niche that overlaps with environmental conditions in the native range.

**Unfilling:**Proportion of the native niche not occupied in the invaded range.
