# Supplementary material for: Invasion Patterns and Niche Dynamics of the Pollinivorous Florida Calligrapher, Toxomerus floralis (Diptera: Syrphidae) in the Afrotropical Region
Source: Ecol Evol. 2026 Jun 23;16(6):e73838. doi: 10.1002/ece3.73838 (PMC13288174; doi:10.1002/ece3.73838)
Supplement: Supplementary file 12 — Data S1: Museums or private collections visited to look for specimens of T. floralis . [file ECE3-16-e73838-s027.docx]

**Data S1**: Museums or private collections visited to look for specimens of *T. floralis*.

AMGS : Albany Museum, Makhanda (formerly Grahamstown), South Africa

AMNH : American Museum of Natural History, New York, USA

ASPC : Axel Ssymank Personal Collection, Germany

BMSA : National Museum, Bloemfontein, South Africa

CAS : California Academy of Sciences, San Francisco, USA

CIRAD : French Agricultural Research Centre for International Development, La Réunion, France

CNC : Canadian National Collection of Insects, Arachnids and Nematodes, Ottawa, Canada

CSCA : California State Collection of Arthropods, Sacramento, USA

DMSA : Durban Natural Science Museum, Durban, South Africa

FSUNS : Faculty of Sciences, University of Novi Sad, Serbia

*icipe* : International Centre of Insect Physiology and Ecology, Nairobi, Kenya

IITA : International Institute of Tropical Agriculture, Cotonou, Benin

RBINS : Royal Belgian Institute of Natural Sciences, Brussels, Belgium

RMCA : Royal Museum for Central Africa, Tervuren, Belgium

MZH : Finnish Museum of Natural History, University of Helsinki, Finland

MZLU : Zoological Museum, Lund University, Lund, Sweden

NHMUK : Natural History Museum UK, London, UK (formerly BMNH)

NHRS : Naturhistoriska Riksmuseet Stockholm, Stockholm, Sweden

NMKE : National Museums of Kenya, Nairobi, Kenya

NMSA : KwaZulu-Natal Museum, Pietermaritzburg, South Africa (formerly Natal Museum, South Africa)

RMNH : Naturalis Biodiversity Center, Leiden, The Netherlands

SAMC : Iziko Museum of South Africa, Cape Town, South Africa (formerly South African Museum, Cape town)

USNM : National Museum of Natural History, Smithsonian Institution, Washington D.C., USA

ZFMK : Museum Koenig Bonn, LIB, Bonn, Germany

There are some other major collections of Afrotropical Syrphidae which we did not screen entirely (ANSP: Academy of Natural Sciences of Philadelphia, Philadelphia, USA; MCSNG: Museo Civico di Storia Naturale “Giacomo Doria”, Genoa, Italy; MCZ: Museum of Comparative Zoology, Cambridge, USA; NHMB: Naturhistorisches Museum, Basel, Switzerland; NHMW: Naturhistorisches Museum Wien, Vienna, Austria; MNB: Museum für Naturkunde – Leibniz-Institut für Evolutions- und Biodiversitätsforschung, Berlin, Germany; MNHN: Muséum national d'Histoire naturelle, Paris, France) but no literature or reports of Afrotropical expeditions with material deposited at those repositories report on *T. floralis*. We assume that it is very unlikely that any of these holdings have *T. floralis* from outside its native range, especially since these Afrotropical hover fly collections are from old expeditions from before introduction into the Region.
