# Supplementary material for: Invasion Patterns and Niche Dynamics of the Pollinivorous Florida Calligrapher, Toxomerus floralis (Diptera: Syrphidae) in the Afrotropical Region
Source: Ecol Evol. 2026 Jun 23;16(6):e73838. doi: 10.1002/ece3.73838 (PMC13288174; doi:10.1002/ece3.73838)
Supplement: Supplementary file 15 — Data S4: Traditional modelling techniques, abbreviations and full names, based on information as provided by the ‘biomod2’ package (Guéguen et al. 2025). [file ECE3-16-e73838-s004.docx]

| **Model abbreviation** | **Modelling technique full name** |
| --- | --- |
| ANN | Artificial Neural Network (nnet) |
| CTA | Classification Tree Analysis (rpart) |
| FDA | Flexible Discriminant Analysis (fda) |
| GAM | Generalized Additive Model (gam, gam or bam) |
| GBM | Generalized Boosting Model, or usually called Boosted Regression Trees (gbm) |
| GLM | Generalized Linear Model (glm) |
| MARS | Multiple Adaptive Regression Splines (earth) |
| MAXENT | Maximum Entropy (maxent) |
| MAXNET | Maximum Entropy (maxnet) |
| RF | Random Forest (randomForest) |
| RFd | Random Forest downsampled (randomForest) |
| SRE | Surface Range Envelop or usually called BIOCLIM (bm_SRE) |
| XGBOOST | eXtreme Gradient Boosting Training (xgboost) |

**Data S4**: Tradition modelling techniques, abbreviations and full names, based on information as provided by the biomod2 package (Guéguen et al. 2025)
