# Supplementary material for: Invasion Patterns and Niche Dynamics of the Pollinivorous Florida Calligrapher, Toxomerus floralis (Diptera: Syrphidae) in the Afrotropical Region
Source: Ecol Evol. 2026 Jun 23;16(6):e73838. doi: 10.1002/ece3.73838 (PMC13288174; doi:10.1002/ece3.73838)
Supplement: Supplementary file 26 — Data S15: R‐script used in generating predictive models, projections and figures. [file ECE3-16-e73838-s019.docx]

**#Ensemble Niche modelling of Native, invaded and expanded ranges of *Toxomerus floralis***

library(ecospat)

library(terra)

library(rgbif)

library(geodata)

library(ade4)

library(raster)

library(biomod2)

library(grid)

library(gridExtra)

##read all occurrence points into dataframe

toxomerusData <- read.csv("toxomerus_all.csv", sep = ",", header = TRUE)

#thinning of records Toxomerus floralis to match spatial resolution of climatic data

thinned_dataset_full <-

thin( loc.data = toxomerusData,

lat.col = "decimalLatitude", long.col = "decimalLongitude",

spec.col = "species1",

thin.par = 10, reps = 100,

locs.thinned.list.return = TRUE,

write.files = TRUE,

max.files = 5,

out.dir = "floralis_thinned_full/", out.base = " toxomerusData_thinned",

write.log.file = TRUE,

log.file = "floralis_thinned_full_log_file.txt" )

#Create rasterstack of cropped native range variables

setwd("./Niche modelling/wc2.1_5m_bio/native_current")

file_list <- list.files(pattern = "wc", full.names = TRUE)

bioclim_5m_native <- stack(file_list, RAT = FALSE)

#Create rasterstack of cropped invaded range variables

setwd("./Niche modelling/wc2.1_5m_bio/invaded_current")

file_list <- list.files(pattern = "wc", full.names = TRUE)

bioclim_5m_invaded <- stack(file_list, RAT = FALSE)

#Create rasterstack of cropped expanded range variables

setwd("./Niche modelling/wc2.1_5m_bio/expanded_current")

file_list <- list.files(pattern = "wc", full.names = TRUE)

bioclim_5m_expanded <- stack(file_list, RAT = FALSE)

#creation of dataframes of bioclimatic variables for PCA analysis to test for co-linearity

bioclim_5m_native_df <- na.omit(as.data.frame(bioclim_5m_native))

bioclim_5m_invaded_df <- na.omit(as.data.frame(bioclim_5m_invaded))

bioclim_5m_expanded_df <- na.omit(as.data.frame(bioclim_5m_expanded))

pca_native <- dudi.pca(bioclim_5m_native_df, scannf = FALSE, nf = 2)

pca_invaded <- dudi.pca(bioclim_5m_invaded_df, scannf = FALSE, nf = 2)

pca_expanded <- dudi.pca(bioclim_5m_expanded_df, scannf = FALSE, nf = 2)

##PCA plot

plot(pca_native$li[, 1:2])

plot(pca_invaded$li[, 1:2])

plot(pca_expanded$li[, 1:2])

#Remove outliers native

tail_scores <- sort(pca_native$li[, 1])[1:10]

tail_scores

to_remove <- which(pca_native$li[, 1] < -10)

to_remove

if (length(to_remove)) {

bioclim_5m_native_df <- bioclim_5m_native_df [-to_remove, ]

pca_native <- dudi.pca(bioclim_5m_native_df, scannf = FALSE, nf = 2)

}

plot(pca_native$li[, 1:2])

#Remove outliers invaded

tail_scores <- sort(pca_invaded$li[, 1])[1:10]

tail_scores

to_remove <- which(pca_invaded$li[, 1] < -10)

to_remove

if (length(to_remove)) {

bioclim_5m_invaded_df <- bioclim_5m_invaded_df [-to_remove, ]

pca_invaded <- dudi.pca(bioclim_5m_invaded_df, scannf = FALSE, nf = 2)

}

plot(pca_invaded$li[, 1:2])

#Remove outliers expanded

tail_scores <- sort(pca_expanded$li[, 1])[1:10]

tail_scores

to_remove <- which(pca_expanded$li[, 1] < -10)

to_remove

if (length(to_remove)) {

bioclim_5m_expanded_df <- bioclim_5m_expanded_df [-to_remove, ]

pca_expanded <- dudi.pca(bioclim_5m_expanded_df, scannf = FALSE, nf = 2)

}

plot(pca_expanded$li[, 1:2])

#Create dataframes

toxomerusData_native <- read.csv("toxomerusData_native.csv", sep = ",", header = TRUE)

toxomerusData_invaded <- read.csv("toxomerusData_invaded.csv", sep = ",", header = TRUE)

toxomerusData_native <- read.csv("toxomerusData_native.csv", sep = ",", header = TRUE)

#Create spatialdataframes

toxomerusData_native_sp <- SpatialPointsDataFrame(coords = toxomerusData_native[, c("decimalLongitude", "decimalLatitude")], data = toxomerusData_native)

toxomerusData_invaded_sp <- SpatialPointsDataFrame(coords = toxomerusData_invaded[, c("decimalLongitude", "decimalLatitude")], data = toxomerusData_invaded)

toxomerusData_expanded_sp <- SpatialPointsDataFrame(coords = toxomerusData_expanded[, c("decimalLongitude", "decimalLatitude")], data = toxomerusData_expanded)

Toxomerus_native_cell_id <- cellFromXY(subset(bioclim_5m_native, 1), toxomerusData_native_sp)

Toxomerus_invaded_cell_id <- cellFromXY(subset(bioclim_5m_invaded, 1), toxomerusData_invaded_sp)

Toxomerus_expanded_cell_id <- cellFromXY(subset(bioclim_5m_expanded, 1), toxomerusData_expanded_sp)

#native PCA final run

#Set up a 1x2 plotting layout

par(mfrow = c(1, 2))

#Plot PCA scores discriminating Toxomerus floralis presences from the entire native environmental space

s.class(pca_native$li[, 1:2],

fac = factor(rownames(bioclim_5m_native_df) %in% Toxomerus_native_cell_id,

levels = c("FALSE", "TRUE"),

labels = c("background", "Toxomerusfloralis")),

col = c("red", "blue"),

csta = 0,

cellipse = 2,

cpoint = .3,

pch = 16)

mtext("(a)", side = 3, line = 3, adj = 0)

s.corcircle(pca_native$co, clabel = 0.5)

mtext("(b)", side = 3, line = 3, adj = 0)

#invaded PCA final run

#Set up a 1x2 plotting layout

par(mfrow = c(1, 2))

#Plot PCA scores discriminating Toxomerus floralis presences from the entire invaded environmental space

s.class(pca_invaded$li[, 1:2],

fac = factor(rownames(bioclim_5m_invaded_df) %in% Toxomerus_invaded_cell_id,

levels = c("FALSE", "TRUE"),

labels = c("background", "Toxomerusfloralis")),

col = c("red", "blue"),

csta = 0,

cellipse = 2,

cpoint = .3,

pch = 16)

mtext("(a)", side = 3, line = 3, adj = 0)

s.corcircle(pca_invaded$co, clabel = 0.5)

mtext("(b)", side = 3, line = 3, adj = 0)

#expanded PCA final run

#Set up a 1x2 plotting layout

par(mfrow = c(1, 2))

#Plot PCA scores discriminating Toxomerus floralis presences from the entire expanded environmental space

s.class(pca_expanded$li[, 1:2],

fac = factor(rownames(bioclim_5m_expanded_df) %in% Toxomerus_expanded_cell_id,

levels = c("FALSE", "TRUE"),

labels = c("background", "Toxomerusfloralis")),

col = c("red", "blue"),

csta = 0,

cellipse = 2,

cpoint = .3,

pch = 16)

mtext("(a)", side = 3, line = 3, adj = 0)

s.corcircle(pca_expanded$co, clabel = 0.5)

mtext("(b)", side = 3, line = 3, adj = 0)

#Variance Inflation Factors (VIF) for each predictor in native range

vif_values <- vif(bioclim_5m_native_df)

#Calculate Variance Inflation Factors (VIF) for each pair of predictor variables in native range

v1 <- vifcor(bioclim_5m_native_df, th = 0.9)

#Apply stepwise variable selection based on Variance Inflation Factors (VIF) for native range

v2 <- vifstep(bioclim_5m_native_df, th = 10)

#Subset the bioclim_5m_native raster stack to include only specific layers as determined by vifstep and also with input from PCA results

bioclim_5m_native_sub <- stack(subset(bioclim_5m_native,

c(“wc2.1_5m_bio_2_native",

"wc2.1_5m_bio_3_native",

"wc2.1_5m_bio_8_native",

"wc2.1_5m_bio_15_native",

"wc2.1_5m_bio_18_native")))

#Subset the bioclim_5m_invaded raster stack to include only specific layers as determined by vifstep and also with input from PCA results

bioclim_5m_invaded_sub <- stack(subset(bioclim_5m_invaded,

c(“wc2.1_5m_bio_2_invaded",

"wc2.1_5m_bio_3_invaded",

"wc2.1_5m_bio_8_invaded",

"wc2.1_5m_bio_15_invaded",

"wc2.1_5m_bio_18_invaded")))

#Subset the bioclim_5m_expanded raster stack to include only specific layers as determined by vifstep and also with input from PCA results

bioclim_5m_expanded_sub <- stack(subset(bioclim_5m_expanded,

c(“wc2.1_5m_bio_2_expanded",

"wc2.1_5m_bio_3_expanded",

"wc2.1_5m_bio_8_expanded",

"wc2.1_5m_bio_15_expanded",

"wc2.1_5m_bio_18_expanded")))

#Extract coordinates from SpatialPoints object; Convert the coordinates to a data frame

coords <- toxomerusData_native@coords

toxomerusData_native_df <- data.frame(

decimalLongitude = coords[, 1],

decimalLatitude = coords[, 2]

)

#Extract coordinates from SpatialPoints object; Convert the coordinates to a data frame

coords <- toxomerusData_invaded@coords

toxomerusData_invaded_df <- data.frame(

decimalLongitude = coords[, 1],

decimalLatitude = coords[, 2]

#Extract coordinates from SpatialPoints object; Convert the coordinates to a data frame

coords <- toxomerusData_expanded@coords

toxomerusData_expanded_df <- data.frame(

decimalLongitude = coords[, 1],

decimalLatitude = coords[, 2]

#Pseudoabsences for toxomerus floralis native data

toxomerusData_native_allPA <- BIOMOD_FormatingData(

resp.var = rep(1, nrow(toxomerusData_native)),

expl.var = bioclim_5m_native_sub,

resp.xy = toxomerusData_native[, c('decimalLongitude', 'decimalLatitude')],

resp.name = "Toxomerus.floralis",

PA.nb.rep = 10,

PA.nb.absences = 705,

PA.strategy = 'random'

)

#Pseudoabsences for toxomerus floralis invaded data

toxomerusData_invaded_allPA <- BIOMOD_FormatingData(

resp.var = rep(1, nrow(toxomerusData_invaded)),

expl.var = bioclim_5m_invaded_sub,

resp.xy = toxomerusData_invaded[, c('decimalLongitude', 'decimalLatitude')],

resp.name = "Toxomerus.floralis",

PA.nb.rep = 10,

PA.nb.absences = 384,

PA.strategy = 'random'

)

#Pseudoabsences for toxomerus floralis expanded data

toxomerusData_expanded_allPA <- BIOMOD_FormatingData(

resp.var = rep(1, nrow(toxomerusData_expanded)),

expl.var = bioclim_5m_expanded_sub,

resp.xy = toxomerusData_expanded[, c('decimalLongitude', 'decimalLatitude')],

resp.name = "Toxomerus.floralis",

PA.nb.rep = 10,

PA.nb.absences = 1089,

PA.strategy = 'random'

)

#Running traditional models on toxomerus floralis native data

myBiomodModelOut_native <- BIOMOD_Modeling(bm.format = toxomerusData_native_allPA,

modeling.id = 'AllModels_native',

CV.strategy = 'random',

CV.nb.rep = 10,

CV.perc = 0.8,

OPT.strategy = 'bigboss',

var.import = 3,

metric.eval = c('TSS','ROC','KAPPA', 'BOYCE'))

#Running traditional models on toxomerus floralis invaded data

myBiomodModelOut_invaded <- BIOMOD_Modeling(bm.format = toxomerusData_invaded_allPA,

modeling.id = 'AllModels_invaded',

CV.strategy = 'random',

CV.nb.rep = 10,

CV.perc = 0.8,

OPT.strategy = 'bigboss',

var.import = 3,

metric.eval = c('TSS','ROC','KAPPA', 'BOYCE'))

#Running traditional models on toxomerus floralis expanded data

myBiomodModelOut_expanded <- BIOMOD_Modeling(bm.format = toxomerusData_expanded_allPA,

modeling.id = 'AllModels_expanded',

CV.strategy = 'random',

CV.nb.rep = 10,

CV.perc = 0.8,

OPT.strategy = 'bigboss',

var.import = 3,

metric.eval = c('TSS','ROC','KAPPA', 'BOYCE'))

#Running EM model for toxomerus floralis native data

myBiomodEM_native <- BIOMOD_EnsembleModeling(bm.mod = myBiomodModelOut_native,

models.chosen = 'all',

em.by = 'all', em.algo = c('EMmean', 'EMcv', 'EMci', 'EMmedian', 'EMca', 'EMwmean'),

metric.select = c('TSS'),

metric.select.thresh = c(0.7),

metric.eval = c('TSS', 'ROC', 'KAPPA', 'BOYCE'),

var.import = 3,

EMci.alpha = 0.05,

EMwmean.decay = 'proportional')

#Running EM model for toxomerus floralis invaded data

myBiomodEM_invaded <- BIOMOD_EnsembleModeling(bm.mod = myBiomodModelOut_invaded,

models.chosen = 'all',

em.by = 'all', em.algo = c('EMmean', 'EMcv', 'EMci', 'EMmedian', 'EMca', 'EMwmean'),

metric.select = c('TSS'),

metric.select.thresh = c(0.7),

metric.eval = c('TSS', 'ROC', 'KAPPA', 'BOYCE'),

var.import = 3,

EMci.alpha = 0.05,

EMwmean.decay = 'proportional')

#Running EM model for toxomerus floralis expanded data

myBiomodEM_expanded <- BIOMOD_EnsembleModeling(bm.mod = myBiomodModelOut_expanded,

models.chosen = 'all',

em.by = 'all', em.algo = c('EMmean', 'EMcv', 'EMci', 'EMmedian', 'EMca', 'EMwmean'),

metric.select = c('TSS'),

metric.select.thresh = c(0.7),

metric.eval = c('TSS', 'ROC', 'KAPPA', 'BOYCE'),

var.import = 3,

EMci.alpha = 0.05,

EMwmean.decay = 'proportional')

#explanatory environmental variables for current conditions used for projection

myExpl_native <- terra::rast(bioclim_5m_native_sub)

myExpl_invaded <- terra::rast(bioclim_5m_invaded_sub)

myExpl_expanded <- terra::rast(bioclim_5m_expanded_sub)

myBiomodEMProj_native <- BIOMOD_EnsembleForecasting(bm.em = myBiomodEM_native,

proj.name = 'CurrentEM_native',

new.env = myExpl_native,

models.chosen = 'all',

metric.binary = 'all',

metric.filter = 'all')

myBiomodEMProj_invaded <- BIOMOD_EnsembleForecasting(bm.em = myBiomodEM_invaded,

proj.name = 'CurrentEM_invaded',

new.env = myExpl_invaded,

models.chosen = 'all',

metric.binary = 'all',

metric.filter = 'all')

myBiomodEMProj_expanded <- BIOMOD_EnsembleForecasting(bm.em = myBiomodEM_expanded,

proj.name = 'CurrentEM_expanded',

new.env = myExpl_expanded,

models.chosen = 'all',

metric.binary = 'all',

metric.filter = 'all')

#The Continuous Boyce Index (CBI)

obs2 <- subset(toxomerusData_native, select = -Species1)

obs <- subset(toxomerusData_expanded, select = -Species1)

#CBI for native range

boyce_native <- ecospat.boyce(EMprojrast_native, obs2, nclass = 0,

window.w = "default", res = 100,

PEplot = FALSE, rm.duplicate = TRUE,

method = 'spearman')

#Creation of Boyce index figure for native projection

hs <- boyce_native$HS #Uppercase HS based on your previous output

F.ratio <- boyce_native$F.ratio

rs <- round(boyce_native$cor, 3) #Rounded Spearman correlation

threshold_index <- which(F.ratio >= 1)[1]

threshold_hs <- hs[threshold_index]

plot(hs, F.ratio, type = "n",

xlab = "Habitat suitability",

ylab = "Predicted/Expected ratio",

main = "Native Range")

rect(min(hs), 0, threshold_hs, max(F.ratio)*1.1,

col = rgb(1, 0.8, 0.8), border = NA)

band_width <- (max(hs) - min(hs)) * 0.03

rect(threshold_hs, 0, threshold_hs + band_width, max(F.ratio)*1.1,

col = "gray80", border = NA)

rect(threshold_hs + band_width, 0, max(hs), max(F.ratio)*1.1,

col = rgb(0.8, 1, 0.8), border = NA)

abline(h = 1, col = "red", lty = 2)

points(hs, F.ratio, pch = 16, cex = 0.8)

text(min(hs) + (threshold_hs - min(hs))/2, max(F.ratio)*0.8,

"Not-suitable", cex = 0.8)

text(threshold_hs + band_width/2, max(F.ratio)*0.8,

"P/E = 1", cex = 0.8, srt = 90)

text(threshold_hs + (max(hs) - threshold_hs)/2, max(F.ratio)*0.8,

"Suitable", cex = 0.8)

text(max(hs)*0.85, max(F.ratio)*0.95,

paste("R_s =", rs), cex = 0.8)

text(max(hs)*0.6, 1.5,

"P/E = 1, random model threshold", cex = 0.7, col = "red")

box()

#CBI for expanded range

boyce_expanded <- ecospat.boyce(EMprojrast_expanded, obs, nclass = 0,

window.w = "default", res = 100,

PEplot = FALSE, rm.duplicate = TRUE,

method = 'spearman')

#Creation of Boyce index figure for expanded projection

hs <- boyce_expanded$HS #Uppercase HS based on your previous output

F.ratio <- boyce_expanded$F.ratio

rs <- round(boyce_expanded$cor, 3) #Rounded Spearman correlation

threshold_index <- which(F.ratio >= 1)[1]

threshold_hs <- hs[threshold_index]

plot(hs, F.ratio, type = "n",

xlab = "Habitat suitability",

ylab = "Predicted/Expected ratio",

main = "Expanded Range")

rect(min(hs), 0, threshold_hs, max(F.ratio)*1.1,

col = rgb(1, 0.8, 0.8), border = NA)

band_width <- (max(hs) - min(hs)) * 0.03

rect(threshold_hs, 0, threshold_hs + band_width, max(F.ratio)*1.1,

col = "gray80", border = NA)

rect(threshold_hs + band_width, 0, max(hs), max(F.ratio)*1.1,

col = rgb(0.8, 1, 0.8), border = NA)

abline(h = 1, col = "red", lty = 2)

points(hs, F.ratio, pch = 16, cex = 0.8)

text(min(hs) + (threshold_hs - min(hs))/2, max(F.ratio)*0.8,

"Not-suitable", cex = 0.8)

text(threshold_hs + band_width/2, max(F.ratio)*0.8,

"P/E = 1", cex = 0.8, srt = 90)

text(threshold_hs + (max(hs) - threshold_hs)/2, max(F.ratio)*0.8,

"Suitable", cex = 0.8)

text(max(hs)*0.85, max(F.ratio)*0.95,

paste("R_

s =", rs), cex = 0.8)

text(max(hs)*0.6, 1.5,

"P/E = 1, random model threshold", cex = 0.7, col = "red")

box()

#explanatory environmental variables for future conditions used for projection

#EC-Earth3-Veg ssp245

myExpl_EC_Earth3_Veg_ssp245_2021_2040 <- `wc2.1_5m_Bio_EC-Earth3-Veg_ssp245_2021-2040`

myExpl_EC_Earth3_Veg_ssp245_2041_2060 <- `wc2.1_5m_Bio_EC-Earth3-Veg_ssp245_2041-2060`

myExpl_EC_Earth3_Veg_ssp245_2061_2080 <- `wc2.1_5m_Bio_EC-Earth3-Veg_ssp245_2061-2080`

myExpl_EC_Earth3_Veg_ssp245_2081_2100 <- `wc2.1_5m_Bio_EC-Earth3-Veg_ssp245_2081-2100`

#EC-Earth3-Veg ssp585

myExpl_EC_Earth3_Veg_ssp585_2021_2040 <- `wc2.1_5m_Bio_EC-Earth3-Veg_ssp585_2021-2040`

myExpl_EC_Earth3_Veg_ssp585_2041_2060 <- `wc2.1_5m_Bio_EC-Earth3-Veg_ssp585_2041-2060`

myExpl_EC_Earth3_Veg_ssp585_2061_2080 <- `wc2.1_5m_Bio_EC-Earth3-Veg_ssp585_2061-2080`

myExpl_EC_Earth3_Veg_ssp585_2081_2100 <- `wc2.1_5m_Bio_EC-Earth3-Veg_ssp585_2081-2100`

#MPI-ESM1-2-HR ssp245

myExpl_MPI_ESM1_2_HR_ssp245_2021_2040 <- `wc2.1_5m_Bio_MPI-ESM1-2-HR_ssp245_2021-2040`

myExpl_MPI_ESM1_2_HR_ssp245_2041_2060 <- `wc2.1_5m_Bio_MPI-ESM1-2-HR_ssp245_2041-2060`

myExpl_MPI_ESM1_2_HR_ssp245_2061_2080 <- `wc2.1_5m_Bio_MPI-ESM1-2-HR_ssp245_2061-2080`

myExpl_MPI_ESM1_2_HR_ssp245_2081_2100 <- `wc2.1_5m_Bio_MPI-ESM1-2-HR_ssp245_2081-2100`

#MPI-ESM1-2-HR ssp585

myExpl_MPI_ESM1_2_HR_ssp585_2021_2040 <- `wc2.1_5m_Bio_MPI-ESM1-2-HR_ssp585_2021-2040`

myExpl_MPI_ESM1_2_HR_ssp585_2041_2060 <- `wc2.1_5m_Bio_MPI-ESM1-2-HR_ssp585_2041-2060`

myExpl_MPI_ESM1_2_HR_ssp585_2061_2080 <- `wc2.1_5m_Bio_MPI-ESM1-2-HR_ssp585_2061-2080`

myExpl_MPI_ESM1_2_HR_ssp585_2081_2100 <- `wc2.1_5m_Bio_MPI-ESM1-2-HR_ssp585_2081-2100`

#MRI-ESM2-0 ssp245

myExpl_MRI_ESM2_0_ssp245_2021_2040 <- `wc2.1_5m_Bio_MRI-ESM2-0_ssp245_2021-2040`

myExpl_MRI_ESM2_0_ssp245_2041_2060 <- `wc2.1_5m_Bio_MRI-ESM2-0_ssp245_2041-2060`

myExpl_MRI_ESM2_0_ssp245_2061_2080 <- `wc2.1_5m_Bio_MRI-ESM2-0_ssp245_2061-2080`

myExpl_MRI_ESM2_0_ssp245_2081_2100 <- `wc2.1_5m_Bio_MRI-ESM2-0_ssp245_2081-2100`

#MRI-ESM2-0 ssp585

myExpl_MRI_ESM2_0_ssp585_2021_2040 <- `wc2.1_5m_Bio_MRI-ESM2-0_ssp585_2021-2040`

myExpl_MRI_ESM2_0_ssp585_2041_2060 <- `wc2.1_5m_Bio_MRI-ESM2-0_ssp585_2041-2060`

myExpl_MRI_ESM2_0_ssp585_2061_2080 <- `wc2.1_5m_Bio_MRI-ESM2-0_ssp585_2061-2080`

myExpl_MRI_ESM2_0_ssp585_2081_2100 <- `wc2.1_5m_Bio_MRI-ESM2-0_ssp585_2081-2100`

# BIOMOD2 Ensemble Forecasting of GCMs

#EC-Earth3-Veg ssp245

# 2021-2040

myBiomodEMProj_EC_Earth3_Veg_ssp245_2021_2040_native <- BIOMOD_EnsembleForecasting(

bm.em = myBiomodEM_native,

proj.name = 'EC_Earth3_Veg_ssp245_2021_2040_native',

new.env = myExpl_EC_Earth3_Veg_ssp245_2021_2040,

models.chosen = 'all',

metric.binary = 'all',

metric.filter = 'all')

myBiomodEMProj_EC_Earth3_Veg_ssp245_2021_2040_expanded <- BIOMOD_EnsembleForecasting(

bm.em = myBiomodEM_expanded,

proj.name = 'EC_Earth3_Veg_ssp245_2021_2040_expanded',

new.env = myExpl_EC_Earth3_Veg_ssp245_2021_2040,

models.chosen = 'all',

metric.binary = 'all',

metric.filter = 'all')

# 2041-2060

myBiomodEMProj_EC_Earth3_Veg_ssp245_2041_2060_native <- BIOMOD_EnsembleForecasting(

bm.em = myBiomodEM_native,

proj.name = 'EC_Earth3_Veg_ssp245_2041_2060_native',

new.env = myExpl_EC_Earth3_Veg_ssp245_2041_2060,

models.chosen = 'all',

metric.binary = 'all',

metric.filter = 'all')

myBiomodEMProj_EC_Earth3_Veg_ssp245_2041_2060_expanded <- BIOMOD_EnsembleForecasting(

bm.em = myBiomodEM_expanded,

proj.name = 'EC_Earth3_Veg_ssp245_2041_2060_expanded',

new.env = myExpl_EC_Earth3_Veg_ssp245_2041_2060,

models.chosen = 'all',

metric.binary = 'all',

metric.filter = 'all')

# 2061-2080

myBiomodEMProj_EC_Earth3_Veg_ssp245_2061_2080_native <- BIOMOD_EnsembleForecasting(

bm.em = myBiomodEM_native,

proj.name = 'EC_Earth3_Veg_ssp245_2061_2080_native',

new.env = myExpl_EC_Earth3_Veg_ssp245_2061_2080,

models.chosen = 'all',

metric.binary = 'all',

metric.filter = 'all')

myBiomodEMProj_EC_Earth3_Veg_ssp245_2061_2080_expanded <- BIOMOD_EnsembleForecasting(

bm.em = myBiomodEM_expanded,

proj.name = 'EC_Earth3_Veg_ssp245_2061_2080_expanded',

new.env = myExpl_EC_Earth3_Veg_ssp245_2061_2080,

models.chosen = 'all',

metric.binary = 'all',

metric.filter = 'all')

# 2081-2100

myBiomodEMProj_EC_Earth3_Veg_ssp245_2081_2100_native <- BIOMOD_EnsembleForecasting(

bm.em = myBiomodEM_native,

proj.name = 'EC_Earth3_Veg_ssp245_2081_2100_native',

new.env = myExpl_EC_Earth3_Veg_ssp245_2081_2100,

models.chosen = 'all',

metric.binary = 'all',

metric.filter = 'all')

myBiomodEMProj_EC_Earth3_Veg_ssp245_2081_2100_expanded <- BIOMOD_EnsembleForecasting(

bm.em = myBiomodEM_expanded,

proj.name = 'EC_Earth3_Veg_ssp245_2081_2100_expanded',

new.env = myExpl_EC_Earth3_Veg_ssp245_2081_2100,

models.chosen = 'all',

metric.binary = 'all',

metric.filter = 'all')

#EC-Earth3-Veg ssp585

# 2021-2040

myBiomodEMProj_EC_Earth3_Veg_ssp585_2021_2040_native <- BIOMOD_EnsembleForecasting(

bm.em = myBiomodEM_native,

proj.name = 'EC_Earth3_Veg_ssp585_2021_2040_native',

new.env = myExpl_EC_Earth3_Veg_ssp585_2021_2040,

models.chosen = 'all',

metric.binary = 'all',

metric.filter = 'all')

myBiomodEMProj_EC_Earth3_Veg_ssp585_2021_2040_expanded <- BIOMOD_EnsembleForecasting(

bm.em = myBiomodEM_expanded,

proj.name = 'EC_Earth3_Veg_ssp585_2021_2040_expanded',

new.env = myExpl_EC_Earth3_Veg_ssp585_2021_2040,

models.chosen = 'all',

metric.binary = 'all',

metric.filter = 'all')

# 2041-2060

myBiomodEMProj_EC_Earth3_Veg_ssp585_2041_2060_native <- BIOMOD_EnsembleForecasting(

bm.em = myBiomodEM_native,

proj.name = 'EC_Earth3_Veg_ssp585_2041_2060_native',

new.env = myExpl_EC_Earth3_Veg_ssp585_2041_2060,

models.chosen = 'all',

metric.binary = 'all',

metric.filter = 'all')

myBiomodEMProj_EC_Earth3_Veg_ssp585_2041_2060_expanded <- BIOMOD_EnsembleForecasting(

bm.em = myBiomodEM_expanded,

proj.name = 'EC_Earth3_Veg_ssp585_2041_2060_expanded',

new.env = myExpl_EC_Earth3_Veg_ssp585_2041_2060,

models.chosen = 'all',

metric.binary = 'all',

metric.filter = 'all')

# 2061-2080

myBiomodEMProj_EC_Earth3_Veg_ssp585_2061_2080_native <- BIOMOD_EnsembleForecasting(

bm.em = myBiomodEM_native,

proj.name = 'EC_Earth3_Veg_ssp585_2061_2080_native',

new.env = myExpl_EC_Earth3_Veg_ssp585_2061_2080,

models.chosen = 'all',

metric.binary = 'all',

metric.filter = 'all')

myBiomodEMProj_EC_Earth3_Veg_ssp585_2061_2080_expanded <- BIOMOD_EnsembleForecasting(

bm.em = myBiomodEM_expanded,

proj.name = 'EC_Earth3_Veg_ssp585_2061_2080_expanded',

new.env = myExpl_EC_Earth3_Veg_ssp585_2061_2080,

models.chosen = 'all',

metric.binary = 'all',

metric.filter = 'all')

# 2081-2100

myBiomodEMProj_EC_Earth3_Veg_ssp585_2081_2100_native <- BIOMOD_EnsembleForecasting(

bm.em = myBiomodEM_native,

proj.name = 'EC_Earth3_Veg_ssp585_2081_2100_native',

new.env = myExpl_EC_Earth3_Veg_ssp585_2081_2100,

models.chosen = 'all',

metric.binary = 'all',

metric.filter = 'all')

myBiomodEMProj_EC_Earth3_Veg_ssp585_2081_2100_expanded <- BIOMOD_EnsembleForecasting(

bm.em = myBiomodEM_expanded,

proj.name = 'EC_Earth3_Veg_ssp585_2081_2100_expanded',

new.env = myExpl_EC_Earth3_Veg_ssp585_2081_2100,

models.chosen = 'all',

metric.binary = 'all',

metric.filter = 'all')

#MPI-ESM1-2-HR ssp245

# 2021-2040

myBiomodEMProj_MPI_ESM1_2_HR_ssp245_2021_2040_native <- BIOMOD_EnsembleForecasting(

bm.em = myBiomodEM_native,

proj.name = 'MPI_ESM1_2_HR_ssp245_2021_2040_native',

new.env = myExpl_MPI_ESM1_2_HR_ssp245_2021_2040,

models.chosen = 'all',

metric.binary = 'all',

metric.filter = 'all')

myBiomodEMProj_MPI_ESM1_2_HR_ssp245_2021_2040_expanded <- BIOMOD_EnsembleForecasting(

bm.em = myBiomodEM_expanded,

proj.name = 'MPI_ESM1_2_HR_ssp245_2021_2040_expanded',

new.env = myExpl_MPI_ESM1_2_HR_ssp245_2021_2040,

models.chosen = 'all',

metric.binary = 'all',

metric.filter = 'all')

# 2041-2060

myBiomodEMProj_MPI_ESM1_2_HR_ssp245_2041_2060_native <- BIOMOD_EnsembleForecasting(

bm.em = myBiomodEM_native,

proj.name = 'MPI_ESM1_2_HR_ssp245_2041_2060_native',

new.env = myExpl_MPI_ESM1_2_HR_ssp245_2041_2060,

models.chosen = 'all',

metric.binary = 'all',

metric.filter = 'all')

myBiomodEMProj_MPI_ESM1_2_HR_ssp245_2041_2060_expanded <- BIOMOD_EnsembleForecasting(

bm.em = myBiomodEM_expanded,

proj.name = 'MPI_ESM1_2_HR_ssp245_2041_2060_expanded',

new.env = myExpl_MPI_ESM1_2_HR_ssp245_2041_2060,

models.chosen = 'all',

metric.binary = 'all',

metric.filter = 'all')

# 2061-2080

myBiomodEMProj_MPI_ESM1_2_HR_ssp245_2061_2080_native <- BIOMOD_EnsembleForecasting(

bm.em = myBiomodEM_native,

proj.name = 'MPI_ESM1_2_HR_ssp245_2061_2080_native',

new.env = myExpl_MPI_ESM1_2_HR_ssp245_2061_2080,

models.chosen = 'all',

metric.binary = 'all',

metric.filter = 'all')

myBiomodEMProj_MPI_ESM1_2_HR_ssp245_2061_2080_expanded <- BIOMOD_EnsembleForecasting(

bm.em = myBiomodEM_expanded,

proj.name = 'MPI_ESM1_2_HR_ssp245_2061_2080_expanded',

new.env = myExpl_MPI_ESM1_2_HR_ssp245_2061_2080,

models.chosen = 'all',

metric.binary = 'all',

metric.filter = 'all')

# 2081-2100

myBiomodEMProj_MPI_ESM1_2_HR_ssp245_2081_2100_native <- BIOMOD_EnsembleForecasting(

bm.em = myBiomodEM_native,

proj.name = 'MPI_ESM1_2_HR_ssp245_2081_2100_native',

new.env = myExpl_MPI_ESM1_2_HR_ssp245_2081_2100,

models.chosen = 'all',

metric.binary = 'all',

metric.filter = 'all')

myBiomodEMProj_MPI_ESM1_2_HR_ssp245_2081_2100_expanded <- BIOMOD_EnsembleForecasting(

bm.em = myBiomodEM_expanded,

proj.name = 'MPI_ESM1_2_HR_ssp245_2081_2100_expanded',

new.env = myExpl_MPI_ESM1_2_HR_ssp245_2081_2100,

models.chosen = 'all',

metric.binary = 'all',

metric.filter = 'all')

#MPI-ESM1-2-HR ssp585

# 2021-2040

myBiomodEMProj_MPI_ESM1_2_HR_ssp585_2021_2040_native <- BIOMOD_EnsembleForecasting(

bm.em = myBiomodEM_native,

proj.name = 'MPI_ESM1_2_HR_ssp585_2021_2040_native',

new.env = myExpl_MPI_ESM1_2_HR_ssp585_2021_2040,

models.chosen = 'all',

metric.binary = 'all',

metric.filter = 'all')

myBiomodEMProj_MPI_ESM1_2_HR_ssp585_2021_2040_expanded <- BIOMOD_EnsembleForecasting(

bm.em = myBiomodEM_expanded,

proj.name = 'MPI_ESM1_2_HR_ssp585_2021_2040_expanded',

new.env = myExpl_MPI_ESM1_2_HR_ssp585_2021_2040,

models.chosen = 'all',

metric.binary = 'all',

metric.filter = 'all')

# 2041-2060

myBiomodEMProj_MPI_ESM1_2_HR_ssp585_2041_2060_native <- BIOMOD_EnsembleForecasting(

bm.em = myBiomodEM_native,

proj.name = 'MPI_ESM1_2_HR_ssp585_2041_2060_native',

new.env = myExpl_MPI_ESM1_2_HR_ssp585_2041_2060,

models.chosen = 'all',

metric.binary = 'all',

metric.filter = 'all')

myBiomodEMProj_MPI_ESM1_2_HR_ssp585_2041_2060_expanded <- BIOMOD_EnsembleForecasting(

bm.em = myBiomodEM_expanded,

proj.name = 'MPI_ESM1_2_HR_ssp585_2041_2060_expanded',

new.env = myExpl_MPI_ESM1_2_HR_ssp585_2041_2060,

models.chosen = 'all',

metric.binary = 'all',

metric.filter = 'all')

# 2061-2080

myBiomodEMProj_MPI_ESM1_2_HR_ssp585_2061_2080_native <- BIOMOD_EnsembleForecasting(

bm.em = myBiomodEM_native,

proj.name = 'MPI_ESM1_2_HR_ssp585_2061_2080_native',

new.env = myExpl_MPI_ESM1_2_HR_ssp585_2061_2080,

models.chosen = 'all',

metric.binary = 'all',

metric.filter = 'all')

myBiomodEMProj_MPI_ESM1_2_HR_ssp585_2061_2080_expanded <- BIOMOD_EnsembleForecasting(

bm.em = myBiomodEM_expanded,

proj.name = 'MPI_ESM1_2_HR_ssp585_2061_2080_expanded',

new.env = myExpl_MPI_ESM1_2_HR_ssp585_2061_2080,

models.chosen = 'all',

metric.binary = 'all',

metric.filter = 'all')

# 2081-2100

myBiomodEMProj_MPI_ESM1_2_HR_ssp585_2081_2100_native <- BIOMOD_EnsembleForecasting(

bm.em = myBiomodEM_native,

proj.name = 'MPI_ESM1_2_HR_ssp585_2081_2100_native',

new.env = myExpl_MPI_ESM1_2_HR_ssp585_2081_2100,

models.chosen = 'all',

metric.binary = 'all',

metric.filter = 'all')

myBiomodEMProj_MPI_ESM1_2_HR_ssp585_2081_2100_expanded <- BIOMOD_EnsembleForecasting(

bm.em = myBiomodEM_expanded,

proj.name = 'MPI_ESM1_2_HR_ssp585_2081_2100_expanded',

new.env = myExpl_MPI_ESM1_2_HR_ssp585_2081_2100,

models.chosen = 'all',

metric.binary = 'all',

metric.filter = 'all')

#MRI-ESM2-0 ssp245

# 2021-2040

myBiomodEMProj_MRI_ESM2_0_ssp245_2021_2040_native <- BIOMOD_EnsembleForecasting(

bm.em = myBiomodEM_native,

proj.name = 'MRI_ESM2_0_ssp245_2021_2040_native',

new.env = myExpl_MRI_ESM2_0_ssp245_2021_2040,

models.chosen = 'all',

metric.binary = 'all',

metric.filter = 'all')

myBiomodEMProj_MRI_ESM2_0_ssp245_2021_2040_expanded <- BIOMOD_EnsembleForecasting(

bm.em = myBiomodEM_expanded,

proj.name = 'MRI_ESM2_0_ssp245_2021_2040_expanded',

new.env = myExpl_MRI_ESM2_0_ssp245_2021_2040,

models.chosen = 'all',

metric.binary = 'all',

metric.filter = 'all')

# 2041-2060

myBiomodEMProj_MRI_ESM2_0_ssp245_2041_2060_native <- BIOMOD_EnsembleForecasting(

bm.em = myBiomodEM_native,

proj.name = 'MRI_ESM2_0_ssp245_2041_2060_native',

new.env = myExpl_MRI_ESM2_0_ssp245_2041_2060,

models.chosen = 'all',

metric.binary = 'all',

metric.filter = 'all')

myBiomodEMProj_MRI_ESM2_0_ssp245_2041_2060_expanded <- BIOMOD_EnsembleForecasting(

bm.em = myBiomodEM_expanded,

proj.name = 'MRI_ESM2_0_ssp245_2041_2060_expanded',

new.env = myExpl_MRI_ESM2_0_ssp245_2041_2060,

models.chosen = 'all',

metric.binary = 'all',

metric.filter = 'all')

# 2061-2080

myBiomodEMProj_MRI_ESM2_0_ssp245_2061_2080_native <- BIOMOD_EnsembleForecasting(

bm.em = myBiomodEM_native,

proj.name = 'MRI_ESM2_0_ssp245_2061_2080_native',

new.env = myExpl_MRI_ESM2_0_ssp245_2061_2080,

models.chosen = 'all',

metric.binary = 'all',

metric.filter = 'all')

myBiomodEMProj_MRI_ESM2_0_ssp245_2061_2080_expanded <- BIOMOD_EnsembleForecasting(

bm.em = myBiomodEM_expanded,

proj.name = 'MRI_ESM2_0_ssp245_2061_2080_expanded',

new.env = myExpl_MRI_ESM2_0_ssp245_2061_2080,

models.chosen = 'all',

metric.binary = 'all',

metric.filter = 'all')

# 2081-2100

myBiomodEMProj_MRI_ESM2_0_ssp245_2081_2100_native <- BIOMOD_EnsembleForecasting(

bm.em = myBiomodEM_native,

proj.name = 'MRI_ESM2_0_ssp245_2081_2100_native',

new.env = myExpl_MRI_ESM2_0_ssp245_2081_2100,

models.chosen = 'all',

metric.binary = 'all',

metric.filter = 'all')

myBiomodEMProj_MRI_ESM2_0_ssp245_2081_2100_expanded <- BIOMOD_EnsembleForecasting(

bm.em = myBiomodEM_expanded,

proj.name = 'MRI_ESM2_0_ssp245_2081_2100_expanded',

new.env = myExpl_MRI_ESM2_0_ssp245_2081_2100,

models.chosen = 'all',

metric.binary = 'all',

metric.filter = 'all')

#MRI-ESM2-0 ssp585

# 2021-2040

myBiomodEMProj_MRI_ESM2_0_ssp585_2021_2040_native <- BIOMOD_EnsembleForecasting(

bm.em = myBiomodEM_native,

proj.name = 'MRI_ESM2_0_ssp585_2021_2040_native',

new.env = myExpl_MRI_ESM2_0_ssp585_2021_2040,

models.chosen = 'all',

metric.binary = 'all',

metric.filter = 'all')

myBiomodEMProj_MRI_ESM2_0_ssp585_2021_2040_expanded <- BIOMOD_EnsembleForecasting(

bm.em = myBiomodEM_expanded,

proj.name = 'MRI_ESM2_0_ssp585_2021_2040_expanded',

new.env = myExpl_MRI_ESM2_0_ssp585_2021_2040,

models.chosen = 'all',

metric.binary = 'all',

metric.filter = 'all')

# 2041-2060

myBiomodEMProj_MRI_ESM2_0_ssp585_2041_2060_native <- BIOMOD_EnsembleForecasting(

bm.em = myBiomodEM_native,

proj.name = 'MRI_ESM2_0_ssp585_2041_2060_native',

new.env = myExpl_MRI_ESM2_0_ssp585_2041_2060,

models.chosen = 'all',

metric.binary = 'all',

metric.filter = 'all')

myBiomodEMProj_MRI_ESM2_0_ssp585_2041_2060_expanded <- BIOMOD_EnsembleForecasting(

bm.em = myBiomodEM_expanded,

proj.name = 'MRI_ESM2_0_ssp585_2041_2060_expanded',

new.env = myExpl_MRI_ESM2_0_ssp585_2041_2060,

models.chosen = 'all',

metric.binary = 'all',

metric.filter = 'all')

# 2061-2080

myBiomodEMProj_MRI_ESM2_0_ssp585_2061_2080_native <- BIOMOD_EnsembleForecasting(

bm.em = myBiomodEM_native,

proj.name = 'MRI_ESM2_0_ssp585_2061_2080_native',

new.env = myExpl_MRI_ESM2_0_ssp585_2061_2080,

models.chosen = 'all',

metric.binary = 'all',

metric.filter = 'all')

myBiomodEMProj_MRI_ESM2_0_ssp585_2061_2080_expanded <- BIOMOD_EnsembleForecasting(

bm.em = myBiomodEM_expanded,

proj.name = 'MRI_ESM2_0_ssp585_2061_2080_expanded',

new.env = myExpl_MRI_ESM2_0_ssp585_2061_2080,

models.chosen = 'all',

metric.binary = 'all',

metric.filter = 'all')

# 2081-2100

myBiomodEMProj_MRI_ESM2_0_ssp585_2081_2100_native <- BIOMOD_EnsembleForecasting(

bm.em = myBiomodEM_native,

proj.name = 'MRI_ESM2_0_ssp585_2081_2100_native',

new.env = myExpl_MRI_ESM2_0_ssp585_2081_2100,

models.chosen = 'all',

metric.binary = 'all',

metric.filter = 'all')

myBiomodEMProj_MRI_ESM2_0_ssp585_2081_2100_expanded <- BIOMOD_EnsembleForecasting(

bm.em = myBiomodEM_expanded,

proj.name = 'MRI_ESM2_0_ssp585_2081_2100_expanded',

new.env = myExpl_MRI_ESM2_0_ssp585_2081_2100,

models.chosen = 'all',

metric.binary = 'all',

metric.filter = 'all')

#Mapping of GCM projections for supplementary materials

library(terra)

library(tmap)

library(stringr)

file_paths <- c(

"D:/Niche modelling/CSV range/Diff_By_Pixel_EC_Earth3_Veg_ssp585_2081_2100_expanded.tif",

"D:/Niche modelling/CSV range/Diff_By_Pixel_EC_Earth3_Veg_ssp585_2081_2100_native.tif",

"D:/Niche modelling/CSV range/Diff_By_Pixel_MPI_ESM1_2_HR_ssp245_2021_2040_expanded.tif",

"D:/Niche modelling/CSV range/Diff_By_Pixel_MPI_ESM1_2_HR_ssp245_2021_2040_native.tif",

"D:/Niche modelling/CSV range/Diff_By_Pixel_MPI_ESM1_2_HR_ssp245_2041_2060_expanded.tif",

"D:/Niche modelling/CSV range/Diff_By_Pixel_MPI_ESM1_2_HR_ssp245_2041_2060_native.tif",

"D:/Niche modelling/CSV range/Diff_By_Pixel_MPI_ESM1_2_HR_ssp245_2061_2080_expanded.tif",

"D:/Niche modelling/CSV range/Diff_By_Pixel_MPI_ESM1_2_HR_ssp245_2061_2080_native.tif",

"D:/Niche modelling/CSV range/Diff_By_Pixel_MPI_ESM1_2_HR_ssp245_2081_2100_expanded.tif",

"D:/Niche modelling/CSV range/Diff_By_Pixel_MPI_ESM1_2_HR_ssp245_2081_2100_native.tif",

"D:/Niche modelling/CSV range/Diff_By_Pixel_MPI_ESM1_2_HR_ssp585_2021_2040_expanded.tif",

"D:/Niche modelling/CSV range/Diff_By_Pixel_MPI_ESM1_2_HR_ssp585_2021_2040_native.tif",

"D:/Niche modelling/CSV range/Diff_By_Pixel_MPI_ESM1_2_HR_ssp585_2041_2060_expanded.tif",

"D:/Niche modelling/CSV range/Diff_By_Pixel_MPI_ESM1_2_HR_ssp585_2041_2060_native.tif",

"D:/Niche modelling/CSV range/Diff_By_Pixel_MPI_ESM1_2_HR_ssp585_2061_2080_expanded.tif",

"D:/Niche modelling/CSV range/Diff_By_Pixel_MPI_ESM1_2_HR_ssp585_2061_2080_native.tif",

"D:/Niche modelling/CSV range/Diff_By_Pixel_MPI_ESM1_2_HR_ssp585_2081_2100_expanded.tif",

"D:/Niche modelling/CSV range/Diff_By_Pixel_MPI_ESM1_2_HR_ssp585_2081_2100_native.tif",

"D:/Niche modelling/CSV range/Diff_By_Pixel_MRI_ESM2_0_ssp245_2021_2040_expanded.tif",

"D:/Niche modelling/CSV range/Diff_By_Pixel_MRI_ESM2_0_ssp245_2021_2040_native.tif",

"D:/Niche modelling/CSV range/Diff_By_Pixel_MRI_ESM2_0_ssp245_2041_2060_expanded.tif",

"D:/Niche modelling/CSV range/Diff_By_Pixel_MRI_ESM2_0_ssp245_2041_2060_native.tif",

"D:/Niche modelling/CSV range/Diff_By_Pixel_MRI_ESM2_0_ssp245_2061_2080_expanded.tif",

"D:/Niche modelling/CSV range/Diff_By_Pixel_MRI_ESM2_0_ssp245_2061_2080_native.tif",

"D:/Niche modelling/CSV range/Diff_By_Pixel_MRI_ESM2_0_ssp245_2081_2100_expanded.tif",

"D:/Niche modelling/CSV range/Diff_By_Pixel_MRI_ESM2_0_ssp245_2081_2100_native.tif",

"D:/Niche modelling/CSV range/Diff_By_Pixel_MRI_ESM2_0_ssp585_2021_2040_expanded.tif",

"D:/Niche modelling/CSV range/Diff_By_Pixel_MRI_ESM2_0_ssp585_2021_2040_native.tif",

"D:/Niche modelling/CSV range/Diff_By_Pixel_MRI_ESM2_0_ssp585_2041_2060_expanded.tif",

"D:/Niche modelling/CSV range/Diff_By_Pixel_MRI_ESM2_0_ssp585_2041_2060_native.tif",

"D:/Niche modelling/CSV range/Diff_By_Pixel_MRI_ESM2_0_ssp585_2061_2080_expanded.tif",

"D:/Niche modelling/CSV range/Diff_By_Pixel_MRI_ESM2_0_ssp585_2061_2080_native.tif",

"D:/Niche modelling/CSV range/Diff_By_Pixel_MRI_ESM2_0_ssp585_2081_2100_expanded.tif",

"D:/Niche modelling/CSV range/Diff_By_Pixel_MRI_ESM2_0_ssp585_2081_2100_native.tif",

"D:/Niche modelling/CSV range/Diff_By_Pixel_EC_Earth3_Veg_ssp245_2021_2040_expanded.tif",

"D:/Niche modelling/CSV range/Diff_By_Pixel_EC_Earth3_Veg_ssp245_2021_2040_native.tif",

"D:/Niche modelling/CSV range/Diff_By_Pixel_EC_Earth3_Veg_ssp245_2041_2060_expanded.tif",

"D:/Niche modelling/CSV range/Diff_By_Pixel_EC_Earth3_Veg_ssp245_2041_2060_native.tif",

"D:/Niche modelling/CSV range/Diff_By_Pixel_EC_Earth3_Veg_ssp245_2061_2080_expanded.tif",

"D:/Niche modelling/CSV range/Diff_By_Pixel_EC_Earth3_Veg_ssp245_2061_2080_native.tif",

"D:/Niche modelling/CSV range/Diff_By_Pixel_EC_Earth3_Veg_ssp245_2081_2100_expanded.tif",

"D:/Niche modelling/CSV range/Diff_By_Pixel_EC_Earth3_Veg_ssp245_2081_2100_native.tif",

"D:/Niche modelling/CSV range/Diff_By_Pixel_EC_Earth3_Veg_ssp585_2021_2040_expanded.tif",

"D:/Niche modelling/CSV range/Diff_By_Pixel_EC_Earth3_Veg_ssp585_2021_2040_native.tif",

"D:/Niche modelling/CSV range/Diff_By_Pixel_EC_Earth3_Veg_ssp585_2041_2060_expanded.tif",

"D:/Niche modelling/CSV range/Diff_By_Pixel_EC_Earth3_Veg_ssp585_2041_2060_native.tif",

"D:/Niche modelling/CSV range/Diff_By_Pixel_EC_Earth3_Veg_ssp585_2061_2080_expanded.tif",

"D:/Niche modelling/CSV range/Diff_By_Pixel_EC_Earth3_Veg_ssp585_2061_2080_native.tif"

)

#Color palette and labels

value_colors <- c(

"-2" = "#e31a1c",#Red

"-1" = "#fdbf6f",#Orange

"0"= "#b2df8a",#Green

"1"= "#1f78b4" #Blue

)

value_labels <- c(

"-2" = "Predicted to be lost",

"-1" = "Predicted to remain occupied",

"0"= "Predicted to remain unoccupied",

"1"= "Predicted to be gained"

)

#Loop through files

for (file in file_paths) {

cat("Processing:", file, "\n")

filename <- basename(file)

match <- str_match(filename, "Diff_By_Pixel_([A-Za-z0-9_]+)_ssp(\\d{3})_(\\d{4})_(\\d{4})_(expanded|native)\\.tif")

if (!is.na(match[1,1])) {

model <- gsub("_", "-", match[1,2])

scenario <- paste0("ssp", match[1,3])

start_year <- match[1,4]

end_year <- match[1,5]

data_type <- match[1,6]

map_title <- paste(model, scenario, paste0("(", start_year, "–", end_year, ") – Habitat Change"))

} else {

map_title <- gsub(".tif$", "", filename)

}

r <- rast(file)

#Make the map

map <- tm_shape(r) +

tm_raster(

style = "cat",

palette = value_colors,

labels = value_labels,

title = "Habitat Change"

) +

tm_scale_bar(position = c("left", "bottom"), text.size = 0.6) +

tm_layout(

main.title = map_title,

main.title.size = 1.4,

legend.outside = TRUE,

legend.outside.position = "right",

legend.title.size = 0.8,

legend.text.size = 0.6,

outer.margin = 0,

frame = FALSE

)

output_file <- gsub(".tif$", "_map.png", file)

tmap_save(

tm = map,

filename = output_file,

width = 12,

height = 7,

units = "in",

dpi = 300

)

}

#Extract Range Change Metrics from Existing BIOMOD_RangeSize Results

#Function to extract metrics from an existing range change object

extract_existing_metrics <- function(range_size_obj, model_name, scenario, period, distribution_type) {

#Extract metrics from Compt.By.Models

metrics_df <- as.data.frame(range_size_obj$Compt.By.Models)

#Add row names

metrics_df$eval_method <- rownames(metrics_df)

rownames(metrics_df) <- NULL

#Identify which evaluation method each row corresponds to

metrics_df$metric <- NA

metrics_df$metric[grep("TSS", metrics_df$eval_method)] <- "TSS"

#columns

metrics_df$model <- model_name

metrics_df$scenario <- scenario

metrics_df$period <- period

metrics_df$distribution <- distribution_type

metrics_df <- metrics_df[, c("model", "scenario", "period", "distribution", "metric", "eval_method",

colnames(metrics_df)[!(colnames(metrics_df) %in%

c("model", "scenario", "period",

"distribution", "metric", "eval_method"))])]

return(metrics_df)

}

#Initialize empty dataframe to store all results

all_metrics <- data.frame()

#Extract metrics from existing native distribution range size objects

#EC_EARTH3 SSP245 - Native

all_metrics <- rbind(all_metrics,

extract_existing_metrics(native_EC_EARTH3_ssp245_2021_2040,

"EC_EARTH3", "SSP245", "2021-2040", "native"))

all_metrics <- rbind(all_metrics,

extract_existing_metrics(native_EC_EARTH3_ssp245_2041_2060,

"EC_EARTH3", "SSP245", "2041-2060", "native"))

all_metrics <- rbind(all_metrics,

extract_existing_metrics(native_EC_EARTH3_ssp245_2061_2080,

"EC_EARTH3", "SSP245", "2061-2080", "native"))

all_metrics <- rbind(all_metrics,

extract_existing_metrics(native_EC_EARTH3_ssp245_2081_2100,

"EC_EARTH3", "SSP245", "2081-2100", "native"))

#EC_EARTH3 SSP585 - Native

all_metrics <- rbind(all_metrics,

extract_existing_metrics(native_EC_EARTH3_ssp585_2021_2040,

"EC_EARTH3", "SSP585", "2021-2040", "native"))

all_metrics <- rbind(all_metrics,

extract_existing_metrics(native_EC_EARTH3_ssp585_2041_2060,

"EC_EARTH3", "SSP585", "2041-2060", "native"))

all_metrics <- rbind(all_metrics,

extract_existing_metrics(native_EC_EARTH3_ssp585_2061_2080,

"EC_EARTH3", "SSP585", "2061-2080", "native"))

all_metrics <- rbind(all_metrics,

extract_existing_metrics(native_EC_EARTH3_ssp585_2081_2100,

"EC_EARTH3", "SSP585", "2081-2100", "native"))

#MRI_ESM2_0 SSP245 - Native

all_metrics <- rbind(all_metrics,

extract_existing_metrics(native_MRI_ESM2_0_ssp245_2021_2040,

"MRI_ESM2_0", "SSP245", "2021-2040", "native"))

all_metrics <- rbind(all_metrics,

extract_existing_metrics(native_MRI_ESM2_0_ssp245_2041_2060,

"MRI_ESM2_0", "SSP245", "2041-2060", "native"))

all_metrics <- rbind(all_metrics,

extract_existing_metrics(native_MRI_ESM2_0_ssp245_2061_2080,

"MRI_ESM2_0", "SSP245", "2061-2080", "native"))

all_metrics <- rbind(all_metrics,

extract_existing_metrics(native_MRI_ESM2_0_ssp245_2081_2100,

"MRI_ESM2_0", "SSP245", "2081-2100", "native"))

#MRI_ESM2_0 SSP585 - Native

all_metrics <- rbind(all_metrics,

extract_existing_metrics(native_MRI_ESM2_0_ssp585_2021_2040,

"MRI_ESM2_0", "SSP585", "2021-2040", "native"))

all_metrics <- rbind(all_metrics,

extract_existing_metrics(native_MRI_ESM2_0_ssp585_2041_2060,

"MRI_ESM2_0", "SSP585", "2041-2060", "native"))

all_metrics <- rbind(all_metrics,

extract_existing_metrics(native_MRI_ESM2_0_ssp585_2061_2080,

"MRI_ESM2_0", "SSP585", "2061-2080", "native"))

all_metrics <- rbind(all_metrics,

extract_existing_metrics(native_MRI_ESM2_0_ssp585_2081_2100,

"MRI_ESM2_0", "SSP585", "2081-2100", "native"))

#MPI_ESM1_2_HR SSP245 - Native

all_metrics <- rbind(all_metrics,

extract_existing_metrics(native_MPI_ESM1_2_HR_ssp245_2021_2040,

"MPI_ESM1_2_HR", "SSP245", "2021-2040", "native"))

all_metrics <- rbind(all_metrics,

extract_existing_metrics(native_MPI_ESM1_2_HR_ssp245_2041_2060,

"MPI_ESM1_2_HR", "SSP245", "2041-2060", "native"))

all_metrics <- rbind(all_metrics,

extract_existing_metrics(native_MPI_ESM1_2_HR_ssp245_2061_2080,

"MPI_ESM1_2_HR", "SSP245", "2061-2080", "native"))

all_metrics <- rbind(all_metrics,

extract_existing_metrics(native_MPI_ESM1_2_HR_ssp245_2081_2100,

"MPI_ESM1_2_HR", "SSP245", "2081-2100", "native"))

#MPI_ESM1_2_HR SSP585 - Native

all_metrics <- rbind(all_metrics,

extract_existing_metrics(native_MPI_ESM1_2_HR_ssp585_2021_2040,

"MPI_ESM1_2_HR", "SSP585", "2021-2040", "native"))

all_metrics <- rbind(all_metrics,

extract_existing_metrics(native_MPI_ESM1_2_HR_ssp585_2041_2060,

"MPI_ESM1_2_HR", "SSP585", "2041-2060", "native"))

all_metrics <- rbind(all_metrics,

extract_existing_metrics(native_MPI_ESM1_2_HR_ssp585_2061_2080,

"MPI_ESM1_2_HR", "SSP585", "2061-2080", "native"))

all_metrics <- rbind(all_metrics,

extract_existing_metrics(native_MPI_ESM1_2_HR_ssp585_2081_2100,

"MPI_ESM1_2_HR", "SSP585", "2081-2100", "native"))

#Extract metrics from existing expanded distribution range size objects

#EC_EARTH3 SSP245 - Expanded

all_metrics <- rbind(all_metrics,

extract_existing_metrics(expanded_EC_EARTH3_ssp245_2021_2040,

"EC_EARTH3", "SSP245", "2021-2040", "expanded"))

all_metrics <- rbind(all_metrics,

extract_existing_metrics(expanded_EC_EARTH3_ssp245_2041_2060,

"EC_EARTH3", "SSP245", "2041-2060", "expanded"))

all_metrics <- rbind(all_metrics,

extract_existing_metrics(expanded_EC_EARTH3_ssp245_2061_2080,

"EC_EARTH3", "SSP245", "2061-2080", "expanded"))

all_metrics <- rbind(all_metrics,

extract_existing_metrics(expanded_EC_EARTH3_ssp245_2081_2100,

"EC_EARTH3", "SSP245", "2081-2100", "expanded"))

#EC_EARTH3 SSP585 - Expanded

all_metrics <- rbind(all_metrics,

extract_existing_metrics(expanded_EC_EARTH3_ssp585_2021_2040,

"EC_EARTH3", "SSP585", "2021-2040", "expanded"))

all_metrics <- rbind(all_metrics,

extract_existing_metrics(expanded_EC_EARTH3_ssp585_2041_2060,

"EC_EARTH3", "SSP585", "2041-2060", "expanded"))

all_metrics <- rbind(all_metrics,

extract_existing_metrics(expanded_EC_EARTH3_ssp585_2061_2080,

"EC_EARTH3", "SSP585", "2061-2080", "expanded"))

all_metrics <- rbind(all_metrics,

extract_existing_metrics(expanded_EC_EARTH3_ssp585_2081_2100,

"EC_EARTH3", "SSP585", "2081-2100", "expanded"))

#MRI_ESM2_0 SSP245 - Expanded

all_metrics <- rbind(all_metrics,

extract_existing_metrics(expanded_MRI_ESM2_0_ssp245_2021_2040,

"MRI_ESM2_0", "SSP245", "2021-2040", "expanded"))

all_metrics <- rbind(all_metrics,

extract_existing_metrics(expanded_MRI_ESM2_0_ssp245_2041_2060,

"MRI_ESM2_0", "SSP245", "2041-2060", "expanded"))

all_metrics <- rbind(all_metrics,

extract_existing_metrics(expanded_MRI_ESM2_0_ssp245_2061_2080,

"MRI_ESM2_0", "SSP245", "2061-2080", "expanded"))

all_metrics <- rbind(all_metrics,

extract_existing_metrics(expanded_MRI_ESM2_0_ssp245_2081_2100,

"MRI_ESM2_0", "SSP245", "2081-2100", "expanded"))

#MRI_ESM2_0 SSP585 - Expanded

all_metrics <- rbind(all_metrics,

extract_existing_metrics(expanded_MRI_ESM2_0_ssp585_2021_2040,

"MRI_ESM2_0", "SSP585", "2021-2040", "expanded"))

all_metrics <- rbind(all_metrics,

extract_existing_metrics(expanded_MRI_ESM2_0_ssp585_2041_2060,

"MRI_ESM2_0", "SSP585", "2041-2060", "expanded"))

all_metrics <- rbind(all_metrics,

extract_existing_metrics(expanded_MRI_ESM2_0_ssp585_2061_2080,

"MRI_ESM2_0", "SSP585", "2061-2080", "expanded"))

all_metrics <- rbind(all_metrics,

extract_existing_metrics(expanded_MRI_ESM2_0_ssp585_2081_2100,

"MRI_ESM2_0", "SSP585", "2081-2100", "expanded"))

#MPI_ESM1_2_HR SSP245 - Expanded

all_metrics <- rbind(all_metrics,

extract_existing_metrics(expanded_MPI_ESM1_2_HR_ssp245_2021_2040,

"MPI_ESM1_2_HR", "SSP245", "2021-2040", "expanded"))

all_metrics <- rbind(all_metrics,

extract_existing_metrics(expanded_MPI_ESM1_2_HR_ssp245_2041_2060,

"MPI_ESM1_2_HR", "SSP245", "2041-2060", "expanded"))

all_metrics <- rbind(all_metrics,

extract_existing_metrics(expanded_MPI_ESM1_2_HR_ssp245_2061_2080,

"MPI_ESM1_2_HR", "SSP245", "2061-2080", "expanded"))

all_metrics <- rbind(all_metrics,

extract_existing_metrics(expanded_MPI_ESM1_2_HR_ssp245_2081_2100,

"MPI_ESM1_2_HR", "SSP245", "2081-2100", "expanded"))

#MPI_ESM1_2_HR SSP585 - Expanded

all_metrics <- rbind(all_metrics,

extract_existing_metrics(expanded_MPI_ESM1_2_HR_ssp585_2021_2040,

"MPI_ESM1_2_HR", "SSP585", "2021-2040", "expanded"))

all_metrics <- rbind(all_metrics,

extract_existing_metrics(expanded_MPI_ESM1_2_HR_ssp585_2041_2060,

"MPI_ESM1_2_HR", "SSP585", "2041-2060", "expanded"))

all_metrics <- rbind(all_metrics,

extract_existing_metrics(expanded_MPI_ESM1_2_HR_ssp585_2061_2080,

"MPI_ESM1_2_HR", "SSP585", "2061-2080", "expanded"))

all_metrics <- rbind(all_metrics,

extract_existing_metrics(expanded_MPI_ESM1_2_HR_ssp585_2081_2100,

"MPI_ESM1_2_HR", "SSP585", "2081-2100", "expanded"))

#Write all metrics to CSV file

write.csv(all_metrics, "Range_Change_Metrics_All_Projections.csv", row.names = FALSE)

#summary

cat("Range change metrics for all projections extracted and saved to 'Range_Change_Metrics_All_Projections.csv'\n")

cat("Total number of projections analyzed:", nrow(all_metrics), "\n")

cat("Metrics included:", paste(colnames(all_metrics)[-(1:6)], collapse=", "), "\n")

#Create summary dataframes for each evaluation metric

for (current_metric in unique(all_metrics$metric)) {

#Filter for current metric

metric_data <- all_metrics[all_metrics$metric == current_metric,]

if (nrow(metric_data) > 0) {

#Create a summary dataframe with means by model, scenario, period and distribution

summary_metrics <- aggregate(

metric_data[, -(1:6)],#Exclude identifier columns

by = list(

model = metric_data$model,

scenario = metric_data$scenario,

period = metric_data$period,

distribution = metric_data$distribution,

metric = metric_data$metric

),

FUN = mean

)

#Write summary to CSV

summary_filename <- paste0("Range_Change_Summary_", current_metric, ".csv")

write.csv(summary_metrics, summary_filename, row.names = FALSE)

cat("Summary metrics for", current_metric, "saved to", summary_filename, "\n")

#Create pivot table

if (requireNamespace("reshape2", quietly = TRUE)) {

library(reshape2)

#For each key range metric, create a pivot table

range_metrics <- c("Gain", "Loss", "Stable.Suitable", "Range.Change")

for (range_metric in range_metrics) {

if (range_metric %in% colnames(summary_metrics)) {

pivot_data <- dcast(

summary_metrics,

model + scenario + distribution ~ period,

value.var = range_metric

)

#Write to CSV

pivot_filename <- paste0("Pivot_", current_metric, "_", range_metric, "_by_Period.csv")

write.csv(pivot_data, pivot_filename, row.names = FALSE)

cat("Created pivot table for", current_metric, "-", range_metric, "by time period\n")

}

}

}

}

}

**#MESS Analysis of future GCM Models**

library(predicts)

library(terra)

#Occurrence points

occ_pts <- toxomerusData_expanded[, c("lon", "lat")] # adjust column names if needed

#Convert current RasterStack to SpatRaster

current <- terra::rast(bioclim_5m_world_sub)

#Extract current climate values at occurrence points

ref_points <- extract(current, occ_pts, ID = FALSE)

ref_points <- na.omit(ref_points)

#Loop through each future scenario and run MESS

output_dir <- "D:/Niche modelling/CMIP6/TIFS"

folders <- list.dirs(output_dir, recursive = FALSE)

for (folder in folders) {

scenario_name <- basename(folder)

future_stack <- get(scenario_name)

mess_result <- mess(x = future_stack,

v = ref_points,

full = TRUE)

out_path <- file.path(folder, paste0(scenario_name, "_MESS.tif"))

writeRaster(mess_result, out_path, overwrite = TRUE)

}

**#Niche Conservatism Script for *Toxomerus floralis* between its native and invaded ranges**

#Load libraries

library(ecospat)

library(terra)

library(rgbif)

library(geodata)

library(ade4)

library(raster)

library(biomod2)

library(grid)

library(gridExtra)

#Load thinned occurrence data from a CSV file

tfAll <- read.csv("toxomerusData_thinned_all.csv", sep = ",", header = TRUE)

#Convert the occurrence data to spatial vector format

TfOccs <- vect(tfAll, geom = c("decimalLongitude", "decimalLatitude"), crs = "+proj=longlat +datum=WGS84")

#Stack the bioclimatic sub selection files for world

bioclim_5m_world_sub <- stack(subset(bioclim_5m_world,

c("bio18",

"bio15",

"bio8",

"bio3",

"bio2")))

#Convert world bioclimatic stack to 'SpatRaster' format

bioclim_5m_world_spat <- rast(bioclim_5m_world_sub)

#Thin occurrences based on world bioclimatic data

tfOccs_thinned <- spatSample(TfOccs, size = 1, strata = bioclim_5m_world_spat)

tfOccs_thinned <- cbind(tfOccs_thinned, extract(bioclim_5m_world_spat, tfOccs_thinned))

tfOccs_thinned <- tfOccs_thinned[complete.cases(data.frame(tfOccs_thinned)), ]

#Convert regional bioclimatic stacks to 'SpatRaster' format

bioclim_5m_native_spat <- rast(bioclim_5m_native_sub)

bioclim_5m_invaded_spat <- rast(bioclim_5m_invaded_sub)

#Define extents for Native and Invaded range

native_extent <- ext(bioclim_5m_native_spat)

invaded_extent <- ext(bioclim_5m_invaded_spat)

#Crop thinned occurrences to regional extents

tfnative <- crop(tfOccs_thinned, native_extent)

tfinvaded <- crop(tfOccs_thinned, invaded_extent)

#Extract bioclimatic values for analysis

nativeEnvR <- bioclim_5m_native_spat

invadedEnvR <- bioclim_5m_invaded_spat

globalEnvR <- bioclim_5m_world_spat

#Convert bioclimatic rasters to matrices

nativeEnvM <- values(nativeEnvR)

invadedEnvM <- values(invadedEnvR)

globalEnvM <- values(globalEnvR)

#Remove rows with missing values

nativeEnvM <- nativeEnvM[complete.cases(nativeEnvM), ]

invadedEnvM <- invadedEnvM[complete.cases(invadedEnvM), ]

globalEnvM <- globalEnvM[complete.cases(globalEnvM), ]

#global environmental matrix PCA

pca.clim <- dudi.pca(globalEnvM, center = TRUE, scale = TRUE, scannf = FALSE, nf = 2)

global.scores <- pca.clim$li

#Project occurrence data into PCA space

nativeTf.scores <- suprow(pca.clim, data.frame(tfnative)[, colnames(globalEnvM)])$li

invasiveTf.scores <- suprow(pca.clim, data.frame(tfinvaded)[, colnames(globalEnvM)])$li

#Project environmental data into PCA space

nativeEnv.scores <- suprow(pca.clim, nativeEnvM)$li

invasiveEnv.scores <- suprow(pca.clim, invadedEnvM)$li

#Variable loadings (coordinates of variables on axes)

pca.clim$co

# eigenvalues

pca.clim$eig

#percentage of variance calculation

pca.clim$eig / sum(pca.cal$eig) * 100

#CorrelationCircle

s.corcircle(pca.clim$co, xax = 1, yax = 2

#Create climatic niche grids

nativeGrid <- ecospat.grid.clim.dyn(global.scores, nativeEnv.scores, nativeTf.scores)

invasiveGrid <- ecospat.grid.clim.dyn(global.scores, invasiveEnv.scores, invasiveTf.scores)

ecospat.plot.niche.dyn(nativeGrid, invasiveGrid, quant = 0.05)

indexVals <- ecospat.niche.dyn.index(nativeGrid, invasiveGrid)

indexVals$dynamic.index.w

#Project niche dynamics to geographic space

geoProj <- ecospat.niche.dynIndexProjGeo(nativeGrid, invasiveGrid, env = globalEnvR)

plot(geoProj, legend = FALSE, col = c("grey", "green", "red", "blue"), axes = FALSE, mar = c(0, 0, 0, 0))

points(tfinvaded, col = 'red', cex = 1, pch = 23, bg = "white")

points(tfnative, col = 'green', cex = 1, pch = 23, bg = "white")

par(mar = c(0,0,0,0))

box()

#Perform niche equivalency test

eq.test <- ecospat.niche.equivalency.test(nativeGrid, invasiveGrid, overlap.alternative = "higher", expansion.alternative = "lower", stability.alternative = "higher", unfilling.alternative = "lower", rep = 1000, ncores = 2)

#Extract model scores

#Getting scores from traditional models for native niche

model_scores_native <- get_evaluations(myBiomodModelOut_native)

write.csv(model_scores_native, "retained_models_native_scores.csv", row.names = TRUE)

#Getting scores from traditional models for expanded niche

model_scores_native <- get_evaluations(myBiomodModelOut_expanded)

write.csv(model_scores_native, "retained_models_expanded_scores.csv", row.names = TRUE)

#Extract retained models used to create nativeEM

retained_models_EM_native <- get_kept_models(myBiomodEM_native)

write.csv(retained_models_EM_native, "retained_models_EM_native.csv", row.names = TRUE)

#Extract retained models used to create expandedEM

retained_models_expandedEM <- get_kept_models(myBiomodEM_expanded)

write.csv(retained_models_expandedEM, "retained_models_expandedEM.csv", row.names = TRUE)
