# Supplementary material for: Invasion Patterns and Niche Dynamics of the Pollinivorous Florida Calligrapher, Toxomerus floralis (Diptera: Syrphidae) in the Afrotropical Region
Source: Ecol Evol. 2026 Jun 23;16(6):e73838. doi: 10.1002/ece3.73838 (PMC13288174; doi:10.1002/ece3.73838)
Supplement: Supplementary file 27 — Data S16: OD‐map Protocol for niche modelling of T. floralis. [file ECE3-16-e73838-s017.docx]

Invasion patterns and niche dynamics of the pollinivorous Florida calligrapher, *Toxomerus floralis* (Diptera: Syrphidae) in the Afrotropical Region

– ODMAP Protocol –

Burgert Muller; John Midgley; Georg Goergen; Ali Al Jahdhami; Michelson Azo’o Ela; Terence Bellingan; Simon Cavaillès; Robert Copeland; Marc De Meyer; Martin Hauser; Allen Holmes; Ximo Mengual; Gabriel Nève; Menno Reemer; Jeff Skevington; Gunilla Ståhls; Eugène Sinzinkayo; John Smit; Axel Ssymank; Genevieve Theron; Kurt Jordaens

2026-05-02

## Overview

#### Authorship

*Contact:* burgert.muller@nasmus.co.za

*DOI:* TBC

#### Model objective

*Model objective:* Forecast and transfer

*Target output:* probability of occurrence maps; binary presence/absence predictions; future distribution projections (2021–2100); niche overlap and expansion quantification.

#### Focal Taxon

*Focal Taxon:* *Toxomerus floralis* (Fabricius, 1798)

#### Location

*Location:* Native range: Americas (southern Canada to southern Chile and Argentina); Invaded range: Afrotropical Region (25 countries); Projections: Eastern Palaearctic, Indomalayan, and Australasian Regions

#### Scale of Analysis

*Spatial extent:* Global, with focus on native range (Americas), invaded range (Afrotropical Region), and potential expansion areas (Eastern Palaearctic, Indomalayan, Australasian Regions)

*Temporal extent:* Species occurrences: 2013-2025 (invaded range), historical to 2025 (native range); Climate data: 1970–2000 (current baseline); Future projections: 2021–2100

*Boundary:* Global extent defined by WorldClim 2.1 climate data coverage; species range boundaries defined by biogeographic regions

#### Biodiversity data

*Observation type:* Presence-only data from museum specimens, field sampling, and citizen science platforms

*Response data type:* presence/pseudo-absence data

#### Predictors

*Predictor types:* Climatic variables only (bioclimatic variables from WorldClim 2.1)

#### Hypotheses

*Hypotheses:* Climate matching hypothesis: areas with similar environmental conditions to native range are more likely to be colonized. Niche conservatism expected with a potential for niche expansion in invaded range. Species distribution limited primarily by climatic factors.

#### Assumptions

*Model assumptions:* Species is in equilibrium with climate in sampled areas; occurrences represent suitable habitat; pseudo-absences adequately represent unsuitable conditions; climatic variables capture main environmental constraints.

#### Algorithms

*Modelling techniques:* Ensemble modelling using 12 algorithms from ‘biomod2’ package: Artificial Neural Networks (ANN), Classification Tree Analysis (CTA), Flexible Discriminant Analysis (FDA), Generalized Boosted Models (GBM), Generalized Linear Models (GLM), Multivariate Adaptive Regression Splines (MARS), MAXNET, Random Forest (RF), Support Vector Machines (SVM), XGBOOST, and additional techniques. Six ensemble methods: Mean (EMmean), Median (EMmedian), Coefficient of Variation (EMcv), Confidence Interval (EMci), Committee Averaging (EMca), Weighted Mean (EMwmean)

*Model complexity:* Default complexity settings for each algorithm in ‘biomod2’ package; 3 variable importance permutations; 10-fold cross-validation

*Model averaging:* Six ensemble modelling techniques applied to models with TSS ≥ 0.7. Best performers: EMmedian for native range (TSS=0.824, ROC=0.969, Kappa=0.591); EMca for expanded range (TSS=0.812, ROC=0.971, Kappa=0.641). EMmedian used for final visualizations for consistency.

#### Workflow

*Model workflow:* (1) Data collection and compilation from multiple sources; (2) Data cleaning and spatial thinning (10 km, 100 repetitions); (3) PCA and VIF analysis for variable selection; (4) Pseudo-absence generation (3x presence points); (5) Model fitting with 12 algorithms using ‘biomod2’; (6) Model evaluation (TSS, ROC, Kappa, Boyce); (7) Model selection (TSS ≥ 0.7); (8) Ensemble modelling (6 techniques); (9) Continuous Boyce Index (CBI) validation; (10) Future climate projections; (11) Niche overlap and dynamics analysis using ‘ecospat’

#### Software

*Software:* R (R Core Team 2017), RStudio 2023.12.1+; QGIS 3.28.6; Adobe Illustrator CS6; Adobe Photoshop CS5. R packages: ‘biomod2’ (Guéguen et al. 2025), ‘ecospat’ (Di Cola et al. 2017), ‘spThin’ (Aiello-Lammens et al. 2015), ‘ade4’ (Dray and Dufour 2007), ‘usdm’ (Naimi et al. 2014)

*Code availability:* available in supplementary material

*Data availability:* *Toxomerus floralis*: GBIF occurrence data: https://doi.org/10.15468/dl.84a7g3 (accessed 22 July 2025); iNaturalist: https://www.inaturalist.org/; WorldClim 2.1: https://www.worldclim.org/; *Cyperus rotundus*: https://doi.org/10.15468/dl.xqaym4; *Mitracarpus hirtus:* https://doi.org/10.15468/dl.dqh46y; Museum specimen data available in supplementary material

## Data

#### Biodiversity data

*Taxon names:* *Toxomerus floralis* (Fabricius, 1798)

*Taxonomic reference system:* Thompson & Thompson (2006), Borges & Couri (2009), Thompson (2013)

*Ecological level:* species level

*Data sources:* Field sampling by authors; 24 museum collections (AMGS, AMNH, ASPC, BMSA, CAS, CIRAD, CNC, CSCA, DMSA, FSUNS, icipe, IITA, RBINS, RMCA, MZH, MZLU, NHMUK, NHRS, NMKE, NMSA, RMNH, SAMC, USNM, ZFMK); GBIF database (https://doi.org/10.15468/dl.84a7g3); iNaturalist citizen science platform.

*Sampling design:* Retrospective observational study combining museum specimens, targeted field sampling, and citizen science observations. No standardized sampling protocol across data sources.

*Sample size:* Initial: 1229 GBIF records: 632 total records (451 native, 181 invaded); After spatial thinning: 363 final records (235 native, 128 invaded, 363 expanded);

*Scaling:* none

*Data filtering:* Filtered to valid geographic coordinates only; removed records with coordinate uncertainty >10 km to match environmental variable resolution; removed outliers identified through PCA analysis.

*Absence data:* Pseudo-absences generated using BIOMOD_FormatingData function from ‘biomod2’ package. Number generated: 3x presence points (Native=705, Invaded=384, Expanded=1089). Strategy: Random selection within study area extent, excluding cells with presence records. Surface range envelope approach not used as fundamental niche not fully understood due to ongoing range expansion.

*Background data:* Global extent defined by WorldClim 2.1 bioclimatic variable coverage. Background area used for pseudo-absence generation encompasses entire extent of environmental data.

#### Data partitioning

*Training data:* 80% of data used for model training. Partitioning performed using randomized cross-validation strategy with 10 validation sets for each model and pseudo-absence dataset.

*Validation data:* 20% of data used for model validation. 10-fold cross-validation with random partitioning for each of 12 algorithms across 3 pseudo-absence sets, totaling 1332 model runs per range (native, expanded).

*Test data:* No independent test dataset. Model performance evaluated using cross-validation and Continuous Boyce Index (CBI) on validation data.

#### Predictor variables

*Predictor variables:* Final 5 variables: Bio2 (Mean Diurnal Range), Bio3 (Isothermality), Bio8 (Mean Temperature of Wettest Quarter), Bio15 (Precipitation Seasonality - Coefficient of Variation), Bio18 (Precipitation of Warmest Quarter). Initially considered all 19 WorldClim bioclimatic variables.

*Data sources:* WorldClim 2.1 (Fick & Hijmans 2017) - https://www.worldclim.org/

*Spatial extent:* The accessible area for native (xmin: -129.3333, xmax: -27.0000, ymin: -60.3333, ymax: 40.3333) and invaded (xmin: -23.750, xmax: 69.250, ymin: -42.6667, ymax: 40.8333).

*Spatial resolution:* 5 arc minutes (~9.28 km at equator)

*Coordinate reference system:* WGS84 geographic coordinate system (latitude-longitude)

*Temporal extent:* 30-year climatological averages (1970–2000)

*Data processing:* Variables extracted from WorldClim 2.1 rasters; combined into raster stacks for modelling; standardized spatial resolution and extent across all variables

#### Transfer data

*Data sources:* WorldClim future climate projections derived from Global Climate Models (GCMs): Earth3-Veg, MRI-ESM2-0 and MPI-ESM1-2. GCM selection based MESS analysis.

*Spatial extent:* Global coverage, same as current climate data

*Spatial resolution:* 5 arc minutes (~9.28 km at equator)

*Temporal extent:* Four future periods: 2021–2040, 2041–2060, 2061–2080, 2081–2100

*Models and scenarios:* Three GCMs: Earth3-Veg, MRI-ESM2-0 and MPI-ESM1-2. Two Shared Socioeconomic Pathways (SSPs): SSP2-4.5 (middle of the road scenario with medium challenges to mitigation and adaptation), SSP5-8.5 (fossil-fueled development with high mitigation challenges, low adaptation challenges); Total: 48 future projections (3 GCMs x 2 SSPs x 4 time periods x 2 range models).

## Model

*Variable pre-selection:* started with all 19 WorldClim 2.1 bioclimatic variables as candidates based on known importance of temperature and precipitation for insect distributions.

#### Multicollinearity

*Multicollinearity:* Addressed using two complementary approaches: (1) PCA to identify orthogonal variables and visualize environmental space occupation; (2) VIF analysis using ‘usdm’ package - vifcor() function with maximum linear correlation threshold of 0.9, vifstep() function to calculate VIF and exclude variables exceeding threshold of 10. Process resulted in retention of 5 uncorrelated variables from initial 19 Bioclimatic variables.

#### Model settings

*Model settings (fitting):* ‘biomod2’ package BIOMOD_Modeling function with default settings for each of 12 algorithms. 80/20 training-validation split; 10-fold randomized cross-validation; 3 pseudo-absence datasets; 3 permutations per variable for importance; total 1332 models per range. Separate models fitted for native range (235 occurrences, 705 pseudo-absences) and expanded range (363 occurrences, 1089 pseudo-absences).

*Model settings (extrapolation):* Models projected to: (1) global extent for current climate; (2) future climates (32 projections: 2 GCMs x 2 SSPs x 4 time periods); (3) geographic regions outside training extent (Eastern Palaearctic, Indomalayan, Australasian). No clamping or truncation applied.

#### Model estimates

*Coefficients:* Not applicable for ensemble models. Individual model coefficients not reported, as ensemble approach prioritizes predictive performance over parameter interpretation.

*Variable importance:* Calculated using 3 permutations per variable for each model. Native range ensemble (EMmedian): Bio18=42% (±10.9%), Bio2=33% (±10.5%). Expanded range ensemble (EMca): Bio2=62% (±10.9%), Bio18=31% (±10.5%). Bio18 and Bio2 consistently most important across all ensemble techniques.

#### Analysis and Correction of non-independence

*Spatial autocorrelation:* Spatial thinning applied spThin package at 10 km resolution with 100 repetitions on random selections to ensure representative final dataset. Thinning distance matched environmental variable resolution (5 arc-minutes = 9.28 km). No additional spatial autocorrelation correction in models.

#### Threshold selection

*Threshold selection:* Models evaluated using threshold-independent metrics (ROC, Continuous Boyce Index (CBI)) and threshold-dependent metrics (TSS, Kappa). TSS threshold of 0.7 was used for model selection.

## Assessment

#### Performance statistics

*Performance on training data:* Not separately reported. Models evaluated using cross-validation which includes training performance.

*Performance on validation data:* Native range ensemble (EMmedian): TSS=0.824, ROC=0.969, Kappa=0.591, CBI Spearman Rs=0.982. Expanded range ensemble (EMca): TSS=0.812, ROC=0.971, Kappa=0.641, CBI Rs=0.997. Expanded range (EMmedian, used for visualization): TSS=0.805, ROC=0.964, Kappa=0.561. Single models before ensemble: Native mean TSS=0.73 (n=108 retained); Expanded mean TSS=0.72 (n=32 retained).

*Performance on test data:* No independent test data. Continuous Boyce Index (CBI) used as additional validation metric on holdout data. CBI calculated using ecospat.boyce function with Spearman rank correlation of predicted-to-expected ratio.

#### Plausibility check

*Response shapes:* Response curves generated for all five bioclimatic variables (Bio2, Bio3, Bio8, Bio15, Bio18). Bio2: occurrence probability declines rapidly when mean diurnal range exceeds ~8°C (native) or ~10.5°C (expanded). Bio18: highest occurrence at 600–650mm precipitation in warmest quarter, with native range showing plateau then decline, expanded range showing stable plateau at higher precipitation. Curves indicate preference for humid tropical/subtropical zones.

*Expert judgement:* Model projections reviewed for biological plausibility. Predicted distribution overlaps with known host plant distributions (*Cyperus rotundus, Mitracarpus hirtus*). Predictions consistent with observed rapid expansion in Afrotropical Region since 2013. Unsuitable predictions for semi-arid regions (Namibia, Botswana) align with species' apparent moisture requirements, though recent Botswana observation (2025) indicates some model conservatism.

## Prediction

#### Prediction output

*Prediction unit:* Probability of occurrence (continuous 0–1 scale) per grid cell (5 arc-minute resolution). Binary presence/absence maps also generated using threshold.

*Post-processing:* Prediction outputs exported to QGIS 3.28.6 for final mapping and visualisation.

#### Uncertainty quantification

*Input data uncertainty:* Not explicitly quantified. Spatial thinning (100 repetitions) helps reduce sampling bias. Pseudo-absence uncertainty addressed by generating 3 sets of pseudo-absences and running models across all sets. Additionally any older records without uncertainty estimates or with uncertainty exceeding the spatial resolution were excluded from analyses.

*Novel environments:* Niche dynamics analysis using ‘ecospat’ package show 23.6% niche expansion in invaded range (p=0.048), indicating species can establish in novel environments and environmental conditions. Schoener's D=0.275 and Hellinger's I=0.512 indicate minor to moderate niche overlap between ranges, suggesting some environmental differences.
